# Evolutionary genomics of three agricultural pest moths reveals rapid evolution of host adaptation and immune-related genes

**DOI:** 10.1093/gigascience/giad103

**Published:** 2024-01-02

**Authors:** Yi-Ming Weng, Pathour R Shashank, R Keating Godfrey, David Plotkin, Brandon M Parker, Tyler Wist, Akito Y Kawahara

**Affiliations:** McGuire Center for Lepidoptera & Biodiversity, Florida Museum of Natural History, University of Florida, Gainesville, FL 32611, USA; McGuire Center for Lepidoptera & Biodiversity, Florida Museum of Natural History, University of Florida, Gainesville, FL 32611, USA; Division of Entomology, ICAR-Indian Agricultural Research Institute, Pusa, New Delhi 110012, India; McGuire Center for Lepidoptera & Biodiversity, Florida Museum of Natural History, University of Florida, Gainesville, FL 32611, USA; McGuire Center for Lepidoptera & Biodiversity, Florida Museum of Natural History, University of Florida, Gainesville, FL 32611, USA; McGuire Center for Lepidoptera & Biodiversity, Florida Museum of Natural History, University of Florida, Gainesville, FL 32611, USA; Agriculture and Agri-Food Canada, Saskatoon, SK, S7N 0×2, Canada; McGuire Center for Lepidoptera & Biodiversity, Florida Museum of Natural History, University of Florida, Gainesville, FL 32611, USA

**Keywords:** Gelechiidae, twirler moths, genome assembly, host adaptation, detoxification, immunity, tomato leafminer, tomato pinworm, goosefoot groundling moth

## Abstract

**Background:**

Understanding the genotype of pest species provides an important baseline for designing integrated pest management (IPM) strategies. Recently developed long-read sequence technologies make it possible to compare genomic features of nonmodel pest species to disclose the evolutionary path underlying the pest species profiles. Here we sequenced and assembled genomes for 3 agricultural pest gelechiid moths: *Phthorimaea absoluta* (tomato leafminer), *Keiferia lycopersicella* (tomato pinworm), and *Scrobipalpa atriplicella* (goosefoot groundling moth). We also compared genomes of tomato leafminer and tomato pinworm with published genomes of *Phthorimaea operculella* and *Pectinophora gossypiella* to investigate the gene family evolution related to the pest species profiles.

**Results:**

We found that the 3 solanaceous feeding species, *P. absoluta, K. lycopersicella*, and *P. operculella*, are clustered together. Gene family evolution analyses with the 4 species show clear gene family expansions on host plant–associated genes for the 3 solanaceous feeding species. These genes are involved in host compound sensing (e.g., gustatory receptors), detoxification (e.g., ABC transporter C family, cytochrome P450, glucose-methanol-choline oxidoreductase, insect cuticle proteins, and UDP-glucuronosyl), and digestion (e.g., serine proteases and peptidase family S1). A gene ontology enrichment analysis of rapid evolving genes also suggests enriched functions in host sensing and immunity.

**Conclusions:**

Our results of family evolution analyses indicate that host plant adaptation and pathogen defense could be important drivers in species diversification among gelechiid moths.

## Background

Gelechiidae are a diverse family of Lepidoptera comprising more than 4,700 species [1, 2]. Some species, such as *Phthorimaea absoluta* (tomato leafminer), *Keiferia lycopersicella* (tomato pinworm), and *Phthorimaea operculella* (potato tuber moth), are notorious agricultural pests that could cause more than a billion US dollars of annual agricultural damage globally [3–10]. The 3 gelechiids all use solanaceous plants as larval hosts, but *P. absoluta* and *K. lycopersicella* feed primarily on tomato while *P. operculella* prefers potato. These species, especially the 2 *Phthorimaea* species, are found invading many nonnative regions, including Asia, Europe, and Africa. Research on these moths has focused largely on their host preference, identification, and management. Despite their importance as major global pests to agriculture, their genomic framework and the evolutionary process of host plant preference in insect pests are still poorly understood (but see [11]).

Host selection and host use in insects are determined by a series of physiological processes, including host plant compound sensing, detoxification, and nutrient digestion. Several genes are thought to be involved in these processes that affect host selection [12]. Genes associated with sensing phytocompounds include olfactory receptors, gustatory receptors, ionotropic receptors, odorant-binding proteins, and chemosensory proteins. Genes associated with detoxification include cytochrome P450 (P450), ATP-binding cassette transporter (ABC), and glutathione S-transferase, and genes associated with digestion include serine protease and beta-fructo-furanosidase [13–21]. A crucial question in understanding pest evolution is how these genes evolved among pest species and their relatives. Whole-genome sequencing of pest species has shown great promise for revealing the evolutionary processes that led to the formation of a pestiferous species. For example, recent studies on the genomic evolution of agricultural pests, with subsequent comparative genomics analyses such as orthology, gene family evolution, selected region detections, and structural variant analyses, have identified putative genetic bases of their ecological features or pest species profiles [22–25].

Despite the diversity of gelechiid moths, the many studies on the impact of gelechiids to agriculture, and the release of nearly a thousand Lepidoptera genome assemblies in GenBank thus far [26], only a few gelechiid genome assemblies are publicly available [11, 27, 28]. Considering its high species diversity and economic importance, more attention and efforts on genomic data accumulation and exploration are required for further understanding the evolution of this moth family. In this study, we sequenced and assembled the genomes of 3 gelechiid moth pests, *Keiferia lycopersicella, Phthorimaea absoluta*, and *Scrobipalpa atriplicella*, to examine their genomic features and how they relate to host preference. Specifically, we investigate how rapidly evolving genes are correlated with host preference and life history.

## Methods

### Sample information and sequencing

Three gelechiid moth species (*K. lycopersicella* [NCBI:txid1511203], *P. absoluta* [NCBI:txid702717], *S. atriplicella* [NCBI:txid687131]) were collected from laboratory colonies at University of California, Davis, USA; Khumaltar, Lalitpur, Nepal; and the Saskatoon Research and Development Centre of Agriculture and Agri-Food, Canada, respectively. Genomic DNA from one moth of each species was extracted from the whole moth (larva) using the DNA isolation protocol of the OmniPrep Genomic DNA Extraction Kit (G-Biosciences). For *S. atriplicella*, we encountered sequencing interference for several library samples. Therefore, we amplified genomic DNA with illustra™ GenomiPhi V2 DNA Amplification Kits (Cytiva), and the amplified DNA was used to replace the native DNA extracted from the tissue. The genomic and amplified DNA samples were subsequently used to perform fragment size selection and sample purification with the DNeasy PowerClean CleanUp Kit, Qiagen before library preparation. Libraries were sequenced with a single SMRT cell in the Pacific Biosciences (PacBio) Sequel IIe system. The DNA cleanup, library construction, and sequencing steps were performed in the Interdisciplinary Center for Biotechnology Research at the University of Florida. The high-fidelity (HiFi) sequences are deposited in NCBI (BioProject accession number: PRJNA932016; SRA sample accession: SRR23497930, SRR23497929, and SRR23497928).

### Genome size and sequence coverage estimations

To verify read quality, we first assessed the HiFi sequence quality using FastQC v 0.11.7 (RRID:SCR_014583) to summarize read profiles [29]. The genome size and sequence coverage were estimated with 2 methods. First, we counted *k*-mers and calculated the *k*-mer density distribution for the HiFi reads using K-Mer Counter (kmc) v.3.2.1 (RRID:SCR_001245) with a *k*-mer size of 31 nucleotides. Density distributions were subsequently submitted to GenomeScope v2.0 online tool (RRID:SCR_017014) [30] with the default setting for diploid species to estimate the genome size, heterozygosity, sequence coverage, and other genomic profiles (Supplementary Fig. S1). Second, we mapped HiFi reads to final assemblies to estimate genome size and sequence coverage. This process was conducted in the program ModEst (backmap.pl v 0.5) [31]. Estimated genome sizes and read coverages from GenomeScope were used to certify autodetected estimates from the hifiasm assembler (see next section) to ensure the accuracy of autodetected assembling assumptions [32].

### Genome assembly, quality assessment, and nontarget sequence removal

We used hifiasm v 0.16.1 (RRID:SCR_021069) to assemble the genome from HiFi reads using default settings, except for reads of *P. absoluta*, for which we applied a 2 (-l 2) purging level to keep a greater number of haplotigs for downstream purging. We kept more haplotigs because the sequence coverage for this species was low, and it generated the best assembly evaluated by N50 and BUSCO v 5.3.0 (RRID:SCR_015008) completeness (based on the lepidoptera_odb10 database) [33, 34]. We also applied the haplotig purging pipeline to remove duplicated haplotigs [35]. For *K. lycopersicella* and *S. atriplicella*, we first mapped the HiFi reads to the assemblies with Minimap v. 2.21 (RRID:SCR_008103) and sorted using samtools v 1.15 (RRID:SCR_002105) [36, 37]. The sorted mapped reads were subsequently used to draw the density distribution histogram of coverage using “hist” function in the purge_haplotigs pipeline (RRID:SCR_017616) [34]. The histogram was used to identify the peaks of homozygous and heterozygous reads and the low point between the peaks. These values were then used to define the aggressiveness of the purging. Finally, we used low- and high-coverage cutoffs to purge the duplicated contigs. Other parameters were kept default as suggested by purge_haplotigs pipeline. For *P. absoluta*, since its sequence depth is relatively low (see Results), we used the Illumina short reads published by [28] to perform the purge_haplotigs. Specifically, we ran Hifiasm with less aggressive purging (l -2) to allow more duplicated haplotypes in the assembly for the haplotig purging pipeline and used the short-read coverage histogram to define the peaks. To identify potential nontarget sequences in assemblies, we created blobplots using blobtools (RRID:SCR_017618) to visualize the distribution of GC content and read coverage for contigs [38]. To determine read coverage, we aligned HiFi reads to the assembly using minimap2 [36]. To assign taxonomy to reads, we used blastn (RRID:SCR_001598) to blast contigs against the NCBI nt database with an e-value cutoff of 1e-25. Contigs assigned to nonarthropods with deviating GC content and sequence coverage were determined to be nontarget sequences and removed from assemblies (Supplementary Fig. S2). A BUSCO score using the lepidoptera_odb10 database was calculated to evaluate the completeness of each assembly (Table 1). Genome assemblies of the 3 species are available through NCBI (BioProject accession number: PRJNA932016).

**Table 1:** Assembly statistics of the 3 newly sequenced gelechiid moth species, compared to statistics of the published *Phthorimaea absoluta* v1 assembly. BUSCO results from the *Phthorimaea absoluta* v1 assembly have been reanalyzed using BUSCO v5. (C: Complete; S: Complete and single-copy; D: Complete and duplicated)

	*Phthorimaea absoluta* v1	*Phthorimaea absoluta* v2	*Keiferia lycopersicella*	*Scrobipalpa atriplicella*
Number of contigs	51,398	688	61	7,092
Total length	906,539,853	652,703,157	443,647,192	301,148,843
GC content	38.11%	38.45%	38.86%	36.87%
Contig N50	97,121	1,614,219	14,556,016	51,599
Contig L50	1,787	116	12	1,805
Genome BUSCO complete (C)	C:90.3% [S:67.1%, D:23.2%]	96.2% [S:82.5%, D:13.7%]	96.6% [S:95.5%, D:1.1%]	C:73.3% [S:69.7%, D:3.6%]
Genome BUSCO fragmented (F)	3.4%	0.5%	0.7%	2.8%
Genome BUSCO missing (M)	6.3%	3.3%	2.7%	23.9%
Repeat percentage	—	54.4%	48.22%	32.83%
Number of protein coding genes	—	19,106	15,405	14,647
Gene model BUSCO (C)	—	93.2% [S:75.9%, D:17.3%]	93.2% [S:91.7%, D:1.5%]	70.2% [S:60.6%, D:9.6%]
Gene model BUSCO (F)	—	1.3%	0.7%	3.4%
Gene model BUSCO (M)	—	5.5%	6.1%	26.4%
Number of monoexonic genes	—	2,568	2,040	1,600
Reference	Tabuloc et al. [36]	This study	This study	This study

### Gene models and annotations

In the genome annotation pipeline, we first identified repeat regions using RepeatModeler2 (RRID:SCR_015027) [39]. The genome assemblies were soft-masked with repeats from 3 lines of evidence, including simple and short repeats, the identified repeats from RepeatModeler2, and the evidence from the lepidopteran repeat database in Repbase using RepeatMasker (RRID:SCR_012954) with the blast tool RMBlast (RRID:SCR_022710) [40, 41]. The Braker2 gene prediction pipeline (RRID:SCR_018964) was applied to soft-masked genomes [37, 42–47]. For *K. lycopersicella* and *S. atriplicella*, we used arthropod protein sequences from orthoDB (RRID:SCR_011980) (odb10_arthropoda) in the ProtHint pipeline (RRID:SCR_021167) to generate hints to train GeneMark-EP+ (RRID:SCR_011930) [48] and predict gene models alone with the Augustus (RRID:SCR_008417). For *P. absoluta*, we also included published RNA sequences to train the gene model [49]. Specifically, we ran Braker2 pipeline twice (one with protein and one with RNA) and used TSEBRA [50] with default settings to integrate the 2 models. To further refine models for the 3 species, we removed genes identified solely by Augustus  *ab initio* prediction without hint supports (e.g., introns, start and stop codons) from the protein database using the Python script “selectSupportedSubsets.py” provided by Braker2. Final gene models were evaluated using a BUSCO protein model with the lepidoptera_odb10 database. Gene model profiles, including the monoexonic rate and sequence lengths of gene, intron, and exon, were summarized using gFACs v1.0.0 (RRID:SCR_022017) [51] (Supplementary Table S1).

For functional annotations, we first annotated gene function by blasting transcript sequences from the Braker2 pipeline to the RefSeq nonredundant protein database and Swiss-Prot arthropodan protein database (Reviewed UniPort database) using the blastp function in Diamond v2.0.9 (RRID:SCR_016071) [44]. Additionally, we performed default InterProScan (RRID:SCR_005829) annotation, which integrates 14 member databases, including Pfam and Panther [52]. For Gene Ontology (GO) terms and KEGG pathway annotations (RRID:SCR_012773), we queried transcript sequences to the PANNZER webserver (Protein annotation with *z*-score) [53] and KEGG automatic annotation server [54] with bidirectional best hits.

### Phylogeny and gene evolution

To explore the evolution of the 3 gelechiid moths and their genes, we created a phylogeny using 2 additional published genomes of Gelechiidae: *P. operculella* and *Pectinophora gossypiella*. We used the genome of *Hyposmocoma kahamanoa* as an out-group, as this species belongs to a moth family closely related to Gelechiidae (Cosmopterigidae) [55, 56]. Published genome assembly of *P. operculella* (GCA_024,500,475.1) was downloaded from NCBI GenBank while those of *P. gossypiella* (GCF_024,362,695.1) and *H. kahamanoa* (GCF_003,589,595.1) were downloaded from the NCBI Reference Sequence (RefSeq) Database [57]. We performed the same BUSCO approach using the lepidoptera_odb10 database to obtain compatible single-copy amino acid orthologs for these 3 species [32]. The final data matrix contained 4,876 single-copy orthologs that contained at least 2 in-group species and the *H. kahamanoa* out-group (385 orthologs did not fit these parameters and were removed). Sequences of each ortholog were aligned using default settings in MAFFT version 7.490 (RRID:SCR_011811) [58].

Phylogeny of these 5 gelechiid moth species was constructed using the concatenated sequences from the BUSCO single-copy gene alignments. We assigned a single-substitution model (Q.insect+FO+G4 substitution model, the Q matrix estimated for insects) to the alignment and built a maximum likelihood tree in IQ-tree v 2.1.3 (RRID:SCR_017254) [59–62]. Branch supports were calculated using ultrafast bootstrap [63] and SH-aLRT [64, 65]. Since the genome assembly of *S. atriplicella* is less complete (see Results), we also constructed phylogeny with the 4 gelechiid moth species (excluding *S. atriplicella*) and *H. kahamanoa* (out-group) for gene family analysis (see below) to avoid the noise from the incomplete gene model of *S. atriplicella* with the same tree-building approach.

To investigate gene family evolution, we inferred an ultrametric tree from the concatenated sequence species tree using treePL with default settings [66]. For gene family identification, we employed OrthoFinder v2.5.2 (RRID:SCR_017118) using the primary isoform of the annotated gene models from each of the 5 species (4 gelechiid species and 1 out-group) [67]. Gene models of *K. lycopersicella* and *P. absoluta* were predicted from the Braker2 pipeline while the other 3 gene models were directly downloaded from appropriate databases. In OrthoFinder, we chose gene families as defined by phylogenetic hierarchical orthogroups (HOGs), an approach that is thought to be more accurate than similarity-based methods [67]. For each gene family, the HOGs’ gene-counting matrix and ultrametric tree were used to estimate repertoire size changes in cafe v 5.0.0 (RRID:SCR_018924) [68]. We extracted HOGs under rapid repertoire size expansion and contraction, with the significance level set to 0.01 and branch lengths calculated from the ultrametric tree. For each gene associated with these HOGs, we used the top annotated function (lowest e-value) from InterProScan to represent the gene function.

For the HOGs with significant rapid expansion and contraction, we assessed their gene functions and GO terms using InterProScan annotations. To standardize annotations, we reannotated gene functions for the 3 downloaded gene models (*P. operculella, P. gossypiella*, and *H. kahamanoa*) using default settings in InterProScan [68]. For associated GO terms, we performed enrichment analysis using the R package topGO 2.40.0 (RRID:SCR_014798) [69] with a significance level of 0.05 for both fisher classic and weight01 algorithms.

## Results

### Genome assemblies and annotations

To assemble genomes of *K. lycopersicella, P. absoluta*, and *S. atriplicella*, we used 3.8, 2.2, and 2.9 million PacBio HiFi reads (mean read lengths of 5.5, 4.9, and 7.6 kbp, respectively) corresponding to the estimated read coverage of 50×, 20×, and 37×, respectively. After haplotig removal, considerable reductions in the number of contigs were found while assembled sizes and BUSCO completeness remained nearly consistent, indicating that smaller duplicated contigs were removed. From the assemblies of *K. lycopersicella* and *S. atriplicella*, we identified nontarget sequences contributing to a small portion of the assemblies. In *K. lycopersicella*, a 10-kbp contig was blasted to Streptophyta while in *S. atriplicella*, 15 small contigs (total 380 kbp) were blasted to Proteobacteria. After removing these nontarget contigs, 443.65 Mb from 61 contigs, 652.7 Mb from 688 contigs, and 301.15 Mb from 7,092 contigs were found in the assemblies of *K. lycopersicella, P. absoluta*, and *S. atriplicella*, respectively. BUSCO scores for these assemblies are shown in Table 1. Estimated genome sizes from GenomeScope for *K. lycopersicella, P. absoluta*, and *S. atriplicella* were 396, 514, and 276 million base pairs (Mb)—much smaller than our assemblies and likely due to the exclusion of extremely high number of *k*-mers from the repeats in the genomes. Estimating genome size with ModEst resulted in 422, 1,040, and 344 Mbs with peak coverages at 49×, 10×, and 55× for *K. lycopersicella, P. absoluta*, and *S. atriplicella*, respectively (Supplementary Table S2). The estimated genome and assembly sizes of *P. absoluta* based on ModEst were nearly twice that of GenomeScope, and this discrepancy was likely due to different estimated sequence coverage.

For gene annotation, we first annotated repeats using RepeatModeler2 [39], and *P. absoluta* showed the highest proportion of repeats (54.4%), followed by *K. lycopersicella* (48.22%) and *S. atriplicella* (32.83%). Soft-masked genomes were used to run the Braker2 pipeline with protein evidence for *K. lycopersicella* and *S. atriplicella*, resulting in 15,405 and 14,647 genes, respectively [43]. For *P. absoluta*, we used both protein and RNA sequence evidence to predict the gene model. After removing genes without hint support, the gene model with 19,106 genes was used for functional annotation. BUSCO scores for these gene models reflect their assembly features, including the higher duplication rate in *P. absoluta* and higher missing rate in *S. atriplicella* (Table 1).

### Phylogeny and gene family evolution

**Figure 1:**
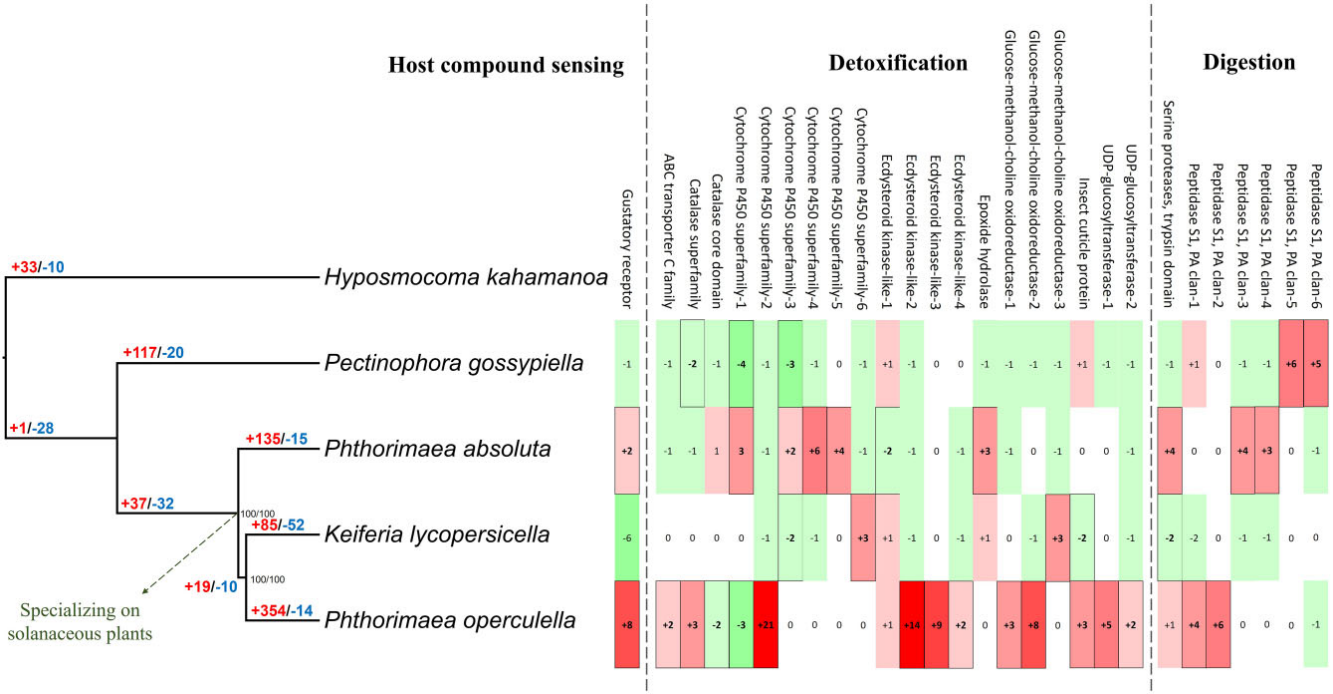
(left) Maximum likelihood tree of 4 gelechiid species from a concatenated supermatrix analysis of 4,876 single-copy genes, presented alongside a color-coded number of rapidly evolving gene families (red: expanding, blue: contracting). The tree is rooted with *Hyposmocoma kahamanoa* (Gelechioidea: Cosmopterigidae). Nodes are labeled with branch supports (ultrafast bootstrap/SH-aLRT). (right) The list of rapidly evolving gene families that are associated with host plants includes a host compound–sensing gene family, 20 detoxification genes, and 7 digestion-related genes. Numbers in color-coded cells represent repertoire size change in corresponding branches on the tree on the left, and gene-family functions are shown at the top of columns. The significant repertoire size changes are marked with outside borders on the cell. References supporting categorizations of gene function are provided in Supplementary Table S5.

The maximum likelihood tree, derived from the concatenated supermatrix, shows that *K. lycopersicella* and *P. operculella* are most closely related (Fig. 1). *Phthorimaea absoluta*, another species feeding on solanaceous hosts, is the sister species to *K. lycopersicella* and *P. operculella*. It is noteworthy that *P. absoluta* was previously and widely recognized as *Tuta absoluta*, indicating a need for a comprehensive phylogenomic analysis on this group. *S. atriplicella*, an amaranthaceous feeder, is recovered as the sister taxon to the other 3 members of subfamily Gelechiinae in the 6-species phylogeny (Supplementary Fig. S3). Finally, *P. gossypiella*, a member of subfamily Apatetrinae, is the sister taxon to all 4 Gelechiinae species in the phylogeny, supporting the current taxonomic arrangement [70] (Fig. 1).

We identified 14,384 HOGs in the protein sequences of the 6 species, and 809 HOGs were found to evolve rapidly at least in 1 branch along the ultrametric tree (Supplementary Table S3). Gene family evolution analyses showed a general pattern of rapid expansions at the tips of the tree and rapid contractions along internal branches (Fig. 1). Specifically, 85 and 52 HOGs were identified in *K. lycopersicella* with rapid repertoire size expansion and contraction, respectively. Among these HOGs, 67 are annotated with gene functions where 5 are putatively involved in host plant adaptation (glucose-methanol-choline oxidoreductase, cytochrome P450 superfamily, insect cuticle proteins, and trypsin family serine proteases), 37 involved in immunity (*PiggyBac* transposable elements, retrotransposon *Pao*-related genes, serpin superfamilies, and Toll-like receptors) (Supplementary Tables S4 and S5).

For *P. operculella*, 368 HOGs (310 expansions and 66 contractions) were identified to evolve rapidly where 166 HOGs (158 expansions and 8 contractions) are annotated with gene functions. From these HOGs, we found that 16 are putatively associated with host plant adaptation, including gustatory receptor, catalase superfamily, cytochrome P450 superfamily, ecdysteroid kinases, glucose-methanol-choline oxidoreductase, insect cuticle proteins, and UDP-glucuronosyl. We also found 68 immune-related associated HOGs (gamma interferon–related gene, pacifastin domain, serpin superfamily, *PiggyBac* transposable element, retrotransposon *Pao*-related genes, and *Ty3* transposon) and 1 pheromone signaling-related gene (CD36 family). For *P. absoluta*, 150 HOGs (135 expansions and 15 contractions) were identified to evolve rapidly, and among them, 102 HOGs (94 expansions and 8 contractions) are annotated with gene functions. These HOGs include 9 host plant adaptation-related genes (cytochrome P450 superfamilies, epoxide hydrolase, ecdysteroid kinases, and serine proteases) and 39 immune-related genes (immunoglobulin, pacifastin, retrotransposon *Pao*-related genes, *PiggyBac* transposable element, and Toll-like receptor). Finally, we found 137 (135 expansions and 20 contractions) rapidly evolving HOGs along the branch of *P. gossypiella*, where 101 HOGs have gene annotations (90 expansions and 11 contractions). Among them, 36 HOGs are associated with immunity (*PiggyBac* transposable elements, retrotransposon *Pao*-related genes, and Toll-like receptors). We also found 5 (2 expansions and 3 contractions) host plant adaptation-related genes (cytochrome P450 superfamilies, catalase superfamily, and peptidase family S1). A summary of HOGs with putative functions in immunity and host adaptation is listed in Supplementary Tables S4 and S5, respectively.

### GO enrichment analyses

From genes that were identified to be rapidly evolving, we found a handful of biological function terms that were enriched from the rapidly evolving genes (Table 2). This includes sensory perception of taste (GO:0,050,909), Toll-like receptor signaling pathway (GO:0,002,224), and immune response (GO:0,006,955), DNA integration (GO:0,015,074), and plasma membrane phospholipid scrambling (GO:0,017,121). All the enriched terms, including those terms passing through Fisher classic but not weight01 threshold, are listed in Supplementary Table S6.

**Table 2:** Enriched GO terms from the rapidly evolving genes of the 5 gelechiid species in this study

	GO term ID	Biological function	*P*_weight_	*P*_classic_
*K. lycopersicella*	GO:0,007,275	Multicellular organism development	5.52E-09	5.52E-09
	GO:0,007,304	Chorion-containing eggshell formation	4.31E-14	4.31E-14
*P. operculella*	GO:0,006,313	Transposition, DNA-mediated	0.0161611	0.0161611
	GO:0,006,508	Proteolysis	4.955E-05	6.116E-05
	GO:0,006,979	Response to oxidative stress	0.0028518	0.0028518
	GO:0,015,074	DNA integration	2.156E-06	2.156E-06
	GO:0,050,909	Sensory perception of taste	6.504E-10	5.851E-09
*P. absoluta*	GO:0,000,723	Telomere maintenance	0.046281	0.046281
	GO:0,002,224	Toll-like receptor signaling pathway	0.023407	0.023407
	GO:0,006,313	Transposition, DNA mediated	0.0015755	0.0015755
	GO:0,006,508	Proteolysis	0.0035861	0.0035861
	GO:0,006,955	Immune response	0.046281	0.046281
	GO:0,007,275	Multicellular organism development	3.635E-05	3.635E-05
	GO:0,007,304	Chorion-containing eggshell formation	3.121E-08	3.121E-08
	GO:0,010,923	Negative regulation of phosphatase activity	1.247E-06	1.247E-06
	GO:0,048,015	Phosphatidylinositol-mediated signaling	0.046281	0.046281
*P. gossypiella*	GO:0,006,310	DNA recombination	0.0009534	0.0009534
	GO:0,006,334	Nucleosome assembly	2.504E-29	2.504E-29
	GO:0,006,357	Regulation of transcription by RNA polymerase II	0.03104	0.03104
	GO:0,006,418	tRNA aminoacylation for protein translation	0.0002912	0.0002912
	GO:0,006,486	Protein glycosylation	0.0024002	0.0024002
	GO:0,006,915	Apoptotic process	0.0006649	0.0006649
	GO:0,007,275	Multicellular organism development	0.0099958	0.0099958
	GO:0,007,304	Chorion-containing eggshell formation	0.0005445	0.0005445
	GO:0,015,074	DNA integration	5.079E-20	5.079E-20
	GO:0,017,121	Plasma membrane phospholipid scrambling	2.236E-09	2.236E-09

## Discussion

### Genome assembly quality and its implications for gene family evolution

Long-read sequencing technologies such as PacBio and Oxford Nanopore Technologies have provided a promising future for *de novo* assemblies of high-quality genomes for nonmodel species [71, 72]. These recent advancements have the potential to significantly expand our understanding of the evolutionary mechanisms underlying plant–insect interactions and contribute to prevent future catastrophic crop damage.

In this study, we used HiFi long reads to assemble genomes for 3 gelechiid moth species (Table 1). Although BUSCO completeness of the *S. atriplicella* genome was relatively low (73.3%), 3,246 of its BUSCO genes could be used to reconstruct a phylogeny with 4 other gelechiid species (Supplementary Fig. S3). However, we note that the robustness of gene family evolution analyses relies heavily on the quality of the genome assembly and the subsequent gene model predictions. The incomplete genome assembly with higher heterozygosity or lower sequence coverage could confound the result by providing missing, incomplete, or duplicated gene prediction. Therefore, we used phylogeny with the 4 more complete genome assemblies and an out-group to detect gene-family evolution with rapid repertoire size changes. We note that the assembly of *P. absoluta*, which has a higher BUSCO duplication rate, likely the result of shallower sequence depth and higher heterozygosity, could possibly overestimate the gene copy number. Although programs such as CAFE were designed to cope with such issues [73], the result of repertoire size changes for species with lower assembly completeness should be interpreted with some caution.

It should also be noted that sequencing interferences were encountered for *S. atriplicella* library samples, which were prepared together with those of *P. absoluta* and *K. lycopersicella* using the same DNA extraction, cleanup, library preparation, and sequencing protocols. We therefore sequenced the amplified DNA library with trial-and-error. According to the BUSCO completeness score, only part of the genome was covered by the HiFi reads despite the high sequence depth (37× and 55× from GenomeScope and ModEst, respectively). This is likely due to replication bias or errors during the amplification process. Based on these experiences, we would recommend optimization of DNA extraction to enable native DNA to be sequenced over the whole genome amplification route we followed with this challenging sample.

Finally, the different methods and assembled sizes resulted in considerable differences in estimated genome sizes. This discrepancy seems to be more prominent in taxa with lower sequence coverage and higher heterozygosity (e.g., *P. absoluta*). We therefore recommend using multiple approaches to better estimate the true genome size and understand the quality of the assembly.

### Genomic adaption of the solanaceous feeding gelechiid moths

Moths use a combination of olfactory (smell) and gustatory (taste or contact) chemoreception to find oviposition sites. Olfactory and gustatory cues are often thought to function in long- and short-range detection of suitable hosts, respectively, but volatile cues at the host surface may also stimulate olfactory sensilla and determine oviposition choice in some species [74]. Indeed, for *P. absoluta*, olfactory cues in the form of tomato leaf volatiles result in oviposition rates indistinguishable from those involving direct contact with the leaf surface [75]. This is not the case for *K. lycopersicella* and *P. operculella*, where contact chemoreception appears to play a more important role in oviposition choice, with surface compounds of host plants shown to stimulate egg-laying in both species [76–78] and those of nonhosts shown to act as deterrents in *P. operculella* [77]. It is notable that our gene family evolution analyses show an increase in rapidly evolving genes associated with host plant sensing, particularly gustatory receptors [79], coincident with a shift to solanaceous feeding in gelechiid moths. This could serve as an indication of selective pressure on host plant association through female oviposition or larval feeding choice. It is likely that caterpillars of leaf-rolling and leaf-mining species are confined to the plant where they hatch [80, 81] and therefore do not search extensively for a new host plant, but the role of contact chemoreception in oviposition choice in gelechiids is better characterized than that of caterpillar host searching.

While an increase in host plant association genes correlates with a shift to feeding on Solanaceae, we did not observe a directional shift in terms of gains/losses. Thus, while we detected gains in gustatory receptor genes in *P. absoluta* and *P. operculella, K. lycopersicella* shows losses in this gene family. While a number of studies have shown a correlation between host range and chemosensory receptor gene repertoire size or specific losses [82–84], the gelechiids studied here appear to have experienced a host shift from one plant family (Amaranthaceae in *S. atriplicella*) to another (Solananceae in *P. absoluta* and its relatives), instead of an expansion or contraction of host range. Thus, we might not expect directional changes in chemosensory receptor repertoire size in instances of host shifts in the same manner that has been observed after expansion or contraction of host range.

For many lepidopteran species, detoxification of plant secondary metabolites is essential in host adaptation [85]. Several genes, including ABC transporters, P450, GMC oxidoreductase, UGT, and insect cuticle protein, play important roles in detoxifying the defending compounds from their host plants [86, 87]. Our gene family evolution analysis reveals that these detoxification genes also rapidly evolve in the focal gelechiid moths, while most expansions are found in the 2 solanaceous feeding species (i.e., *P. absoluta* and *P. operculella*) (Fig. 1). It is noteworthy that *K. lycopersicella*, the sister species of *P. operculella* in our tree, shows only 4 detoxification genes expanding. This result may be explained partially by the different annotation pipelines that were used for these 2 genomes. However, since the gene model of *K. lycopersicella* covers 93.2% of the BUSCO single-copy genes and CAFE was designed controlling such confounding factors from the incomplete or biased annotation, it is fair to conclude that detoxification gene expansion is not a general feature of solanaceous-feeding species [73]. Interestingly, *K. lycopersicella* and *P. absoluta* are found to prefer tomato over potato while *P. operculella* feeds mainly on potato, implying that the feeding and oviposition preferences are not directly related to the evolution of detoxification genes. One other possible explanation is that the rapid expansion of detoxification genes on *P. absoluta* and *P. operculella* has resulted from the frequent exposure to pesticides, as these 2 species are well-known agriculture pests with many pesticide resistances reported [88–90]. Although *K. lycopersicella* is also considered an agricultural pest, the damage it causes is not comparable to the 2 *Phthorimaea* species [7]. Further studies using population genomic approaches to determine the relationship between detoxification–gene evolution and pesticide resistance might provide more evidence supporting or opposing this hypothesis.

One other important mechanism in host adaptation involves digesting nutrients from plant tissue. For many phytophagous insects, coping with host plant protein peptidase inhibitors and efficiently breaking down these complex molecules are an essential first step in digestion [20]. By comparing genomes of 4 gelechiid species, we identified many serine protease genes that are known for its function of host–plant protein digestion. According to the gene evolution results, serine protease genes rapidly expanded in *P. absoluta, P. operculella*, and *P. gossypiella* (Fig. 1). These genes not only digest proteins but also act as species-specific antagonists interfering with the function of host plant peptidase inhibitors [91–93]. The expansion of trypsin and chymotrypsin in 2 global pests on solanaceous crops implies their underlying contributions to important pest species features such as shorter life spans relative to *K. lycopersicella*, a species that has fewer copies of these genes [7, 94–97]. In general, our gene family evolution analysis reveals indirect but important signals of genome evolution underlying the host adaptation in these agricultural pests.

### Rapid evolution of retrotransposable elements and other immune-related genes in gelechiid moths

We found that many of the rapidly evolving genes present in all 4 gelechiid species are genes associated with retrotransposons and reverse transcription. For example, HOGs annotated with *Pao*, a retrotransposable element involved in antiviral mechanism, were found to be rapidly evolving in all 4 gelechiid species (Supplementary Table S3). This element usually contains 5 protein domains where reverse transcriptase (RTase), retrotransposon gag domain, aspartic protease (or aspartic peptidase), and Ribonuclease H superfamily (RNase H) are repeatedly found evolving rapidly [98, 99]. The RTase in this retrovirus-like element reverse-transcribes the invading virus RNA into DNA (stored in retrotransposon sequences or forming a viral circular DNA), and the infection is suppressed by RNase H through cleavage of the DNA–RNA hybrids or by the downstream RNAi pathway [100–104]. Many other significant HOGs found in these gelechiid species may also have similar antiviral mechanisms, including *PiggyBac* transposable element, *Ty3* transposon capsid-like protein, and Transposase, L1 [105, 106] (Supplementary Table S3). However, rapid repertoire size changes of these retrotransposable elements could be the result of the transposon activity instead of gene copy accumulation through recombination.

We also found many rapidly evolving HOGs annotated with immune-related genes such as those involved in the Toll-like receptor (TLC) pathway. These genes (e.g., Toll-like receptor, leucine-rich repeat domain superfamily, and NF-κbitor-interacting Ras-like protein) were found with rapid size changes in all tested gelechiid species. Unlike retrotransposable elements, the TLC pathway targets a wider range of pathogens inclu,ding bacteria, fungi, and viruses. Finally, many other genes that we identified have putative functions in immunity, including serpin superfamily, immunoglobulin, pacifastin domain, and gamma interferon inducible lysosomal thiol reductase. The presence of many rapidly evolving, immune-related genes suggests that managing potential threats from pathogens is also a significant selection pressure. This finding is supported by comparative genomic studies on other moths where viral defending genes (RNase H, RTase, retrotransposon *Pao*, Toll-like receptor, leucine-rich repeat domain) were identified to evolve rapidly [11, 107]. In sum, our gene family evolution approach highlights the importance of host adaptation and immune-related genes in these closely related gelechiid species.

## Availability of Source Code

Project name: Genome Assembling of Gelechiid Moths

Project homepage: https://github.com/yimingweng/genome_assembling_of_gelechiid_moths

Operating system(s): Linux

Programming language: Bash (Unix shell), python

License: MIT

## Supplementary Material

giad103_GIGA-D-23-00053_Original_Submission

giad103_GIGA-D-23-00053_Revision_1

giad103_GIGA-D-23-00053_Revision_2

giad103_GIGA-D-23-00053_Revision_3

giad103_GIGA-D-23-00053_Revision_4

giad103_Response_to_Reviewer_Comments_Original_Submission

giad103_Response_to_Reviewer_Comments_Revision_1

giad103_Response_to_Reviewer_Comments_Revision_2

giad103_Response_to_Reviewer_Comments_Revision_3

giad103_Reviewer_1_Report_Original_Submission Shanlin Liu -- 3/20/2023 Reviewed

giad103_Reviewer_1_Report_Revision_1 Shanlin Liu -- 8/11/2023 Reviewed

giad103_Reviewer_2_Report_Original_Submission Annabel Charlotte Whibley -- 4/10/2023 Reviewed

giad103_Reviewer_2_Report_Revision_1 Annabel Charlotte Whibley -- 8/13/2023 Reviewed

giad103_Reviewer_2_Report_Revision_2 Annabel Charlotte Whibley -- 9/11/2023 Reviewed

giad103_Supplemental_Files

## Data Availability

The data sets supporting the results of this article are available in the NCBI under BioProject PRJNA932016. Supplementary data are also available via the *GigaScience* database GigaDB [108].

## Additional Files

**Supplementary Figure S1**. The k-mer count frequency plots of the three focal species from GenomeScope2, with estimated genome sizes and coverages.

**Supplementary Figure S2**. The blobplot of the three focal gelechiid species.

**Supplementary Figure S3**. The species tree phylogeny based on 4,876 single-copy gene trees. The branch supports are marked on the nodes.

**Supplementary Table S1**. Summary of the predicted gene model from gFACs.

**Supplementary Table S2**. Genome sizes and sequence coverages estimated with backmap (ModEst).

**Supplementary Table S3**. List of the gene family with rapid expansion and contraction.

**Supplementary Table S4**. List of immunity associated gene family.

**Supplementary Table S5**. List of host adaptation associated gene family.

**Supplementary Table S6**. List of enriched GO (gene ontology) term from the rapidly evolving gene families.

## Abbreviations

ABC: ATP-binding cassette transporter; BUSCO: Benchmarking Universal Single-Copy Orthologs; GO: Gene Ontology; HiFi: high fidelity; HOG: hierarchical orthogroup; KEGG: Kyoto Encyclopedia of Genes and Genomes; NCBI: The National Center for Biotechnology Information; PacBio: Pacific Biosciences; RNase H: Ribonuclease H superfamily; RTase: reverse transcriptase; TLC: Toll-like receptor.
