# Supplementary material for: Evolutionary genomics of three agricultural pest moths reveals rapid evolution of host adaptation and immune-related genes
Source: Gigascience. 2024 Jan 2;13:giad103. doi: 10.1093/gigascience/giad103 (PMC10759296; doi:10.1093/gigascience/giad103)
Supplement: giad103_GIGA-D-23-00053_Original_Submission [file giad103_giga-d-23-00053_original_submission.pdf]

GigaScience

Evolutionary genomics of three agricultural pest moths reveals rapid evolution of host adaptation and immune-related genes

--Manuscript Draft--

Manuscript Number:	GIGA-D-23-00053
Full Title:	Evolutionary genomics of three agricultural pest moths reveals rapid evolution of host adaptation and immune-related genes
Article Type:	Research
Funding Information:	USDA APHIS (AP21PPQS&T00C030) — Prof. Akito Y. Kawahara
Abstract:	Understanding the genotype of pest species provides an important baseline for designing integrated pest management (IPM) strategies. Recently developed long-read sequence technologies make it possible to compare genomic features of non-model pest species to disclose the evolutionary path underlying the pest species profiles. While hundreds of published genomes of lepidopteran species (moths and butterflies) are now available, only a few genome assemblies are publicly available from this diverse and notorious agricultural pest family, Gelechiidae. We sequenced and assembled genomes for three gelechiids: Phthorimaea absoluta (tomato leafminer), Keiferia lycopersicella (tomato pinworm), and Scrobipalpa atriplicella (goosefoot groundling moth). We compared these genomes with published genomes of Phthorimaea operculella and Pectinophora gossypiella, and found that the three solanaceous feeding species, Ph. absoluta, K. lycopersicella, and Ph. operculella are clustered together. Gene family evolution analyses with the five species show clear gene family expansions on hostplant associated genes for the three solanaceous feeding species. These genes are involved in host compounding sensing (e.g., gustatory receptors), detoxification (e.g., Cytochrome P450, Glucose-methanol-choline oxidoreductase, Glutathione S-transferase, Insect cuticle proteins, and UDP-glucuronosyl), and digestion (e.g., serine proteases and Aminopeptidase N-type). A gene ontology enrichment analysis of rapid evolving genes also suggests enriched functions in host sensing and immunity. Our results indicate that host plant adaptation and pathogen defense could be important drivers in species diversification among gelechiid moths.
Corresponding Author:	Yi-Ming Weng, Ph.D<br>Florida Museum of Natural History<br>Gainesville, Florida UNITED STATES
Corresponding Author Secondary Information:	
Corresponding Author's Institution:	Florida Museum of Natural History
Corresponding Author's Secondary Institution:	
First Author:	Yi-Ming Weng, Ph.D
First Author Secondary Information:	
Order of Authors:	Yi-Ming Weng, Ph.D
	Shashank R. Pathour, Ph.D
	Keating R. Godfrey, Ph.D
	David Plotkin, Ph.D
	Brandon M. Parker
	Tyler Wist, Ph.D
	Akito Y. Kawahara
Order of Authors Secondary Information:	

Powered by Editorial Manager® and ProduXion Manager® from Aries Systems Corporation

Additional Information:	
**Question**	**Response**
Are you submitting this manuscript to a special series or article collection?	No
**Experimental design and statistics**<br><br>Full details of the experimental design and statistical methods used should be given in the Methods section, as detailed in our Minimum Standards Reporting Checklist. Information essential to interpreting the data presented should be made available in the figure legends.<br><br>Have you included all the information requested in your manuscript?	Yes
**Resources**<br><br>A description of all resources used, including antibodies, cell lines, animals and software tools, with enough information to allow them to be uniquely identified, should be included in the Methods section. Authors are strongly encouraged to cite Research Resource Identifiers (RRIDs) for antibodies, model organisms and tools, where possible.<br><br>Have you included the information requested as detailed in our Minimum Standards Reporting Checklist?	Yes
**Availability of data and materials**<br><br>All datasets and code on which the conclusions of the paper rely must be either included in your submission or deposited in publicly available repositories (where available and ethically appropriate), referencing such data using a unique identifier in the references and in the “Availability of Data and Materials” section of your manuscript.	Yes

Powered by Editorial Manager® and ProduXion Manager® from Aries Systems Corporation

Have you have met the above requirement as detailed in our Minimum Standards Reporting Checklist?	

main manuscript Click here to access/download;Manuscript;Weng_et_al_submission_gigascien

Manuscript for submission to: GigaScience

Title: Evolutionary genomics of three agricultural pest moths reveals rapid evolution of host adaptation and immune-related genes

Yi-Ming Weng[1], Shashank R. Pathour[1,2], R. Keating Godfrey[1], David Plotkin[1], Brandon M. Parker[1], Tyler Wist[3], Akito Y. Kawahara[1]

*Weng and Pathour are co-first authors

[1] McGuire Center for Lepidoptera & Biodiversity, Florida Museum of Natural History, University of Florida, Gainesville, Florida, USA

[2] Division of Entomology, ICAR-Indian Agricultural Research Institute, Pusa, New Delhi 110012, India

[3]Agriculture and Agri-Food Canada, 107 Science Place, Saskatoon, SK, S7N 0X2, Canada

Corresponding author: Akito Y. Kawahara; kawahara@flmnh.ufl.edu

ORCID identifier:

Yi-Ming Weng: 0000-0002-8243-5061

Shashank R. Pathour: 0000-0002-8177-6091

R. Keating Godfrey: 0000-0001-8740-1752

David Plotkin: 0000-0002-2339-655X

Tyler Wist: 0000-0003-3820-2487

Akito Y. Kawahara: 0000-0002-3724-4610

Abstract

Understanding the genotype of pest species provides an important baseline for designing integrated pest management (IPM) strategies. Recently developed long-read sequence technologies make it possible to compare genomic features of non-model pest species to disclose the evolutionary path underlying the pest species profiles. While hundreds of published genomes of lepidopteran species (moths and butterflies) are now available, only a few genome assemblies are publicly available from this diverse and notorious agricultural pest family, Gelechiidae. We sequenced and assembled genomes for three gelechiids: *Phthorimaea absoluta* (tomato leafminer), *Keiferia lycopersicella* (tomato pinworm), and *Scrobipalpa atriplicella* (goosefoot groundling moth). We compared these genomes with published genomes of *Phthorimaea operculella* and *Pectinophora gossypiella*, and found that the three solanaceous feeding species, *Ph. absoluta*, *K. lycopersicella*, and *Ph. operculella* are clustered together. Gene family evolution analyses with the five species show clear gene family expansions on hostplant associated genes for the three solanaceous feeding species. These genes are involved in host compounding sensing (e.g., gustatory receptors), detoxification (e.g., Cytochrome P450, Glucose-methanol-choline oxidoreductase, Glutathione S-transferase, Insect cuticle proteins, and UDP-glucuronosyl), and digestion (e.g., serine proteases and Aminopeptidase N-type). A gene ontology enrichment analysis of rapid evolving genes also suggests enriched functions in host sensing and immunity. Our results indicate that host plant adaptation and pathogen defense could be important drivers in species diversification among gelechiid moths.

Keywords: Gelechiidae, twirler moths, genome assembly, host adaptation, detoxification, immunity, tomato leafminer, tomato pinworm, goosefoot groundling moth

Introduction

Gelechiidae are a diverse family of Lepidoptera comprised of more than 4,700 species [1, 2]. Some species, such as *Phthorimaea absoluta* (tomato leafminer), *Keiferia lycopersicella* (tomato pinworm), and *Phthorimaea operculella* (potato tuber moth), are notorious agricultural pests which could cause more than a billion annual agricultural damage globally [3-10]. The three gelechiids all use of solanaceous plants as larval hosts, but *Phthorimaea absoluta* and *Keiferia lycopersicella* feed primarily on tomato while *Phthorimaea operculella* prefers potato. These species especially the two *Phthorimaea* species are found invading namy non-native regions including Asia, Europe, and Africa. Research on these moths have focused largely on their host preference, identification, and management. Despite their importance as major global pests to agriculture, their genomic framework and the evolutionary process of host plant preference in insect pests is still poorly understood (but see [11]).

Host selection and host use in insects is determined by a series of physiological processes including host plant compound sensing, detoxification, and nutrient digestion. Several genes are thought to be involved in these processes that affect host selection [12]. Genes associated with sensing phytocompounds include olfactory receptor (OR), gustatory receptor (GR), ionotropic receptor (IR), odorant-binding protein (OBP), and chemosensory proteins (CSP). Genes associated with detoxification include cytochrome P450 (P450), ATP-binding cassette transporter (ABC), and glutathione S-transferases (GST), and genes associated with digestion include serine protease (SP) and beta-fructo-furanosidases (BFF) [13-21]. A crucial question in understanding pest evolution is how these genes evolved among pest species and their relatives. Whole genome sequencing of pest species has shown great promise for revealing the evolutionary processes that led to the formation of a pestiferous species. For example, recent studies on the genomic evolution of agricultural pests, with subsequent analyses such as comparative orthologous and gene family evolution, selected region detections, and structural variant analyses, have identified putative genetic bases of their ecological features or pest species profiles [22-25].

Despite the diversity of gelechiid moths, the many studies on the impact of gelechiids to agriculture, and the release of nearly a thousand Lepidoptera genome assemblies in GenBank thus far [26], only five gelechiid genome assemblies are publicly available [11, 27, 28]. Considering its high species diversity and economic importance, more attention and efforts on genomic data accumulation and exploration are required for further understanding the evolution of this moth

family. In this study, we sequenced and assembled the genomes of three gelechiid moth pests, *Keiferia lycopersicella*, *Phthorimaea absoluta*, and *Scrobipalpa atriplicella* to examine their genomic features and how they relate to host preference. Specifically, we investigate how rapidly evolving genes are correlated with host preference and life history.

Materials and Methods

Sample information and sequencing

Three gelechiid moth species (*K. lycopersicella*, *Ph. absoluta*, *S. atriplicella*) were collected from laboratory colonies at University of California, Davis, USA, Bangalore, Karnataka, India, and the Saskatoon Research and Development Centre of Agriculture and Agri-Food, Canada, respectively. Genomic DNA was extracted from the whole moth (larva) using the DNA isolation protocol of the OmniPrep Genomic DNA Extraction Kit (G-Biosciences, St. Louis, MO). For *S. atriplicella*, we encountered sequencing interference for several library samples. Therefore, we amplified genomic DNA with illustra™ GenomiPhi V2 DNA Amplification Kits, Cytiva and the amplified DNA was used to replace the native DNA extracted from the tissue. The genomic and amplified DNA samples were subsequently used to perform fragment size selection and sample purification with the DNeasy PowerClean CleanUp Kit before library preparation. Libraries were sequenced with a single SMRT cell in the Pacbio Sequel IIe system. The DNA clean-up, library construction, and sequencing steps were performed in the Interdisciplinary Center for Biotechnology Research (ICBR) at the University of Florida. The HiFi sequences are deposited in NCBI (BioProject accession number: PRJNA932016; SRA sample accession: SRR23497930, SRR23497929, and SRR23497928).

Genome size and sequence coverage estimations

To verify read quality, we first assessed the HiFi sequence quality using FastQC v 0.11.7 to summarize read profiles [29]. We counted k-mers and calculated the k-mer density distribution for the HiFi reads using K-Mer Counter (KMC) v.3.2.1 with k-mer size of 21 nucleotides. Density distributions were subsequently submitted to GenomeScope v2.0 online tool [30] with default setting for diploid species to estimate the genome size, heterozygosity, sequence coverage and other genomic profiles (**Supplementary Figure S1**). Estimated genome sizes and read coverages

from GENOMESCOPE were used to certify autodetected estimates from HIFIASM assembler (see next section) to ensure the accuracy of autodetected assembling assumptions [31].

Genome assembly, quality assessment, and non-target sequence removal

We used HIFIASM v 0.16.1 to assemble the genome from HiFi reads using default settings, except for reads of *Ph. absoluta*, for which we applied a 2 (-l 2) purging level to keep a greater number of haplotigs for downstream purging. We kept more haplotigs because the sequence coverage for this species was low, and it generated the best assembly evaluated by N50 and BUSCO completeness (based on the lepidoptera_odb10 database) [32, 33]. We also applied the haplotig purging pipeline to remove duplicated haplotigs [34] Haplotig purge details are described in the supplemental methods. Briefly, we mapped HiFi reads (published illumina reads for *Ph. absoluta*) to assemblies to define coverage cutoffs using MINIMAP v. 2.21 (we used BWA for short read mapping for *Ph. absoluta*) [35, 36] Cutoffs were applied to the haplotig purge pipeline for duplicated haplotig removal.

To identify potential non-target sequences in assemblies, we created blobplots using BLOBTOOLS to visualize the distribution of GC content and read coverage for contigs [37]. To determine read coverage, we aligned HiFi reads to the assembly using MINIMAP2 [36]. To assign taxonomy to reads, we used BLASTN to blast contigs against the NCBI nt database with an e-value cutoff of 1e-25. Contigs assigned to non-arthropods with deviating GC content and sequence coverage were determined to be non-target sequences and removed from assemblies (**Supplementary Figure S2**). A BUSCO score was calculated to evaluate the completeness of each assembly (**Table 1**). Genome assemblies of the three species are available through NCBI (BioProject accession number: PRJNA932016).

Gene models and annotations

In the genome annotation pipeline, we first identified repeat regions using REPEATMODELER2 [38]. The genome assemblies were soft-masked with repeats from three lines of evidence including simple and short repeats, the identified repeats from REPEATMODELER2, and the evidence from the lepidopteran repeat database in Repbase using REPEATMASKER with the blast tool RMBLAST [39, 40]. The BRAKER2 gene prediction pipeline was applied to soft-masked genomes [41-47]. For *K. lycopersicella* and *S. atriplicella*, we used arthropod protein sequences

from orthoDB (odb10_arthropoda) in the PROTHINT pipeline to generate hints to train GENEMARK-EP+ [48] and predict gene models alone with the AUGUSTUS. For *Ph. absoluta*, we also included published RNA sequences to train the gene model [49]. Specifically, we ran BRAKER2 pipeline twice (one with protein and one with RNA) and used TSEBRA [50] with default settings to integrate the two models. To further refine models for the three species, we removed genes identified solely by AUGUSTUS *ab initio* prediction without hint supports (e.g., introns, start and stop codons) from the protein database using the python script "selectSupportedSubsets.py" provided by BRAKER2. Final gene models were evaluated using a BUSCO protein model with the lepidoptera_odb10 database. Gene model profiles, including the monoexonic rate and sequence lengths of gene, intron, and exon, were summarized using GFACS v1.0.0 [51] (**Supplemental Table S1**).

For functional annotations, we first annotated gene function by blasting transcript sequences from the BRAKER2 pipeline to the RefSeq non-redundant protein database and Swiss-Prot arthropodan protein database (Reviewed UniPort database) using the blastp function in DIAMOND v2.0.9 [43]. Additionally, we performed default INTERPROSCAN annotation which integrates 14 member databases including PFAM and PANTHER [52]. For gene ontology terms (GO terms) and KEGG pathway annotations, we queried transcript sequences to the PANNZER webserver (Protein annotation with z-score) [53] and KEGG automatic annotation server (KAAS) [54] with bi-directional best hits.

Phylogeny

To explore the evolution of the three gelechiid moths and their genes, we created a phylogeny using two additional published genomes of Gelechiidae: *Phthorimaea operculella* and *Pectinophora gossypiella*. We used the genome of *Hyposmocoma kahamanoa* as an outgroup, as this species belongs to a moth family closely related to Gelechiidae (Cosmopterigidae) [55, 56]. Published genome assembly of *Ph. operculella* (GCA_024500475.1) was downloaded from NCBI GenBank while those of *P. gossypiella* (GCF_024362695.1) and *H. kahamanoa* (GCF_003589595.1) were downloaded from the NCBI Reference Sequence (RefSeq) Database (O'Leary et al., 2016). We performed the same BUSCO approach using the lepidoptera_odb10 database to obtain compatible single-copy amino acid orthologs for these three species [32]. The final data matrix contained 4,876 single copy orthologs that contained at least two ingroup species

and the *H. kahamanoa* outgroup (385 orthologs did not fit these parameters and were removed). Sequences of each ortholog were aligned using default settings in MAFFT version 7.490 [57].

Gene evolution

Phylogenies were constructed using both concatenation and coalescent approaches. For concatenation, we merged all 4,876 gene alignments to create a supermatrix. We assigned a single substitution model (Q.insect+FO+G4 substitution model, the Q matrix estimated for insects) to the entire alignment and built a maximum likelihood tree in IQ-tree v 2.1.3 [58, 59]. Branch supports were calculated using ultrafast bootstrap [60] and SH-aLRT [61]. We also ran coalescent-based analysis in ASTRAL v5.15.5 [62] to estimate the species tree. For the ASTRAL analysis, we ran ModelFinder [63, 64] on each gene to estimate the best substitution model and built 4,876 gene trees, rooted with *H. kahamanoa*.

To investigate gene family evolution, we inferred an ultrametric tree from the concatenated sequence species tree using TREEPL with default settings [65]. For gene family identification, we employed ORTHOFINDER v2.5.2 with amino acid sequences of the annotated gene models from each of the six species [66]. Gene models of the three focal species were predicted from the BRAKER2 pipeline while the other three gene models were directly downloaded from appropriate databases. In ORTHOFINDER, we chose gene families as defined by phylogenetic hierarchical orthogroups (HOGs), an approach which is thought to be more accurate than similarity-based methods [66]. For each gene family, the HOGs gene-counting matrix and ultrametric tree were used to estimate repertoire size changes in CAFE v 5.0.0 [67]. We extracted HOGs under rapid repertoire size expansion and contraction, with the significance level set to 0.01 and branch lengths calculated from the ultrametric tree. For each gene associated with these HOGs, we used the top annotated function (lowest e-value) from INTERPROSCAN to represent the gene function.

For the HOGs with significant rapid expansion and contraction, we assessed their gene functions and GO terms using INTERPROSCAN annotations. To standardize annotations, we reannotated gene functions for the three downloaded gene models (*Phthorimaea operculella*, *Pe. gossypiella*, and *H. kahamanoa*) using default settings in INTERPROSCAN (Jones et al., 2014). For associated GO terms, we performed enrichment analysis using the R package TOPGO 2.40.0 [68] with a significance level of 0.05 for both fisher classic and weight01 algorithms.

Results

Genome assemblies and annotations

To assemble genomes of *K. lycopersicella*, *Ph. absoluta*, and *S. atriplicella*, we used 3.8, 2.2, and 2.9 million PacBio high-fidelity (HiFi) reads corresponding to the estimated read coverage of 25X, 10X, and 37X, respectively. Estimated genome sizes from GENOMESCOPE for *K. lycopersicella*, *Ph. absoluta*, and *S. atriplicella* were 302, 487, and 244 million base pairs (Mb); much smaller than our assemblies. After haplotig removals, considerable reductions in the number of contigs were found while assembled sizes and BUSCO completeness remained nearly consistent, indicating that smaller duplicated contigs were removed. From the assemblies of *K. lycopersicella* and *S. atriplicella*, we identified non-target sequences contributing to a small portion of the assemblies. In *K. lycopersicella*, a 10 kbp-contig was blasted to Streptophyta while in *S. atriplicella*, 15 small contigs (total 380 kbp) were blasted to Proteobacteria. After removing these non-target contigs, 444.65 Mb from 61 contigs, 652.69 Mb from 687 contigs, and 298.57 Mb from 6,960 contigs were found in the assemblies of *K. lycopersicella*, *Ph. absoluta*, and *S. atriplicella*, respectively. BUSCO scores for these assemblies are shown in **Table 1**.

For gene annotation, we first annotated repeats using REPEATMODELER2 [38] and *Ph. absoluta* showed the highest proportion of repeats (54.4%), followed by *K. lycopersicella* (48.22%) and *S. atriplicella* (32.83%). Soft-masked genomes were used to run the BRAKER2 pipeline with protein evidence for *K. lycopersicella* and *S. atriplicella*, resulting in 15,405 and 14,647 genes, respectively [42]. For *Ph. Absoluta*, we used both protein and RNA sequence evidence to predict the gene model. After removing genes without hint support, the gene model with 19,106 genes was used for functional annotation. BUSCO scores for these gene models reflect their assembly features, including the higher duplication rate in *Ph. absoluta* and higher missing rate in *S. atriplicella* (**Table 1**).

Phylogeny and gene family evolution

The maximum likelihood tree, derived from the concatenated supermatrix, shows that *K. lycopersicella* and *Ph. operculella* are most closely related (**Figure 1**). *Phthorimaea absoluta*, another species feeding on solanaceous hosts, is the sister species to *K. lycopersicella* and *Ph. operculella*. *Scrobipalpa atriplicella*, an amaranthaceous feeder, is recovered as the sister taxon to the other three members of subfamily Gelechiinae in the phylogeny. Finally, *Pectinophora*

gossypiella, a member of subfamily Apatetrinae, is the sister taxon to all four Gelechiinae species in the phylogeny, supporting the current taxonomic arrangement [69] (**Figure 1**). We found that the ASTRAL species tree shares the same topology as the ML tree, despite that the branches are not fully supported by the quadripartition supports (**Supplemental Figure S3**).

We identified 15,708 HOGs in the protein sequences of the six species, and 610 HOGs were found to evolve rapidly at least in one branch along the ultrametric tree (**Supplemental Table S2**). Gene family evolution analyses showed a general pattern of rapid expansions at the tips of the tree and rapid contractions along internal branches (**Figure 1**). Specifically, 166 and 92 HOGs were identified in *K. lycopersicella* with rapid repertoire size expansion and contraction, respectively. Among these HOGs, 95 are annotated with gene functions where 4 are putatively involved in host plant adaptation (Otopetrin, Cytochrome P450 superfamily, insect cuticle proteins, and trypsin family serine proteases), 34 involved in immunity (*PiggyBac* transposable elements, Retrotransposon *Pao*-related genes, Serpin superfamilies, and Toll-like receptors), and one associated with pheromone production (Acyltransferase 3 enzyme domain) (**Supplemental Tables S3 and S4**).

For *Ph. operculella*, 376 HOGs (310 expansions and 66 contractions) were identified to evolve rapidly where 244 HOGs (229 expansions and 15 contractions) are annotated with gene functions. From these HOGs, we found that 13 are putatively associated with host plant adaptation including Gustatory receptor, Catalase superfamily, Cytochrome P450 superfamily, Ecdysteroid kinases, Glucose-methanol-choline oxidoreductase, Glutathione S-transferase family, Insect cuticle proteins, and UDP-glucuronosyl. We also found that 51 immune related associated HOGs (Gamma interferon related gene, Pacifastin domain, Serpin superfamily, *PiggyBac* transposable element, Retrotransposon *Pao*-related genes, *Toll*-like receptors, and *Ty3* transposon) and one pheromone signaling related gene (CD36 family). For *Ph. absoluta*, 278 HOGs (236 expansions and 42 contractions) were identified to evolve rapidly, and among them, 120 HOGs (105 expansions and 15 contractions) are annotated with gene functions. These HOGs include 6 host plant adaptation related genes (Gustatory receptor, Cytochrome P450 superfamilies, Epoxide hydrolase, and Serine proteases) and 22 immune related genes (Immunoglobulin, Pacifastin, Retrotransposon *Pao*-related genes, *PiggyBac* transposable element, and *Toll*-like receptor).

For *S. atriplicella*, which is known to feed on amaranthaceous hosts [5], we found 74 rapidly evolving HOGs (20 expansions and 54 contractions) including 25 gene-annotated HOGs

(Nine expansions and 16 contractions). Interestingly, none of the rapidly expanding HOGs are associated with host adaptation but 5 out of 16 rapidly contracting HOGs are associated with detoxification (Catalase superfamily, Cytochrome P450 superfamily, Epoxide hydrolase, Glucose-methanol-choline oxidoreductase, and Insect cuticle protein) and none of them is related to host compound detection or digestion. We also found that 5 HOGs are related to genes associated with Retrotransposon *Pao*. Finally, we found 368 (354 expansions and 14 contractions) rapidly evolving HOGs along the branch of *Pe. gossypiella*, where 193 HOGs have gene annotations (190 expansions and 3 contractions). Among them, 35 HOGs are associated with immunity (Serpin superfamilies, Immunoglobulin-like folds, Pacifastin domain, *PiggyBac* transposable elements, Retrotransposon *Pao*-related genes, and *Toll*-like receptors) and one is related to digestion (Aminopeptidase N-type). However, none are involved in host compound detection or detoxification. A summary of HOGs with putative functions in immunity and host adaptation are listed in **Supplemental Tables S3 & S4**, respectively.

Gene ontology enrichment analyses

From genes that were identified to be rapidly evolving, we found a handful of biological function terms that were shared among species, while others were recovered from only a single species (**Table 2**). For example, sensory perception of taste (GO:0050909), toll-like receptor signaling pathway (GO:0002224), and immune response (GO:0006955) were found in two of the three solanaceous feeding moth species. DNA integration (GO:0015074) was found in three of the five gelechiid species and plasma membrane phospholipid scrambling (GO:0017121) was found in two. All the enriched terms, including those terms passing through fisher classic but not weight01 threshold, are listed in **Supplemental Table S5**.

Discussion

Genome assembly quality and its implications for gene family evolution

Long read sequencing technologies such as Pacific Biosciences (PacBio) and Oxford Nanopore Technologies (ONT) have provided a promising future for *de novo* assemblies of high-quality genomes for non-model species [70, 71]. These recent advancements have the potential to significantly expand our understanding of the evolutionary mechanisms underlying plant–insect interactions and contribute to prevent future catastrophic crop damage.

In this study, we used HiFi long-read to assemble genomes for three gelechiid moth species (**Table 1**). Although BUSCO completeness of the *S. atriplicella* genome was relatively low (73.3%), 3,246 of its BUSCO genes could be used to reconstruct a phylogeny with four other gelechiid species (**Figure 1** and **Supplemental Figure S3**). Phylogenies based on 4,876 single copy genes (including 3,246 sequences from *S. atriplicella*) show consistent results from concatenation and coalescent approaches with identical topology and similar branch lengths, providing a foundation needed to detect gene-family evolution with rapid repertoire size changes. We note, however, that the less complete genome assembly of *S. atriplicella* results in fewer genes being identified through annotation pipelines and could therefore artificially affect contraction and expansion results in the gene family evolution analysis (**Table 1**). Although programs such as CAFE were designed to cope with such issues [72], the result of repertoire size changes for species with lower assembly completeness should be interpreted with some caution.

It should be noted that sequencing interferences were encountered for *S. atriplicella* library samples, which were prepared together with those of *Ph. absoluta* and *K. lycopersicella* using the same DNA extraction, clean-up, library preparation, and sequencing protocols. We therefore sequenced the amplified DNA library the with trial-and-error. According to the BUSCO completeness score, only part of the genome was covered by the HiFi reads despite the high sequence depth (37X). This is likely due to replication bias or errors during the amplification process. Based on this experience, sequencing native DNA directly from the tissue using different DNA extraction strategies is still recommended when similar situations are taking place.

Genomic adaption of the solanaceous feeding gelechiid moths

Moths use a combination of olfactory (smell) and gustatory (taste or contact) chemoreception to find oviposition sites. Olfactory and gustatory cues are often thought to function in long- and short-range detection of suitable hosts, respectively, but volatile cues at the host surface may also stimulate olfactory sensilla and determine oviposition choice in some species [73]. Indeed, for *Ph. absoluta*, olfactory cues in the form of tomato leaf volatiles result in oviposition rates indistinguishable from to those involving direct contact with the leaf surface [74]. This is not the case for *K. lycopersicella* and *P. operculella* where contact chemoreception appears to play a more important role in oviposition choice, with surface compounds of host plants shown to stimulate egg-laying in both species [75-77], and those of non-hosts shown to act as deterrents

in *P. operculella* [76]. It is notable that our gene family evolution analyses show an increase in rapidly evolving genes associated with host plant sensing, particularly gustatory receptors and a sour-sensing channel, otopetrin [78], coincident with a shift to solanaceous feeding in gelechiid moths. This could serve as an indication of selective pressure on host plant association through female oviposition or larval feeding choice. However, it is likely that caterpillars of leaf-rolling and leaf-mining species are confined to the plant where they hatch [79, 80], and therefore do not search extensively for a new host plant. Admittedly, the role of contact chemoreception in oviposition choice is better characterized than that of caterpillar movement and host searching in gelechiids.

While an increase in host plant association genes correlates with a shift to feeding on Solanaceae, we do not observe a directional shift in terms of gains/losses. Thus, while we detected gains in gustatory receptor genes in *Ph. absoluta* and *Ph. operculella*, *K. lycopersicella* shows losses in this gene family. While a number of studies have shown a correlation between host range and chemosensory receptor gene repertoire size or specific losses [81-83], the gelechiids studied here appear to have experienced a host shift from one plant family (Amaranthaceae in *S. atriplicella*) to another (Solananceae in *Ph. absoluta* and its relatives), instead of an expansion or contraction of host range. Thus, we might not expect directional changes in chemosensory receptor repertoire size in instances of host shifts in the same manner that has been observed after expansion or contraction of host range.

For many lepidopteran species, detoxification of plant secondary metabolites is essential in host adaptation [84]. Several genes, including ABC transporters, P450, GMC oxidoreductase, GST, UGT, and insect cuticle protein play important roles in detoxifying the defending compounds from their host plants [85, 86]. Our gene family evolution analysis reveals that these detoxification genes also rapidly evolve in the focal gelechiid moths, while most expansions are found in the two solanaceous feeding species (i.e., *Ph. absoluta* and *Ph. operculella*) (**Figure 1**). Interestingly, *K. lycopersicella*, the sister species of *Ph. operculella* in our tree, shows only one detoxification gene expanding (Epoxide hydrolase). This result may be explained partially by the different annotation pipelines that were used for these two genomes. However, since the gene model of *K. lycopersicella* covers 93.2% of the BUSCO single copy genes and CAFE was designed controlling such confounding factors from the incomplete or biased annotation, it is fair to conclude that detoxification gene expansion is not a general feature of solanaceous-feeding species [72].

Interestingly, *K. lycopersicella* and *Ph. absoluta* are found preferring tomato over potato while *Ph. operculella* feeds mainly on potato, implying that the feeding and oviposition preferences are not directly related to the evolution of detoxification genes. One other possible explanation is that the rapid expansion of detoxification genes on *Ph. absoluta* and *Ph. operculella* is resulted from the frequent exposure to pesticides, as these two species are well-known agriculture pests with many pesticide resistances reported [87-89]. Although *K. lycopersicella* is also considered an agricultural pest, the damage it causes is not comparable to the two *Phthorimaea* species [7]. Further studies using population genomic approaches to determine the relationship between detoxification-gene evolution and pesticide resistance might provide more evidence supporting or opposing this hypothesis.

One other important mechanism in host adaptation involves digesting nutrients from plant tissue. For many phytophagous insects, coping with host plant protein peptidase inhibitors and efficiently breaking down these complex molecules are an essential first step in digestion [20]. By comparing genomes of five gelechiid species, we identified three rapidly evolving genes (Aminopeptidase N-type, trypsin, chymotrypsin) with known functions in plant tissue digestion. The HOG annotated with Aminopeptidase N-type (APN) rapidly expanded in *Pe. gossypiella* and remained relatively consistent in the other four species (**Figure 1**). This gene is found in the larval midgut and aids in protein digestion [90]. The expansion of this gene may be associated with host plant adaptation, but more evidence is suggesting that the accumulation of APN gene copies in *Pe. gossypiella* is associated with its resistance to Cry toxins of *Bacillus thuringiensis* (Bt), the bacterial biocontrol agent being widely applied to control the cotton pests. Interestingly, *Pe. gossypiella* is the only species among the five that is known to have developed significant resistance against Cry toxins as a result of constant exposure to transgenic Bt-crops [91-93]. Our comparative genomic analysis strengthened the previous finding that the rapid evolution of APN gene is playing a vital role in Cry toxin resistance. Trypsin and chymotrypsin are also notable midgut serine proteases whose genes rapidly expanded in *Ph. absoluta* and *Ph. operculella*, respectively (**Figure 1**). These genes not only digest proteins, they also act as species-specific antagonists interfering with the function of host plant peptidase inhibitors [94-96]. The expansion of trypsin and chymotrypsin in two global pests on solanaceous crops implies their underlying contributions to important pest species festures such as shorter life spans relative to *K. lycopersicella*, a species that has fewer copies of these genes [7, 97-100]. In general, our gene

family evolution analysis reveals indirect but important signals of genome evolution underlying the host adaptation in these agricultural pests.

Rapid evolution of retrotransposable elements and other immune related genes in gelechiid moths

We found that many of the rapidly evolving genes present in all five gelechiid species are genes associated with retrotransposons and reverse transcriptome. For example, HOGs annotated with *Pao*, a retrotransposable element involved in antiviral mechanism, were found to be rapidly evolving in all five gelechiid species (**Supplemental Table S2**). This element usually contains five protein domains where reverse transcriptase (RTase), retrotransposon gag domain, aspartic protease (or aspartic peptidase), and Ribonuclease H superfamily (RNase H) are repeatedly found evolving rapidly [101, 102]. The RTase in this retrovirus-like element reverse-transcribes the invading virus RNA into DNA (stored in retrotransposon sequences or forming a viral circular DNA), and the infection is suppressed by RNase H through cleavage of the DNA-RNA hybrids or by the downstream RNAi pathway [103-107]. Many other significant HOGs found in these gelechiid species may also have similar antiviral mechanisms, including *PiggyBac* transposable element, *Ty3* transposon capsid-like protein, and Transposase, L1 [108, 109] (**Supplemental Table S2**). However, rapid repertoire size changes of these retrotransposable elements could be the result of the transposon activity instead of gene copy accumulation through recombination.

We also found many rapidly evolving HOGs annotated with immune related genes such as those involved in the Toll-like receptor (TLC) pathway. These genes (e.g. Toll-like receptor, Leucine-rich repeat domain superfamily, and NF-kappa-B inhibitor-interacting Ras-like protein) were found with rapid size changes in all gelechiid species except *S. atriplicella*. Unlike retrotransposable elements, the TLC pathway targets a wider range of pathogens including bacteria, fungi, and viruses. Finally, many other genes that we identified have putative functions in immunity, including Serpin superfamily, Immunoglobulin, Pacifastin domain, and Gamma interferon inducible lysosomal thiol reductase GILT. The presence of many rapidly evolving, immune-related genes suggests that managing potential threats from pathogens is also a significant selection pressure. This finding is supported by comparative genomic studies on other moths where viral defending genes (RNase H, RTase, retrotransposon *Pao*, Toll-like receptor, Leucine-rich repeat domain) were identified to evolve rapidly [11, 110]. In sum, our gene family evolution

approach highlights the importance of host adaptation and immune-related genes in these closely related gelechiid species.

Availability of source code

1. Genome assembling and analyses for *K. lycopersicella* and *S. atriplicella*: https://github.com/yimingweng/Kely_Scat_genome_project
2. Genome assembling and analyses for *Ph. absoluta:* https://github.com/yimingweng/Tuta_genome_project

Data Availability

The data sets supporting the results of this article are available in the NCBI under BioProject PRJNA932016.

Competing interests

The authors declare that they have no competing interests.

Acknowledgments

We thank USDA APHIS for providing grant funding (grant ID: AP21PPQS&T00C030) to support this study. Thanks also to Dr. N. R. Prasannakumar, ICAR-Indian Institute of Horticultural Research, India for sending samples of *Ph. absoluta*. Analyses were performed on the HiPerGator high-performance computer (University of Florida). PRS expresses his sincere thanks to Dr. Ashok Kumar Singh, Director, Indian Agricultural Research Institute and Indian Council of Agricultural Research, New Delhi, India for necessary permissions and their support.

Author Contributions

AYK, BMR, DP, PRS, and TW, developed the project; PRS and YMW conducted experiments and analyses; RKG and YMW wrote the first draft of the manuscript; AYK, BMR, DP, PRS, and TW, provided comments to improve the manuscript.

References

1. Karsholt O, Mutanen M, Lee S and Kaila L. A molecular analysis of the Gelechiidae (Lepidoptera, Gelechioidea) with an interpretative grouping of its taxa. Systematic Entomology. 2013;38 2:334-48. doi:https://doi.org/10.1111/syen.12006.
2. Van Nieukerken E, Kaila L, Kitching I, Kristensen N, Lees D, Minet J, et al. Animal biodiversity: An outline of higher-level classification and survey of taxonomic richness. Zootaxa. 2011;3148 1:212-21.
3. Biondi A, Guedes RNC, Wan FH and Desneux N. Ecology, Worldwide Spread, and Management of the Invasive South American Tomato Pinworm, Tuta absoluta: Past, Present, and Future. Annu Rev Entomol. 2018;63:239-58. doi:10.1146/annurev-ento-031616-034933.
4. Chang PEC and Metz MA. Classification of *Tuta absoluta* (Meyrick, 1917)(Lepidoptera: Gelechiidae: Gelechiinae: Gnorimoschemini) based on cladistic analysis of morphology. Proceedings of the entomological Society of Washington. 2021;123 1:41-54.
5. Mori BA, Dutcheshen C and Wist TJ. Scrobipalpa atriplicella (Lepidoptera: Gelechiidae), an invasive insect attacking quinoa (Amaranthaceae) in North America. The Canadian Entomologist. 2017;149 4:534-9. doi:10.4039/tce.2017.19.
6. Trivedi TP and Rajagopal D. Distribution, biology, ecology and management of potato tuber moth, Phthorimaea operculella (Zeller) (Lepidoptera: Gelechiidae): A review. Tropical Pest Management. 1992;38 3:279-85. doi:10.1080/09670879209371709.
7. Poe SL. Tomato Pinworm, *Keiferia lycopersicella* (Walshingham). Citeseer; 1999.
8. Rwomushana I, Beale T, Chipabika G, Day R, Gonzalez-Moreno P, Lamontagne-Godwin J, et al. Tomato leafminer (*Tuta absoluta*): impacts and coping strategies for Africa. CABI Working Paper. 2019;12.
9. Venkatramanan S, Wu S, Shi B, Marathe A, Marathe M, Eubank S, et al. Modeling commodity flow in the context of invasive species spread: Study of *Tuta absoluta* in Nepal. Crop Protection. 2020;135:104736.
10. Soares MA and Campos MR. *Phthorimaea absoluta* (tomato leafminer). CABI International; 2022.
11. Zhang M, Cheng X, Lin R, Xie B, Nauen R, Rondon SI, et al. Chromosomal-level genome assembly of potato tuberworm, Phthorimaea operculella: a pest of solanaceous crops. Sci Data. 2022;9 1:748. doi:10.1038/s41597-022-01859-5.
12. Simon J-C, d'Alençon E, Guy E, Jacquin-Joly E, Jaquiéry J, Nouhaud P, et al. Genomics of adaptation to host-plants in herbivorous insects. Briefings in Functional Genomics. 2015;14 6:413-23. doi:10.1093/bfgp/elv015.
13. Ma L, Li ZQ, Bian L, Cai XM, Luo ZX, Zhang YJ, et al. Identification and Comparative Study of Chemosensory Genes Related to Host Selection by Legs Transcriptome Analysis in the Tea Geometrid *Ectropis obliqua*. PLoS One. 2016;11 3:e0149591. doi:10.1371/journal.pone.0149591.
14. Agnihotri AR, Roy AA and Joshi RS. Gustatory receptors in Lepidoptera: chemosensation and beyond. Insect Molecular Biology. 2016;25 5:519-29. doi:10.1111/imb.12246.
15. Barve PR, Tellis MB, Barvkar VT, Joshi RS, Giri AP and Kotkar HM. Functional Diversity of the Lepidopteran ATP-Binding Cassette Transporters. Journal of Molecular Evolution. 2022;90 3-4:258-70. doi:10.1007/s00239-022-10056-2.
16. Calla B, Noble K, Johnson RM, Walden KKO, Schuler MA, Robertson HM, et al. Cytochrome P450 diversification and hostplant utilization patterns in specialist and generalist moths: Birth, death and adaptation. Molecular Ecology. 2017;26 21:6021-35. doi:https://doi.org/10.1111/mec.14348.
17. Cui WC, Wang B, Guo MB, Liu Y, Jacquin-Joly E, Yan SC, et al. A receptor-neuron correlate for the detection of attractive plant volatiles in *Helicoverpa assulta* (Lepidoptera: Noctuidae). Insect Biochemistry and Molecular Biology. 2018;97:31-9. doi:10.1016/j.ibmb.2018.04.006.

18. Koirala B K S, Moural T and Zhu F. Functional and Structural Diversity of Insect Glutathione S-transferases in Xenobiotic Adaptation. International Journal of Biological Sciences. 2022;18 15:5713-23. doi:10.7150/ijbs.77141.
19. Liu NY, Xu W, Dong SL, Zhu JY, Xu YX and Anderson A. Genome-wide analysis of ionotropic receptor gene repertoire in Lepidoptera with an emphasis on its functions of *Helicoverpa armigera*. Insect Biochem Mol Biol. 2018;99:37-53. doi:10.1016/j.ibmb.2018.05.005.
20. Srinivasan A, Giri AP and Gupta VS. Structural and functional diversities in lepidopteran serine proteases. Cellular & Molecular Biology Letters. 2006;11 1:132-54. doi:10.2478/s11658-006-0012-8.
21. Sun L, Mao TF, Zhang YX, Wu JJ, Bai JH, Zhang YN, et al. Characterization of candidate odorant-binding proteins and chemosensory proteins in the tea geometrid *Ectropis obliqua* Prout (Lepidoptera: Geometridae). Archives of Insect Biochemistry and Physiology. 2017;94 4 doi:10.1002/arch.21383.
22. Cohen ZP, Brevik K, Chen YH, Hawthorne DJ, Weibel BD and Schoville SD. Elevated rates of positive selection drive the evolution of pestiferousness in the Colorado potato beetle (*Leptinotarsa decemlineata*, Say). Molecular Ecology. 2021;30 1:237-54. doi:10.1111/mec.15703.
23. Hazzouri KM, Sudalaimuthuasari N, Kundu B, Nelson D, Al-Deeb MA, Le Mansour A, et al. The genome of pest Rhynchophorus ferrugineus reveals gene families important at the plant-beetle interface. Communications Biology. 2020;3 1:323. doi:10.1038/s42003-020-1060-8.
24. Li M, Yang X, Fan F, Ge Y, Hong D, Wang Z, et al. De novo genome assembly of *Bradysia cellarum* (Diptera: Sciaridae), a notorious pest in traditional special vegetables in China. Insect Molecular Biology. 2022;31 4:508-18. doi:10.1111/imb.12776.
25. Powell D, Groβe-Wilde E, Krokene P, Roy A, Chakraborty A, Löfstedt C, et al. A highly-contiguous genome assembly of the Eurasian spruce bark beetle, *Ips typographus*, provides insight into a major forest pest. Communications Biology. 2021;4 1:1059. doi:10.1038/s42003-021-02602-3.
26. Benson DA, Karsch-Mizrachi I, Lipman DJ, Ostell J, Rapp BA and Wheeler DL. GenBank. Nucleic Acids Res. 2000;28 1:15-8. doi:10.1093/nar/28.1.15.
27. Stahlke AR, Chang J, Chudalayandi S, Heu CC, Geib SM, Scheffler BE, et al. Chromosome-scale genome assembly of the pink bollworm, *Pectinophora gossypiella*, a global pest of cotton. bioRxiv. 2022.
28. Tabuloc CA, Lewald KM, Conner WR, Lee Y, Lee EK, Cain AB, et al. Sequencing of Tuta absoluta genome to develop SNP genotyping assays for species identification. Journal of Pest Science. 2019;92 4:1397-407. doi:10.1007/s10340-019-01116-6.
29. Andrews S. FastQC: a quality control tool for high throughput sequence data. Babraham Bioinformatics, Babraham Institute, Cambridge, United Kingdom, 2010.
30. Ranallo-Benavidez TR, Jaron KS and Schatz MC. GenomeScope 2.0 and Smudgeplot for reference-free profiling of polyploid genomes. Nature Communications. 2020;11 1:1432. doi:10.1038/s41467-020-14998-3.
31. Cheng H, Concepcion GT, Feng X, Zhang H and Li H. Haplotype-resolved de novo assembly using phased assembly graphs with hifiasm. Nature Methods. 2021;18 2:170-5. doi:10.1038/s41592-020-01056-5.
32. Seppey M, Manni M and Zdobnov EM. BUSCO: Assessing Genome Assembly and Annotation Completeness. Methods in Molecular Biology. 2019;1962:227-45. doi:10.1007/978-1-4939-9173-0_14.
33. Simão FA, Waterhouse RM, Ioannidis P, Kriventseva EV and Zdobnov EM. BUSCO: assessing genome assembly and annotation completeness with single-copy orthologs. Bioinformatics. 2015;31 19:3210-2. doi:10.1093/bioinformatics/btv351.

34. Roach MJ, Schmidt SA and Borneman AR. Purge Haplotigs: allelic contig reassignment for third-gen diploid genome assemblies. BMC Bioinformatics. 2018;19 1:460. doi:10.1186/s12859-018-2485-7.
35. Li H. Aligning sequence reads, clone sequences and assembly contigs with BWA-MEM. arXiv preprint arXiv:13033997. 2013.
36. Li H. Minimap2: pairwise alignment for nucleotide sequences. Bioinformatics. 2018;34 18:3094-100. doi:10.1093/bioinformatics/bty191.
37. Challis R, Richards E, Rajan J, Cochrane G and Blaxter M. BlobToolKit – Interactive Quality Assessment of Genome Assemblies. G3 Genes|Genomes|Genetics. 2020;10 4:1361-74. doi:10.1534/g3.119.400908.
38. Flynn JM, Hubley R, Goubert C, Rosen J, Clark AG, Feschotte C, et al. RepeatModeler2 for automated genomic discovery of transposable element families. Proceedings of the National Academy of Sciences. 2020;117 17:9451-7. doi:10.1073/pnas.1921046117.
39. Smit A, Hubley R and Green P. RepeatMasker Open-4.0 [http://www. repeatmasker. org] Accessed September, 2020.: Accessed, 2015.
40. Jurka J. Repeats in genomic DNA: mining and meaning. Current Opinion in Structural Biology. 1998;8 3:333-7. doi:10.1016/s0959-440x(98)80067-5.
41. Barnett DW, Garrison EK, Quinlan AR, Strömberg MP and Marth GT. BamTools: a C++ API and toolkit for analyzing and managing BAM files. Bioinformatics. 2011;27 12:1691-2. doi:10.1093/bioinformatics/btr174.
42. Brůna T, Hoff KJ, Lomsadze A, Stanke M and Borodovsky M. BRAKER2: automatic eukaryotic genome annotation with GeneMark-EP+ and AUGUSTUS supported by a protein database. NAR Genom Bioinform. 2021;3 1:lqaa108. doi:10.1093/nargab/lqaa108.
43. Buchfink B, Xie C and Huson DH. Fast and sensitive protein alignment using DIAMOND. Nature Methods. 2015;12 1:59-60. doi:10.1038/nmeth.3176.
44. Hoff KJ, Lomsadze A, Borodovsky M and Stanke M. Whole-Genome Annotation with BRAKER. Methods in Molecular Biology. 2019;1962:65-95. doi:10.1007/978-1-4939-9173-0_5.
45. Lomsadze A, Burns PD and Borodovsky M. Integration of mapped RNA-Seq reads into automatic training of eukaryotic gene finding algorithm. Nucleic acids research. 2014;42 15:e119-e.
46. Stanke M, Schöffmann O, Morgenstern B and Waack S. Gene prediction in eukaryotes with a generalized hidden Markov model that uses hints from external sources. BMC Bioinformatics. 2006;7:62. doi:10.1186/1471-2105-7-62.
47. Li H, Handsaker B, Wysoker A, Fennell T, Ruan J, Homer N, et al. The sequence alignment/map format and SAMtools. Bioinformatics. 2009;25 16:2078-9.
48. Brůna T, Lomsadze A and Borodovsky M. GeneMark-EP+: eukaryotic gene prediction with self-training in the space of genes and proteins. NAR Genom Bioinform. 2020;2 2:lqaa026. doi:10.1093/nargab/lqaa026.
49. Camargo RA, Barbosa GO, Possignolo IP, Peres LE, Lam E, Lima JE, et al. RNA interference as a gene silencing tool to control *Tuta absoluta* in tomato (*Solanum lycopersicum*). PeerJ. 2016;4:e2673. doi:10.7717/peerj.2673.
50. Gabriel L, Hoff KJ, Brůna T, Borodovsky M and Stanke M. TSEBRA: transcript selector for BRAKER. BMC Bioinformatics. 2021;22 1:566. doi:10.1186/s12859-021-04482-0.
51. Caballero M and Wegrzyn J. gFACs: Gene Filtering, Analysis, and Conversion to Unify Genome Annotations Across Alignment and Gene Prediction Frameworks. Genomics, Proteomics & Bioinformatics. 2019;17 3:305-10. doi:https://doi.org/10.1016/j.gpb.2019.04.002.
52. Jones P, Binns D, Chang H-Y, Fraser M, Li W, McAnulla C, et al. InterProScan 5: genome-scale protein function classification. Bioinformatics. 2014;30 9:1236-40. doi:10.1093/bioinformatics/btu031.

53. Koskinen P, Törönen P, Nokso-Koivisto J and Holm L. PANNZER: high-throughput functional annotation of uncharacterized proteins in an error-prone environment. Bioinformatics. 2015;31 10:1544-52. doi:10.1093/bioinformatics/btu851.
54. Moriya Y, Itoh M, Okuda S, Yoshizawa AC and Kanehisa M. KAAS: an automatic genome annotation and pathway reconstruction server. Nucleic Acids Res. 2007;35 Web Server issue:W182-5. doi:10.1093/nar/gkm321.
55. Sohn JC, Regier JC, Mitter C, Adamski D, Landry JF, Heikkilae M, et al. Phylogeny and feeding trait evolution of the mega- diverse Gelechioidea (Lepidoptera: Obtectomera): new insight from 19 nuclear genes. Systematic Entomology. 2016;41 1:112-32.
56. Kawahara AY, Plotkin D, Espeland M, Meusemann K, Toussaint EFA, Donath A, et al. Phylogenomics reveals the evolutionary timing and pattern of butterflies and moths. Proceedings of the National Academy of Sciences. 2019;116 45:22657-63. doi:10.1073/pnas.1907847116.
57. Katoh K and Standley DM. MAFFT multiple sequence alignment software version 7: improvements in performance and usability. Molecular biology and evolution. 2013;30 4:772-80.
58. Minh BQ, Dang CC, Vinh LS and Lanfear R. QMaker: Fast and Accurate Method to Estimate Empirical Models of Protein Evolution. Systematic Biology. 2021;70 5:1046-60. doi:10.1093/sysbio/syab010.
59. Misof B, Liu S, Meusemann K, Peters RS, Donath A, Mayer C, et al. Phylogenomics resolves the timing and pattern of insect evolution. Science. 2014;346 6210:763-7. doi:10.1126/science.1257570.
60. Minh BQ, Nguyen MA and von Haeseler A. Ultrafast approximation for phylogenetic bootstrap. Molecular Biology and Evolution. 2013;30 5:1188-95. doi:10.1093/molbev/mst024.
61. Guindon S, Dufayard JF, Lefort V, Anisimova M, Hordijk W and Gascuel O. New algorithms and methods to estimate maximum-likelihood phylogenies: assessing the performance of PhyML 3.0. Systematic Biology. 2010;59 3:307-21. doi:10.1093/sysbio/syq010.
62. Zhang C, Rabiee M, Sayyari E and Mirarab S. ASTRAL-III: polynomial time species tree reconstruction from partially resolved gene trees. BMC bioinformatics. 2018;19 6:153.
63. Kalyaanamoorthy S, Minh BQ, Wong TKF, von Haeseler A and Jermiin LS. ModelFinder: fast model selection for accurate phylogenetic estimates. Nature Methods. 2017;14 6:587-9. doi:10.1038/nmeth.4285.
64. Nguyen L-T, Schmidt HA, Von Haeseler A and Minh BQ. IQ-TREE: a fast and effective stochastic algorithm for estimating maximum-likelihood phylogenies. Molecular biology and evolution. 2015;32 1:268-74.
65. Smith SA and O'Meara BC. treePL: divergence time estimation using penalized likelihood for large phylogenies. Bioinformatics. 2012;28 20:2689-90. doi:10.1093/bioinformatics/bts492.
66. Emms DM and Kelly S. OrthoFinder: phylogenetic orthology inference for comparative genomics. Genome Biology. 2019;20 1:238. doi:10.1186/s13059-019-1832-y.
67. Ganote C, Mendes F, Henschel R, Hahn M and Fulton B. Introducing CAFE: Computational Analysis of (gene) Family Evolution. Bioinformatics. 2018;22 10:1269–71.
68. Alexa A and Rahnenführer J. Gene set enrichment analysis with topGO. Bioconductor Improv. 2009;27:1-26.
69. Lee G-E, Han T, Park H, Qi M and Li H. A phylogeny of the subfamily Thiotrichinae (Lepidoptera: Gelechiidae) with a revision of the generic classification based on molecular and morphological analyses. Systematic Entomology. 2021;46 2:357-79. doi:https://doi.org/10.1111/syen.12466.
70. Gavrielatos M, Kyriakidis K, Spandidos DA and Michalopoulos I. Benchmarking of next and third generation sequencing technologies and their associated algorithms for *de novo* genome assembly. Molecular Medicine Reports. 2021;23 4 doi:10.3892/mmr.2021.11890.
71. Lang D, Zhang S, Ren P, Liang F, Sun Z, Meng G, et al. Comparison of the two up-to-date sequencing technologies for genome assembly: HiFi reads of Pacific Biosciences Sequel II

system and ultralong reads of Oxford Nanopore. GigaScience. 2020;9 12 doi:10.1093/gigascience/giaa123.

72. Han MV, Thomas GW, Lugo-Martinez J and Hahn MW. Estimating gene gain and loss rates in the presence of error in genome assembly and annotation using CAFE 3. Mol Biol Evol. 2013;30 8:1987-97. doi:10.1093/molbev/mst100.
73. Li R-T, Huang L-Q, Dong J-F and Wang C-Z. A moth odorant receptor highly expressed in the ovipositor is involved in detecting host-plant volatiles. eLife. 2020;9:e53706. doi:10.7554/eLife.53706.
74. Proffit M, Birgersson G, Bengtsson M, Reis R, Witzgall P and Lima E. Attraction and oviposition of *Tuta absoluta* females in response to tomato leaf volatiles. Journal of Chemical Ecology. 2011;37 6:565-74. doi:10.1007/s10886-011-9961-0.
75. Burton RL and Schuster DJ. Oviposition Stimulant for Tomato Pinworms1 from Surfaces of Tomato Plants2. Annals of the Entomological Society of America. 1981;74 5:512-5. doi:10.1093/aesa/74.5.512.
76. Fenemore P. Oviposition of potato tuber moth, Phthorimaea operculella Zell.(Lepidoptera: Gelechiidae); identification of host-plant factors influencing oviposition response. New Zealand Journal of Zoology. 1980;7 3:435-9.
77. Meisner J, Ascher KRS and Lavie D. Factors influencing the attraction to oviposition of the potato tuber moth, Gnorimoschema operculella Zell.1. Zeitschrift für Angewandte Entomologie. 1974;77 1-4:179-89. doi:https://doi.org/10.1111/j.1439-0418.1974.tb03245.x.
78. Tu YH, Cooper AJ, Teng B, Chang RB, Artiga DJ, Turner HN, et al. An evolutionarily conserved gene family encodes proton-selective ion channels. Science. 2018;359 6379:1047-50. doi:10.1126/science.aao3264.
79. Visalakshmi J and Johnson J. Studies on a leaf feeding caterpillar of sweet potato, *Brachmia gonvolvuli* Wlsm.(Gelechiidae: Lepidoptera). Agricultural research journal of Kerala. 1968;6 1.
80. Schuster D. Development of Tomato Pinworm (Lepodoptera: Gelechiidae) on Foliage of Selected Plant Species. The Florida Entomologist. 1989;72 1:216-9.
81. Andersson MN, Keeling CI and Mitchell RF. Genomic content of chemosensory genes correlates with host range in wood-boring beetles (Dendroctonus ponderosae, Agrilus planipennis, and Anoplophora glabripennis). BMC Genomics. 2019;20 1:690. doi:10.1186/s12864-019-6054-x.
82. Goldman-Huertas B, Mitchell RF, Lapoint RT, Faucher CP, Hildebrand JG and Whiteman NK. Evolution of herbivory in Drosophilidae linked to loss of behaviors, antennal responses, odorant receptors, and ancestral diet. Proceedings of the National Academy of Sciences. 2015;112 10:3026-31. doi:10.1073/pnas.1424656112.
83. Xu W, Papanicolaou A, Zhang H-J and Anderson A. Expansion of a bitter taste receptor family in a polyphagous insect herbivore. Scientific Reports. 2016;6 1:1-10.
84. Groen SC and Whiteman NK. Ecology and Evolution of Secondary Compound Detoxification Systems in Caterpillars. Caterpillars in the Middle: Tritrophic Interactions in a Changing World. Springer; 2022. p. 115-63.
85. Breeschoten T, van der Linden CFH, Ros VID, Schranz ME and Simon S. Expanding the Menu: Are Polyphagy and Gene Family Expansions Linked across Lepidoptera? Genome Biology and Evolution. 2022;14 1 doi:10.1093/gbe/evab283.
86. Heidel-Fischer HM and Vogel H. Molecular mechanisms of insect adaptation to plant secondary compounds. Current Opinion in Insect Science. 2015;8:8-14. doi:10.1016/j.cois.2015.02.004.
87. El-Kady H. Insecticide resistance in potato tuber moth Phthorimaea operculella Zeller in Egypt. The Journal of American Science. 2011;7 10:263-6.
88. Langa TP, Dantas KC, Pereira DL, de Oliveira M, Ribeiro LM and Siqueira HA. Basis and monitoring of methoxyfenozide resistance in the South American tomato pinworm *Tuta absoluta*. Journal of Pest Science. 2022;95 1:351-64.
89. Zibaee I. The expression profile of detoxifying enzyme of tomato leaf miner, *Tuta absoluta* Meyrik (Lepidoptera: Gelechiidae) to chlorpyrifos. Arthropods. 2016;5 2:77.

90. Terra WR and Ferreira C. Insect digestive enzymes: properties, compartmentalization and function. Comparative Biochemistry and Physiology Part B: Comparative Biochemistry. 1994;109 1:1-62.
91. Jalapathi SK, Jayaraj J, Shanthi M, Theradimani M, Venkatasamy B, Irulandi S, et al. Potential of Cry1Ac from Bacillus thuringiensis against the tomato pinworm, Tuta absoluta (Meyrick) (Gelechiidae: Lepidoptera). Egyptian Journal of Biological Pest Control. 2020;30 1:81. doi:10.1186/s41938-020-00283-4.
92. Mohammed A, Douches DS, Pett W, Grafius E, Coombs J, Liswidowati, et al. Evaluation of potato tuber moth (Lepidoptera: Gelechiidae) resistance in tubers of Bt-cry5 transgenic potato lines. Journal of Economic Entomology. 2000;93 2:472-6. doi:10.1603/0022-0493-93.2.472.
93. Seal DR and Leibee GL. Toxicity of Bacillus thuringiensis CRY1-type insecticidal toxin to geographically distant populations of tomato pinworm. The Florida Entomologist. 2003;86 2:222-4.
94. Dias RO, Via A, Brandão MM, Tramontano A and Silva-Filho MC. Digestive peptidase evolution in holometabolous insects led to a divergent group of enzymes in Lepidoptera. Insect Biochemistry and Molecular Biology. 2015;58:1-11. doi:10.1016/j.ibmb.2014.12.009.
95. Kumar R, Bhardwaj U, Kumar P and Mazumdar-Leighton S. Midgut serine proteases and alternative host plant utilization in *Pieris brassicae* L. Frontiers in Physiology. 2015;6:95. doi:10.3389/fphys.2015.00095.
96. Souza TP, Dias RO, Castelhano EC, Brandão MM, Moura DS and Silva-Filho MC. Comparative analysis of expression profiling of the trypsin and chymotrypsin genes from Lepidoptera species with different levels of sensitivity to soybean peptidase inhibitors. Comparative Biochemistry and Physiology Part B. 2016;196-197:67-73. doi:10.1016/j.cbpb.2016.02.007.
97. Bell HA, Fitches EC, Down RE, Ford L, Marris GC, Edwards JP, et al. Effect of dietary cowpea trypsin inhibitor (CpTI) on the growth and development of the tomato moth *Lacanobia oleracea* (Lepidoptera: Noctuidae) and on the success of the gregarious ectoparasitoid *Eulophus pennicornis* (Hymenoptera: Eulophidae). Pest Management Science. 2001;57 1:57-65. doi:10.1002/1526-4998(200101)57:1<57::AID-PS273>3.0.CO;2-4.
98. Gharekhani GH and Salek-Ebrahimi H. Life table parameters of the tomato leaf miner *Tuta absoluta* (Lepidoptera: Gelechiidae) on different tomato cultivars. Journal of Applied Entomology. 2014;107 5:1765-70. doi:10.1603/EC14059.
99. Golizadeh A, Esmaeili N, Razmjou J and Rafiee-Dastjerdi H. Comparative life tables of the potato tuberworm, *Phthorimaea operculella*, on leaves and tubers of different potato cultivars. Journal of Insect Science. 2014;14:42. doi:10.1093/jis/14.1.42.
100. Hemmati SA, Takalloo Z, Taghdir M, Mehrabadi M, Balalaei S, Moharramipour S, et al. The trypsin inhibitor pro-peptide induces toxic effects in Indianmeal moth, *Plodia interpunctella*. Pesticide Biochemistry and Physiology. 2021;171:104730. doi:10.1016/j.pestbp.2020.104730.
101. Dezordi FZ, Vasconcelos CRDS, Rezende AM and Wallau GL. In and Outs of Chuviridae Endogenous Viral Elements: Origin of a Potentially New Retrovirus and Signature of Ancient and Ongoing Arms Race in Mosquito Genomes. Front Genet. 2020;11:542437. doi:10.3389/fgene.2020.542437.
102. Xiong Y, Burke WD and Eickbush TH. Pao, a highly divergent retrotransposable element from *Bombyx mori* containing long terminal repeats with tandem copies of the putative R region. Nucleic Acids Research. 1993;21 9:2117-23. doi:10.1093/nar/21.9.2117.
103. Goic B, Vodovar N, Mondotte JA, Monot C, Frangeul L, Blanc H, et al. RNA-mediated interference and reverse transcription control the persistence of RNA viruses in the insect model *Drosophila*. Nature Immunology. 2013;14 4:396-403. doi:10.1038/ni.2542.
104. Moelling K, Broecker F, Russo G and Sunagawa S. RNase H As Gene Modifier, Driver of Evolution and Antiviral Defense. Frontiers in Microbiology. 2017;8:1745. doi:10.3389/fmicb.2017.01745.

105. Tassetto M, Kunitomi M, Whitfield ZJ, Dolan PT, Sánchez-Vargas I, Garcia-Knight M, et al. Control of RNA viruses in mosquito cells through the acquisition of vDNA and endogenous viral elements. Elife. 2019;8 doi:10.7554/eLife.41244.
106. Wu J, Wu C, Xing F, Cao L, Zeng W, Guo L, et al. Endogenous reverse transcriptase and RNase H-mediated antiviral mechanism in embryonic stem cells. Cell Research. 2021;31 9:998-1010. doi:10.1038/s41422-021-00524-7.
107. Zhu M, Pan J, Tong X, Qiu Q, Zhang X, Zhang Y, et al. BmCPV-Derived Circular DNA vcDNA-S7 Mediated by *Bombyx mori* Reverse Transcriptase (RT) Regulates BmCPV Infection. Frontiers in Immunology. 2022;13:861007. doi:10.3389/fimmu.2022.861007.
108. Dodonova SO, Prinz S, Bilanchone V, Sandmeyer S and Briggs JAG. Structure of the Ty3/Gypsy retrotransposon capsid and the evolution of retroviruses. Proceedings of the National Academy of Sciences. 2019;116 20:10048-57. doi:10.1073/pnas.1900931116.
109. Rohrmann GF. Baculoviruses, retroviruses, DNA transposons (piggyBac), and insect cells. Baculovirus Molecular Biology [Internet] 4th edition. National Center for Biotechnology Information (US); 2019.
110. Yan B, Ou H, Wei L, Wang X, Yu X, Liu J, et al. A chromosome-level genome assembly of *Ephestia elutella* (Hübner, 1796)(Lepidoptera: Pyralidae). Genome Biology and Evolution. 2021;13 8:evab114.

Tables

Table 1. Assembly statistics of the three newly sequenced gelechiid moth species, compared to statistics of the published *Phthorimaea absoluta* v1 assembly. BUSCO results from the *Phthorimaea absoluta* v1 assembly have been re-analyzed using BUSCO v5.

	***Phthorimaea absoluta* v1**	***Phthorimaea absoluta* v2**	***Keiferia lycopersicella***	***Scrobipalpa atriplicella***
Number of contigs	51,398	688	61	7,107
Total length	906,539,853	652,703,157	443,647,192	301,531,120
GC content	38.11%	38.45%	38.86%	36.86%
Contig N50	97,121	1,614,219	14,556,016	51,574
Contig L50	1,787	115	11	1,807
genome BUSCO complete (C)	C:90.3%[S:67.1%,D:23.2%]	96.2%[S:82.5%,D:13.7%]	96.6%[S:95.5%,D:1.1%]	C:73.3%[S:69.7%,D:3.6%]
genome BUSCO fragmented (F)	3.4%	0.5%	0.7%	2.8%
genome BUSCO missing (M)	6.3%	3.3%	2.7%	23.9%
Repeat percentage	-	54.4%	48.22%	32.83%
number of protein coding genes	-	19,106	15,405	14,647
gene model BUSCO (C)	-	93.2%[S:75.9%,D:17.3%]	93.2%[S:91.7%,D:1.5%]	70.2%[S:60.6%,D:9.6%]
gene model BUSCO (F)	-	1.3%	0.7%	3.4%
gene model BUSCO (M)	-	5.5%	6.1%	26.4%
number of monoexonic genes	-	2,568	2,040	1,600
Reference	Tabuloc et al., 2019	this study	this study	this study

Table 2. Enriched GO terms from the rapidly evolving genes of the five gelechiid species in this study.

**Species**	**GO term ID**	**Biological Function**	P_{weight}	$P_{classic}$
K. lycopersicella	GO:0002224	toll-like receptor signaling pathway	8.50E-03	8.52E-03
	GO:0006955	immune response	2.54E-02	2.54E-02
	GO:0015074	DNA integration	8.50E-03	8.52E-03
	GO:0016573	histone acetylation	8.50E-03	8.52E-03
	GO:0051560	mitochondrial calcium ion homeostasis	1.70E-02	1.70E-02
Ph. operculella	GO:0006508	proteolysis	1.30E-03	1.32E-03
	GO:0006979	response to oxidative stress	1.40E-03	1.42E-03
	GO:0015074	DNA integration	6.30E-07	6.33E-07
	GO:0050909	sensory perception of taste	6.20E-11	6.23E-11
Ph. absoluta	GO:0002224	toll-like receptor signaling pathway	2.19E-02	2.19E-02
	GO:0006313	transposition, DNA-mediated	2.73E-03	2.73E-03
	GO:0006821	chloride transport	4.33E-02	4.33E-02
	GO:0006955	immune response	4.33E-02	4.33E-02
	GO:0007275	multicellular organism development	1.82E-10	1.82E-10
	GO:0007304	chorion-containing eggshell formation	4.42E-15	4.42E-15
	GO:0010923	negative regulation of phosphatase activity	5.18E-10	5.18E-10
	GO:0017121	plasma membrane phospholipid scrambling	4.68E-04	4.68E-04
	GO:0050909	sensory perception of taste	2.18E-02	2.18E-02
	GO:0090522	vesicle tethering involved in exocytosis	4.33E-02	4.33E-02
S. atriplicella	GO:0000413	protein peptidyl-prolyl isomerization	1.10E-04	1.08E-04
	GO:0006355	regulation of transcription, DNA-templated	2.02E-03	2.02E-03
Pe. gossypiella	GO:0006334	nucleosome assembly	9.37E-27	9.37E-27
	GO:0006468	protein phosphorylation	4.16E-07	4.16E-07
	GO:0006486	protein glycosylation	2.28E-02	2.28E-02
	GO:0006631	fatty acid metabolic process	1.11E-05	1.11E-05
	GO:0006811	ion transport	1.36E-06	4.64E-04
	GO:0006915	apoptotic process	1.18E-09	1.18E-09
	GO:0007411	axon guidance	1.08E-04	1.08E-04
	GO:0015074	DNA integration	2.89E-15	2.89E-15
	GO:0017121	plasma membrane phospholipid scrambling	4.70E-08	4.70E-08
	GO:0070588	calcium ion transmembrane transport	1.27E-03	1.27E-03

Figure legends

Figure 1. (A) Maximum likelihood tree of five gelechiid species from a concatenated supermatrix analysis of 4,876 single-copy genes, presented alongside a color-coded number of rapidly evolving gene families (red: expanding, blue: contracting). The tree is rooted with *Hyposmocoma kahamanoa* (Gelechioidea: Cosmopterigidae). Nodes are labelled with branch supports (ultrafast bootstrap/SH-aLRT). (B) The list of rapidly evolving gene families that are associated with host plants includes two host compound-sensing gene families, 16 detoxification genes, and three digestion-related genes. Numbers in color-coded cells represent repertoire size change in corresponding branches on the tree in (A), and gene-family functions are shown at the top of columns. The significant repertoire size changes are marked with outside borders on the cell. References supporting categorizations of gene function are provided in **Supplemental Table S4**.

Click here to access/download;Figure;Figure1.tif

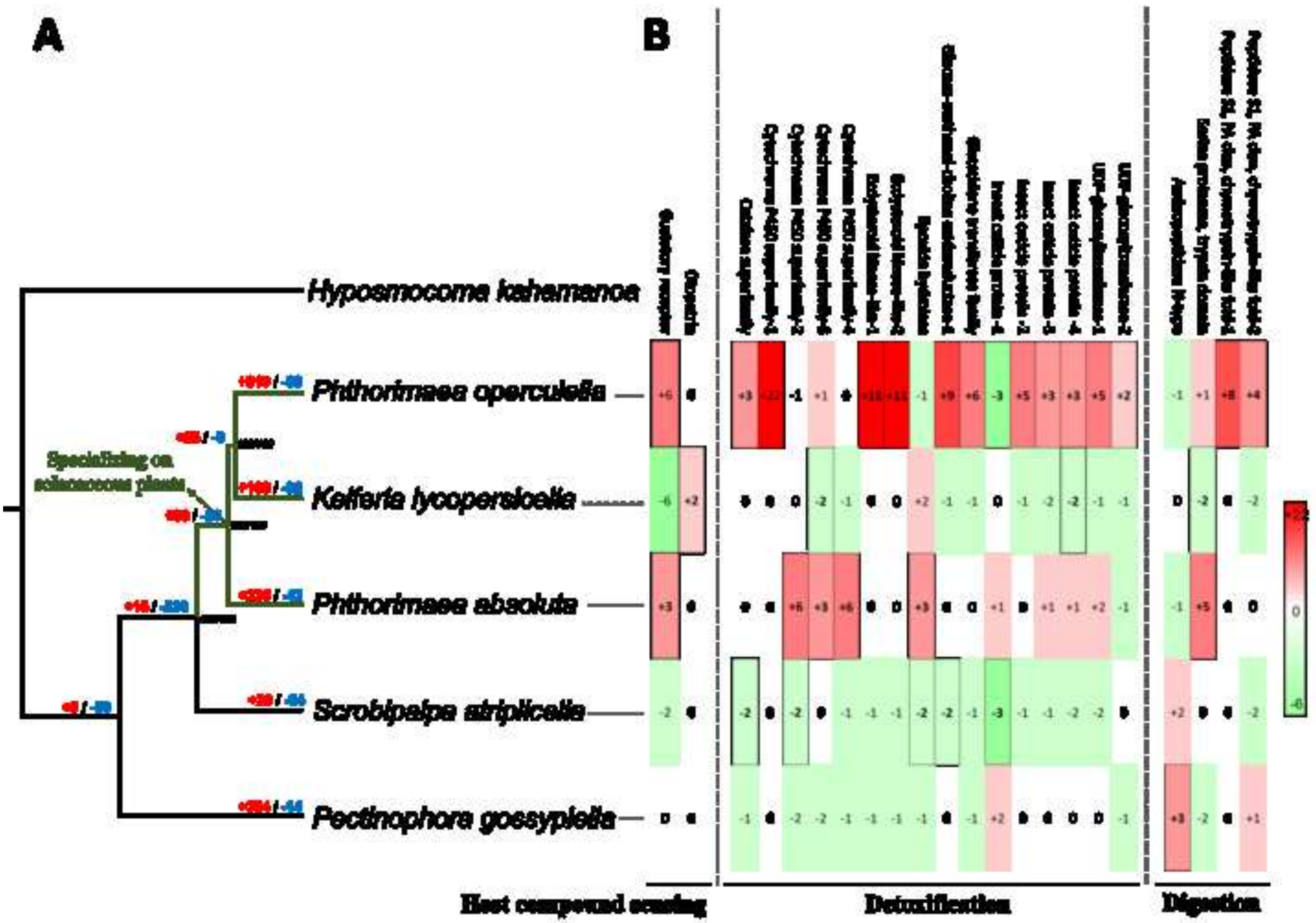

Supplementary method and figures

Click here to access/download

Supplementary Material

Supplemental methods.docx

Table S1

Click here to access/download
Supplementary Material
Table_S1_gFACs_results.xlsx

Table S2

Click here to access/download
Supplementary Material
Table_S2_list_of_rapidly_evolve_HOGs.xlsx

Table S3

Click here to access/download
Supplementary Material
Table_S3_immunity_HOGs.xlsx

Table S4

Click here to access/download
Supplementary Material
Table_S4_host_adaptation_HOGs.xlsx

Table S5

Click here to access/download
Supplementary Material
Table_S5_enriched_GO_summary.xlsx
