# Supplementary material for: Evolutionary genomics of three agricultural pest moths reveals rapid evolution of host adaptation and immune-related genes
Source: Gigascience. 2024 Jan 2;13:giad103. doi: 10.1093/gigascience/giad103 (PMC10759296; doi:10.1093/gigascience/giad103)
Supplement: giad103_GIGA-D-23-00053_Revision_4 [file giad103_giga-d-23-00053_revision_4.pdf]

GigaScience

Evolutionary genomics of three agricultural pest moths reveals rapid evolution of host adaptation and immune-related genes

--Manuscript Draft--

**Manuscript Number:**	GIGA-D-23-00053R4
**Full Title:**	Evolutionary genomics of three agricultural pest moths reveals rapid evolution of host adaptation and immune-related genes
**Article Type:**	Research
**Funding Information:**	USDA APHIS (AP21PPQS&T00C030) — Prof. Akito Y. Kawahara
**Abstract:**	Background<br>Understanding the genotype of pest species provides an important baseline for designing integrated pest management (IPM) strategies. Recently developed long-read sequence technologies make it possible to compare genomic features of non-model pest species to disclose the evolutionary path underlying the pest species profiles. Here we sequenced and assembled genomes for three agricultural pest gelechiid moths: Phthorimaea absoluta (tomato leafminer), Keiferialycopersicella (tomato pinworm), and Scrobipalpa atriplicella (goosefoot groundling moth). We also compared genomes of tomato leafminer and tomato pinworm with published genomes of Phthorimaea operculella and Pectinophora gossypiella to investigate the gene family evolution related to the pest species profiles.<br><br>Results<br>We found that the three solanaceous feeding species, P. absoluta, K.lycopersicella, and P. operculella are clustered together. Gene family evolution analyses with the four species show clear gene family expansions on host plant associated genes for the three solanaceous feeding species. These genes are involved in host compound sensing (e.g., gustatory receptors), detoxification (e.g., ABC transporter C family, Cytochrome P450, Glucose-methanol-choline oxidoreductase, Insect cuticle proteins, and UDP-glucuronosyl), and digestion (e.g., serine proteases and peptidase family S1). A gene ontology enrichment analysis of rapid evolving genes also suggests enriched functions in host sensing and immunity.<br><br>Conclusions<br>Our results of family evolution analyses indicate that host plant adaptation and pathogen defense could be important drivers in species diversification among gelechiid moths.
**Corresponding Author:**	Yi-Ming Weng, Ph.D<br>Florida Museum of Natural History<br>Gainesville, Florida UNITED STATES
**Corresponding Author Secondary Information:**	
**Corresponding Author's Institution:**	Florida Museum of Natural History
**Corresponding Author's Secondary Institution:**	
**First Author:**	Yi-Ming Weng, Ph.D
**First Author Secondary Information:**	
**Order of Authors:**	Yi-Ming Weng, Ph.D
	Shashank R. Pathour, Ph.D
	Keating R. Godfrey, Ph.D
	David Plotkin, Ph.D
	Brandon M. Parker

Powered by Editorial Manager® and ProduXion Manager® from Aries Systems Corporation

	Tyler Wist, Ph.D<br>Akito Y. Kawahara
**Order of Authors Secondary Information:**	
**Response to Reviewers:**	This is the revised version for adding the citation to the GigaDB.
**Additional Information:**	
**Question**	**Response**
Are you submitting this manuscript to a special series or article collection?	No
**Experimental design and statistics**<br><br>Full details of the experimental design and statistical methods used should be given in the Methods section, as detailed in our Minimum Standards Reporting Checklist. Information essential to interpreting the data presented should be made available in the figure legends.<br><br>Have you included all the information requested in your manuscript?	Yes
**Resources**<br><br>A description of all resources used, including antibodies, cell lines, animals and software tools, with enough information to allow them to be uniquely identified, should be included in the Methods section. Authors are strongly encouraged to cite Research Resource Identifiers (RRIDs) for antibodies, model organisms and tools, where possible.<br><br>Have you included the information requested as detailed in our Minimum Standards Reporting Checklist?	Yes
**Availability of data and materials**<br><br>All datasets and code on which the conclusions of the paper rely must be either included in your submission or deposited in publicly available repositories (where available and ethically	Yes

Powered by Editorial Manager® and ProduXion Manager® from Aries Systems Corporation

appropriate), referencing such data using a unique identifier in the references and in the “Availability of Data and Materials” section of your manuscript.<br><br>Have you have met the above requirement as detailed in our Minimum Standards Reporting Checklist?	

Manuscript

Click here to access/download;Manuscript;GIGA-D-23-00053_for_production.docx

Manuscript for resubmission to: GigaScience
(GIGA-D-23-00053)

Title: Evolutionary genomics of three agricultural pest moths reveals rapid evolution of host adaptation and immune-related genes

Yi-Ming Weng[1]*, Pathour R. Shashank[1,2]*, R. Keating Godfrey[1], David Plotkin[1], Brandon M. Parker[1], Tyler Wist[3], Akito Y. Kawahara[1]

* Yi-Ming Weng and Pathour R. Shashank are co-first authors

[1] McGuire Center for Lepidoptera & Biodiversity, Florida Museum of Natural History, University of Florida, Gainesville, Florida, USA
[2] Division of Entomology, ICAR-Indian Agricultural Research Institute, Pusa, New Delhi 110012, India
[3]Agriculture and Agri-Food Canada, 107 Science Place, Saskatoon, SK, S7N 0X2, Canada

Corresponding author: Akito Y. Kawahara; kawahara@flmnh.ufl.edu

ORCID identifier:

Yi-Ming Weng: 0000-0002-8243-5061

Shashank R. Pathour: 0000-0002-8177-6091

R. Keating Godfrey: 0000-0001-8740-1752

David Plotkin: 0000-0002-2339-655X

Tyler Wist: 0000-0003-3820-2487

Akito Y. Kawahara: 0000-0002-3724-4610

Abstract

Background

Understanding the genotype of pest species provides an important baseline for designing integrated pest management (IPM) strategies. Recently developed long-read sequence technologies make it possible to compare genomic features of non-model pest species to disclose the evolutionary path underlying the pest species profiles. Here we sequenced and assembled genomes for three agricultural pest gelechiid moths: *Phthorimaea absoluta* (tomato leafminer), *Keiferia lycopersicella* (tomato pinworm), and *Scrobipalpa atriplicella* (goosefoot groundling moth). We also compared genomes of tomato leafminer and tomato pinworm with published genomes of *Phthorimaea operculella* and *Pectinophora gossypiella* to investigate the gene family evolution related to the pest species profiles.

Results

We found that the three solanaceous feeding species, *P. absoluta*, *K. lycopersicella*, and *P. operculella* are clustered together. Gene family evolution analyses with the four species show clear gene family expansions on host plant associated genes for the three solanaceous feeding species. These genes are involved in host compound sensing (e.g., gustatory receptors), detoxification (e.g., ABC transporter C family, Cytochrome P450, Glucose-methanol-choline oxidoreductase, Insect cuticle proteins, and UDP-glucuronosyl), and digestion (e.g., serine proteases and peptidase family S1). A gene ontology enrichment analysis of rapid evolving genes also suggests enriched functions in host sensing and immunity.

Conclusions

Our results of family evolution analyses indicate that host plant adaptation and pathogen defense could be important drivers in species diversification among gelechiid moths.

Keywords: Gelechiidae, twirler moths, genome assembly, host adaptation, detoxification, immunity, tomato leafminer, tomato pinworm, goosefoot groundling moth

Background

Gelechiidae are a diverse family of Lepidoptera comprised of more than 4,700 species [1, 2]. Some species, such as *Phthorimaea absoluta* (tomato leafminer), *Keiferia lycopersicella* (tomato pinworm), and *Phthorimaea operculella* (potato tuber moth), are notorious agricultural pests which could cause more than a billion U.S. dollars of annual agricultural damage globally [3-10]. The three gelechiids all use solanaceous plants as larval hosts, but *Phthorimaea absoluta* and *Keiferia lycopersicella* feed primarily on tomato while *Phthorimaea operculella* prefers potato. These species especially the two *Phthorimaea* species are found invading many non-native regions including Asia, Europe, and Africa. Research on these moths have focused largely on their host preference, identification, and management. Despite their importance as major global pests to agriculture, their genomic framework and the evolutionary process of host plant preference in insect pests is still poorly understood (but see [11]).

Host selection and host use in insects is determined by a series of physiological processes including host plant compound sensing, detoxification, and nutrient digestion. Several genes are thought to be involved in these processes that affect host selection [12]. Genes associated with sensing phytocompounds include olfactory receptors (OR), gustatory receptors (GR), ionotropic receptors (IR), odorant-binding proteins (OBP), and chemosensory proteins (CSP). Genes associated with detoxification include cytochrome P450 (P450), ATP-binding cassette transporter (ABC), and glutathione S-transferases (GST), and genes associated with digestion include serine protease (SP) and beta-fructo-furanosidases (BFF) [13-21]. A crucial question in understanding pest evolution is how these genes evolved among pest species and their relatives. Whole genome sequencing of pest species has shown great promise for revealing the evolutionary processes that led to the formation of a pestiferous species. For example, recent studies on the genomic evolution of agricultural pests, with subsequent comparative genomics analyses such as orthology, gene family evolution, selected region detections, and structural variant analyses, have identified putative genetic bases of their ecological features or pest species profiles [22-25].

Despite the diversity of gelechiid moths, the many studies on the impact of gelechiids to agriculture, and the release of nearly a thousand Lepidoptera genome assemblies in GenBank thus far [26], only a few gelechiid genome assemblies are publicly available [11, 27, 28]. Considering its high species diversity and economic importance, more attention and efforts on genomic data accumulation and exploration are required for further understanding the evolution of this moth

family. In this study, we sequenced and assembled the genomes of three gelechiid moth pests, *Keiferia lycopersicella*, *Phthorimaea absoluta*, and *Scrobipalpa atriplicella* to examine their genomic features and how they relate to host preference. Specifically, we investigate how rapidly evolving genes are correlated with host preference and life history.

Methods

Sample information and sequencing

Three gelechiid moth species (*K. lycopersicella* [NCBI:txid1511203], *P. absoluta* [NCBI:txid702717], *S. atriplicella* [NCBI:txid687131]) were collected from laboratory colonies at University of California, Davis, USA, Khumaltar, Lalitpur, Nepal, and the Saskatoon Research and Development Centre of Agriculture and Agri-Food, Canada, respectively. Genomic DNA from one moth of each species was extracted from the whole moth (larva) using the DNA isolation protocol of the OmniPrep Genomic DNA Extraction Kit (G-Biosciences, St. Louis, MO). For *S. atriplicella*, we encountered sequencing interference for several library samples. Therefore, we amplified genomic DNA with illustra™ GenomiPhi V2 DNA Amplification Kits, Cytiva and the amplified DNA was used to replace the native DNA extracted from the tissue. The genomic and amplified DNA samples were subsequently used to perform fragment size selection and sample purification with the DNeasy PowerClean CleanUp Kit before library preparation. Libraries were sequenced with a single SMRT cell in the Pacbio Sequel IIe system. The DNA clean-up, library construction, and sequencing steps were performed in the Interdisciplinary Center for Biotechnology Research (ICBR) at the University of Florida. The HiFi sequences are deposited in NCBI (BioProject accession number: PRJNA932016; SRA sample accession: SRR23497930, SRR23497929, and SRR23497928).

Genome size and sequence coverage estimations

To verify read quality, we first assessed the HiFi sequence quality using FastQC v 0.11.7 (RRID:SCR_014583) to summarize read profiles [29]. The genome size and sequence coverage were estimated with two methods. First, we counted k-mers and calculated the k-mer density distribution for the HiFi reads using K-Mer Counter (KMC) v.3.2.1 (RRID:SCR_001245) with k-mer size of 31 nucleotides. Density distributions were subsequently submitted to GenomeScope v2.0 online tool (RRID:SCR_017014) [30] with default setting for diploid species to estimate the

genome size, heterozygosity, sequence coverage and other genomic profiles (**Supplementary Figure S1**). Second, we mapped HiFi reads to final assemblies to estimate genome size and sequence coverage. This process was conducted in the program MODEST (backmap.pl v 0.5) [31]. Estimated genome sizes and read coverages from GENOMESCOPE were used to certify autodetected estimates from HIFIASM assembler (see next section) to ensure the accuracy of autodetected assembling assumptions [32].

Genome assembly, quality assessment, and non-target sequence removal

We used HIFIASM v 0.16.1 (RRID:SCR_021069) to assemble the genome from HiFi reads using default settings, except for reads of *P. absoluta*, for which we applied a 2 (-l 2) purging level to keep a greater number of haplotigs for downstream purging. We kept more haplotigs because the sequence coverage for this species was low, and it generated the best assembly evaluated by N50 and BUSCO v 5.3.0 (RRID:SCR_015008) completeness (based on the lepidoptera_odb10 database) [33, 34]. We also applied the haplotig purging pipeline to remove duplicated haplotigs [35]. For *K. lycopersicella* and *S. atriplicella*, we first mapped the HiFi reads to the assemblies with MINIMAP v. 2.21 (RRID:SCR_008103) and sorted using SAMTOOLS v 1.15 (RRID:SCR_002105) [36, 47]. The sorted mapped reads were subsequently used to draw the density distribution histogram of coverage using "hist" function in the purge_haplotigs pipeline (RRID:SCR_017616) [34]. The histogram was used to identify the peaks of homozygous and heterozygous reads and the low point between the peaks. These values were then used to define the aggressiveness of the purging. Finally, we used low and high coverage cutoffs to purge the duplicated contigs. Other parameters were kept default as suggested by purge_haplotigs pipeline. For *P. absoluta*, since its sequence depth is relatively low (see results), we used the Illumina short reads published by [28] to perform the purge_haplotigs. Specifically, we ran HIFIASM with less aggressive purging (l -2) to allow more duplicated haplotypes in the assembly for the haplotig purging pipeline and used the short-read coverage histogram to define the peaks. To identify potential non-target sequences in assemblies, we created blobplots using BLOBTOOLS (RRID:SCR_017618) to visualize the distribution of GC content and read coverage for contigs [37]. To determine read coverage, we aligned HiFi reads to the assembly using MINIMAP2 [36]. To assign taxonomy to reads, we used BLASTN (RRID:SCR_001598) to blast contigs against the NCBI nt database with an e-value cutoff of 1e-25. Contigs assigned to non-arthropods with deviating GC

content and sequence coverage were determined to be non-target sequences and removed from assemblies (**Supplementary Figure S2**). A BUSCO score using lepidoptera_odb10 database was calculated to evaluate the completeness of each assembly (**Table 1**). Genome assemblies of the three species are available through NCBI (BioProject accession number: PRJNA932016).

Gene models and annotations

In the genome annotation pipeline, we first identified repeat regions using REPEATMODELER2 (RRID:SCR_015027) [38]. The genome assemblies were soft-masked with repeats from three lines of evidence including simple and short repeats, the identified repeats from REPEATMODELER2, and the evidence from the lepidopteran repeat database in Repbase using REPEATMASKER (RRID:SCR_012954) with the blast tool RMBLAST (RRID:SCR_022710) [39, 40]. The BRAKER2 gene prediction pipeline (RRID:SCR_018964) was applied to soft-masked genomes [41-47]. For *K. lycopersicella* and *S. atriplicella*, we used arthropod protein sequences from orthoDB (RRID:SCR_011980) (odb10_arthropoda) in the PROTHINT pipeline (RRID:SCR_021167) to generate hints to train GENEMARK-EP+ (RRID:SCR_011930) [48] and predict gene models alone with the AUGUSTUS (RRID:SCR_008417). For *P. absoluta*, we also included published RNA sequences to train the gene model [49]. Specifically, we ran BRAKER2 pipeline twice (one with protein and one with RNA) and used TSEBRA [50] with default settings to integrate the two models. To further refine models for the three species, we removed genes identified solely by AUGUSTUS *ab initio* prediction without hint supports (e.g., introns, start and stop codons) from the protein database using the python script "selectSupportedSubsets.py" provided by BRAKER2. Final gene models were evaluated using a BUSCO protein model with the lepidoptera_odb10 database. Gene model profiles, including the monoexonic rate and sequence lengths of gene, intron, and exon, were summarized using GFACS v1.0.0 (RRID:SCR_022017) [51] (**Supplemental Table S1**).

For functional annotations, we first annotated gene function by blasting transcript sequences from the BRAKER2 pipeline to the RefSeq non-redundant protein database and Swiss-Prot arthropodan protein database (Reviewed UniPort database) using the blastp function in DIAMOND v2.0.9 (RRID:SCR_016071) [43]. Additionally, we performed default INTERPROSCAN (RRID:SCR_005829) annotation which integrates 14 member databases including PFAM and PANTHER [52]. For gene ontology terms (GO terms) and KEGG pathway annotations

(RRID:SCR_012773), we queried transcript sequences to the PANNZER webserver (Protein annotation with z-score) [53] and KEGG automatic annotation server (KAAS) [54] with bi-directional best hits.

Phylogeny and Gene evolution

To explore the evolution of the three gelechiid moths and their genes, we created a phylogeny using two additional published genomes of Gelechiidae: *Phthorimaea operculella* and *Pectinophora gossypiella*. We used the genome of *Hyposmocoma kahamanoa* as an outgroup, as this species belongs to a moth family closely related to Gelechiidae (Cosmopterigidae) [55, 56]. Published genome assembly of *P. operculella* (GCA_024500475.1) was downloaded from NCBI GenBank while those of *P. gossypiella* (GCF_024362695.1) and *H. kahamanoa* (GCF_003589595.1) were downloaded from the NCBI Reference Sequence (RefSeq) Database (O'Leary et al., 2016). We performed the same BUSCO approach using the lepidoptera_odb10 database to obtain compatible single-copy amino acid orthologs for these three species [32]. The final data matrix contained 4,876 single copy orthologs that contained at least two ingroup species and the *H. kahamanoa* outgroup (385 orthologs did not fit these parameters and were removed). Sequences of each ortholog were aligned using default settings in MAFFT version 7.490 (RRID:SCR_011811) [57].

Phylogeny of these five gelechiid moth species was constructed using the concatenated sequences from the BUSCO single copy gene alignments. We assigned a single substitution model (Q.insect+FO+G4 substitution model, the Q matrix estimated for insects) to the alignment and built a maximum likelihood tree in IQ-tree v 2.1.3 (RRID:SCR_017254) [58-61]. Branch supports were calculated using ultrafast bootstrap [62] and SH-aLRT [63-64]. Since the genome assembly of *S. atriplicella* is less complete (see results), we also constructed phylogeny with the four gelechiid moth species (excluding *S. atriplicella*) and *H. kahamanoa* (outgroup) for gene family analysis (see below) to avoid the noise from the incomplete gene model of *S. atriplicella* with same tree building approach.

To investigate gene family evolution, we inferred an ultrametric tree from the concatenated sequence species tree using TREEPL with default settings [65]. For gene family identification, we employed ORTHOFINDER v2.5.2 (RRID:SCR_017118) using the primary isoform of the annotated gene models from each of the five species (four gelechiid species and one outgroup) [66]. Gene

models of *K. lycopersicella* and *P. absoluta* were predicted from the BRAKER2 pipeline while the other three gene models were directly downloaded from appropriate databases. In ORTHOFINDER, we chose gene families as defined by phylogenetic hierarchical orthogroups (HOGs), an approach which is thought to be more accurate than similarity-based methods [66]. For each gene family, the HOGs gene-counting matrix and ultrametric tree were used to estimate repertoire size changes in CAFE v 5.0.0 (RRID:SCR_018924) [67]. We extracted HOGs under rapid repertoire size expansion and contraction, with the significance level set to 0.01 and branch lengths calculated from the ultrametric tree. For each gene associated with these HOGs, we used the top annotated function (lowest e-value) from INTERPROSCAN to represent the gene function.

For the HOGs with significant rapid expansion and contraction, we assessed their gene functions and GO terms using INTERPROSCAN annotations. To standardize annotations, we reannotated gene functions for the three downloaded gene models (*Phthorimaea operculella*, *P. gossypiella*, and *H. kahamanoa*) using default settings in INTERPROSCAN (Jones et al., 2014). For associated GO terms, we performed enrichment analysis using the R package TOPGO 2.40.0 (RRID:SCR_014798) [68] with a significance level of 0.05 for both fisher classic and weight01 algorithms.

Results

Genome assemblies and annotations

To assemble genomes of *K. lycopersicella*, *P. absoluta*, and *S. atriplicella*, we used 3.8, 2.2, and 2.9 million PacBio high-fidelity (HiFi) reads (mean read lengths are 5.5, 4.9, and 7.6 kbp, respectively) corresponding to the estimated read coverage of 50X, 20X, and 37X, respectively. After haplotig removal, considerable reductions in the number of contigs were found while assembled sizes and BUSCO completeness remained nearly consistent, indicating that smaller duplicated contigs were removed. From the assemblies of *K. lycopersicella* and *S. atriplicella*, we identified non-target sequences contributing to a small portion of the assemblies. In *K. lycopersicella*, a 10 kbp-contig was blasted to Streptophyta while in *S. atriplicella*, 15 small contigs (total 380 kbp) were blasted to Proteobacteria. After removing these non-target contigs, 443.65 Mb from 61 contigs, 652.7 Mb from 688 contigs, and 301.15 Mb from 7,092 contigs were found in the assemblies of *K. lycopersicella*, *P. absoluta*, and *S. atriplicella*, respectively. BUSCO scores for these assemblies are shown in **Table 1**. Estimated genome sizes from GENOMESCOPE

for *K. lycopersicella*, *P. absoluta*, and *S. atriplicella* were 396, 514, and 276 million base pairs (Mb); much smaller than our assemblies and likely due to the exclusion of extremely high number of k-mers from the repeats in the genomes. Estimating genome size with ModEst resulted in 422, 1,040, and 344 Mbs with peak coverages at 49X, 10X, and 55X for *K. lycopersicella*, *P. absoluta*, and *S. atriplicella*, respectively (**Supplemental Table S2**). The estimated genome and assembly sizes of *P. absoluta* based on ModEst were nearly twice that of GenomeScope, this discrepancy was likely due to different estimated sequence coverage.

For gene annotation, we first annotated repeats using RepeatModeler2 [38] and *P. absoluta* showed the highest proportion of repeats (54.4%), followed by *K. lycopersicella* (48.22%) and *S. atriplicella* (32.83%). Soft-masked genomes were used to run the Braker2 pipeline with protein evidence for *K. lycopersicella* and *S. atriplicella*, resulting in 15,405 and 14,647 genes, respectively [42]. For *P. absoluta*, we used both protein and RNA sequence evidence to predict the gene model. After removing genes without hint support, the gene model with 19,106 genes was used for functional annotation. BUSCO scores for these gene models reflect their assembly features, including the higher duplication rate in *P. absoluta* and higher missing rate in *S. atriplicella* (**Table 1**).

Phylogeny and gene family evolution

The maximum likelihood tree, derived from the concatenated supermatrix, shows that *K. lycopersicella* and *P. operculella* are most closely related (**Figure 1**). *Phthorimaea absoluta*, another species feeding on solanaceous hosts, is the sister species to *K. lycopersicella* and *P. operculella*. It is noteworthy that *P. absoluta* was previously and widely recognized as *Tuta absoluta* indicating a need of a comprehensive phylogenomic analysis on this group. *Scrobipalpa atriplicella*, an amaranthaceous feeder, is recovered as the sister taxon to the other three members of subfamily Gelechiinae in the six-species phylogeny (**Supplementary Figure S3**). Finally, *Pectinophora gossypiella*, a member of subfamily Apatetrinae, is the sister taxon to all four Gelechiinae species in the phylogeny, supporting the current taxonomic arrangement [69] (**Figure 1**).

We identified 14,384 HOGs in the protein sequences of the six species, and 809 HOGs were found to evolve rapidly at least in one branch along the ultrametric tree (**Supplemental Table S3**). Gene family evolution analyses showed a general pattern of rapid expansions at the tips of

the tree and rapid contractions along internal branches (**Figure 1**). Specifically, 85 and 52 HOGs were identified in *K. lycopersicella* with rapid repertoire size expansion and contraction, respectively. Among these HOGs, 67 are annotated with gene functions where 5 are putatively involved in host plant adaptation (Glucose-methanol-choline oxidoreductase, Cytochrome P450 superfamily, insect cuticle proteins, and trypsin family serine proteases), 37 involved in immunity (*PiggyBac* transposable elements, Retrotransposon *Pao*-related genes, Serpin superfamilies, and Toll-like receptors (**Supplemental Tables S4 and S5**).

For *P. operculella*, 368 HOGs (310 expansions and 66 contractions) were identified to evolve rapidly where 166 HOGs (158 expansions and 8 contractions) are annotated with gene functions. From these HOGs, we found that 16 are putatively associated with host plant adaptation including Gustatory receptor, Catalase superfamily, Cytochrome P450 superfamily, Ecdysteroid kinases, Glucose-methanol-choline oxidoreductase, Insect cuticle proteins, and UDP-glucuronosyl. We also found that 68 immune related associated HOGs (Gamma interferon related gene, Pacifastin domain, Serpin superfamily, *PiggyBac* transposable element, Retrotransposon *Pao*-related genes, and *Ty3* transposon) and one pheromone signaling related gene (CD36 family). For *P. absoluta*, 150 HOGs (135 expansions and 15 contractions) were identified to evolve rapidly, and among them, 102 HOGs (94 expansions and 8 contractions) are annotated with gene functions. These HOGs include 9 host plant adaptation related genes (Cytochrome P450 superfamilies, Epoxide hydrolase, Ecdysteroid kinases, and Serine proteases) and 39 immune related genes (Immunoglobulin, Pacifastin, Retrotransposon *Pao*-related genes, *PiggyBac* transposable element, and *Toll*-like receptor). Finally, we found 137 (135 expansions and 20 contractions) rapidly evolving HOGs along the branch of *P. gossypiella*, where 101 HOGs have gene annotations (90 expansions and 11 contractions). Among them, 36 HOGs are associated with immunity (*PiggyBac* transposable elements, Retrotransposon *Pao*-related genes, and *Toll*-like receptors. We also found 5 (2 expansions and 3 contractions) host plant adaptation related genes (Cytochrome P450 superfamilies, Catalase superfamily, and peptidase family S1). A summary of HOGs with putative functions in immunity and host adaptation are listed in **Supplemental Tables S4 & S5**, respectively.

Gene ontology enrichment analyses

From genes that were identified to be rapidly evolving, we found a handful of biological function terms that were enriched from the rapidly evolving genes. (**Table 2**). This includes sensory perception of taste (GO:0050909), toll-like receptor signaling pathway (GO:0002224), and immune response (GO:0006955), DNA integration (GO:0015074), and plasma membrane phospholipid scrambling (GO:0017121). All the enriched terms, including those terms passing through fisher classic but not weight01 threshold, are listed in **Supplemental Table S6**.

Discussion

Genome assembly quality and its implications for gene family evolution

Long read sequencing technologies such as Pacific Biosciences (PacBio) and Oxford Nanopore Technologies (ONT) have provided a promising future for *de novo* assemblies of high-quality genomes for non-model species [70, 71]. These recent advancements have the potential to significantly expand our understanding of the evolutionary mechanisms underlying plant–insect interactions and contribute to prevent future catastrophic crop damage.

In this study, we used HiFi long-read to assemble genomes for three gelechiid moth species (**Table 1**). Although BUSCO completeness of the *S. atriplicella* genome was relatively low (73.3%), 3,246 of its BUSCO genes could be used to reconstruct a phylogeny with four other gelechiid species (**Supplemental Figure S3**). However, we note that the robustness of gene family evolution analyses relies heavily on the quality of the genome assembly and the subsequent gene model predictions. The incomplete genome assembly with higher heterozygosity or lower sequence coverage could confound the result by providing missing, incomplete, or duplicated gene prediction. Therefore, we used phylogeny with the four more complete genome assemblies and an outgroup to detect gene-family evolution with rapid repertoire size changes. We note that the assembly of *P. absoluta* has higher BUSCO duplication rate, likely the result of shallower sequence depth and higher heterozygosity, could possibly overestimate the gene copy number. Although programs such as CAFE were designed to cope with such issues [72], the result of repertoire size changes for species with lower assembly completeness should be interpreted with some caution.

It should also be noted that sequencing interferences were encountered for *S. atriplicella* library samples, which were prepared together with those of *P. absoluta* and *K. lycopersicella* using the same DNA extraction, clean-up, library preparation, and sequencing protocols. We

therefore sequenced the amplified DNA library with trial-and-error. According to the BUSCO completeness score, only part of the genome was covered by the HiFi reads despite the high sequence depth (37X and 55X from GENOMESCOPE and MODEST, respectively). This is likely due to replication bias or errors during the amplification process. Based on these experiences, we would recommend optimization of DNA extraction to enable native DNA to be sequenced over the whole genome amplification route we followed in with this challenging sample.

Finally, the different methods and assembled sizes resulted in considerable differences in estimated genome sizes. This discrepancy seems to be more prominent in taxa with lower sequence coverage and higher heterozygosity (e.g., *P. absoluta*). We therefore recommend using multiple approaches to better estimate the true genome size and understand the quality of the assembly.

Genomic adaption of the solanaceous feeding gelechiid moths

Moths use a combination of olfactory (smell) and gustatory (taste or contact) chemoreception to find oviposition sites. Olfactory and gustatory cues are often thought to function in long- and short-range detection of suitable hosts, respectively, but volatile cues at the host surface may also stimulate olfactory sensilla and determine oviposition choice in some species [73]. Indeed, for *P. absoluta,* olfactory cues in the form of tomato leaf volatiles result in oviposition rates indistinguishable from to those involving direct contact with the leaf surface [74]. This is not the case for *K. lycopersicella* and *P. operculella* where contact chemoreception appears to play a more important role in oviposition choice, with surface compounds of host plants shown to stimulate egg-laying in both species [75-77], and those of non-hosts shown to act as deterrents in *P. operculella* [76]. It is notable that our gene family evolution analyses show an increase in rapidly evolving genes associated with host plant sensing, particularly gustatory receptors [78], coincident with a shift to solanaceous feeding in gelechiid moths. This could serve as an indication of selective pressure on host plant association through female oviposition or larval feeding choice. It is likely that caterpillars of leaf-rolling and leaf-mining species are confined to the plant where they hatch [79, 80] and therefore do not search extensively for a new host plant, but the role of contact chemoreception in oviposition choice in gelechiids is better characterized than that of caterpillar host searching.

While an increase in host plant association genes correlates with a shift to feeding on Solanaceae, we do not observe a directional shift in terms of gains/losses. Thus, while we detected

gains in gustatory receptor genes in *P. absoluta* and *P. operculella*, *K. lycopersicella* shows losses in this gene family. While a number of studies have shown a correlation between host range and chemosensory receptor gene repertoire size or specific losses [81-83], the gelechiids studied here appear to have experienced a host shift from one plant family (Amaranthaceae in *S. atriplicella*) to another (Solananceae in *P. absoluta* and its relatives), instead of an expansion or contraction of host range. Thus, we might not expect directional changes in chemosensory receptor repertoire size in instances of host shifts in the same manner that has been observed after expansion or contraction of host range.

For many lepidopteran species, detoxification of plant secondary metabolites is essential in host adaptation [84]. Several genes, including ABC transporters, P450, GMC oxidoreductase, UGT, and insect cuticle protein play important roles in detoxifying the defending compounds from their host plants [85, 86]. Our gene family evolution analysis reveals that these detoxification genes also rapidly evolve in the focal gelechiid moths, while most expansions are found in the two solanaceous feeding species (i.e., *P. absoluta* and *P. operculella*) (**Figure 1**). It is noteworthy that *K. lycopersicella*, the sister species of *P. operculella* in our tree, shows only four detoxification genes expanding. This result may be explained partially by the different annotation pipelines that were used for these two genomes. However, since the gene model of *K. lycopersicella* covers 93.2% of the BUSCO single copy genes and CAFE was designed controlling such confounding factors from the incomplete or biased annotation, it is fair to conclude that detoxification gene expansion is not a general feature of solanaceous-feeding species [72]. Interestingly, *K. lycopersicella* and *P. absoluta* are found to prefer tomato over potato while *P. operculella* feeds mainly on potato, implying that the feeding and oviposition preferences are not directly related to the evolution of detoxification genes. One other possible explanation is that the rapid expansion of detoxification genes on *P. absoluta* and *P. operculella* has resulted from the frequent exposure to pesticides, as these two species are well-known agriculture pests with many pesticide resistances reported [87-89]. Although *K. lycopersicella* is also considered an agricultural pest, the damage it causes is not comparable to the two *Phthorimaea* species [7]. Further studies using population genomic approaches to determine the relationship between detoxification-gene evolution and pesticide resistance might provide more evidence supporting or opposing this hypothesis.

One other important mechanism in host adaptation involves digesting nutrients from plant tissue. For many phytophagous insects, coping with host plant protein peptidase inhibitors and

efficiently breaking down these complex molecules are an essential first step in digestion [20]. By comparing genomes of four gelechiid species, we identified many serine protease genes which are known for its function of host-plant protein digestion. According to the gene evolution results, serine protease genes rapidly expanded in *P. absoluta* and *P. operculella*, and, *P. gossypiella* (**Figure 1**). These genes not only digest proteins, they also act as species-specific antagonists interfering with the function of host plant peptidase inhibitors [94-96]. The expansion of trypsin and chymotrypsin in two global pests on solanaceous crops implies their underlying contributions to important pest species features such as shorter life spans relative to *K. lycopersicella*, a species that has fewer copies of these genes [7, 97-100]. In general, our gene family evolution analysis reveals indirect but important signals of genome evolution underlying the host adaptation in these agricultural pests.

Rapid evolution of retrotransposable elements and other immune related genes in gelechiid moths

We found that many of the rapidly evolving genes present in all four gelechiid species are genes associated with retrotransposons and reverse transcription. For example, HOGs annotated with *Pao*, a retrotransposable element involved in antiviral mechanism, were found to be rapidly evolving in all four gelechiid species (**Supplemental Table S3**). This element usually contains five protein domains where reverse transcriptase (RTase), retrotransposon gag domain, aspartic protease (or aspartic peptidase), and Ribonuclease H superfamily (RNase H) are repeatedly found evolving rapidly [101, 102]. The RTase in this retrovirus-like element reverse-transcribes the invading virus RNA into DNA (stored in retrotransposon sequences or forming a viral circular DNA), and the infection is suppressed by RNase H through cleavage of the DNA-RNA hybrids or by the downstream RNAi pathway [103-107]. Many other significant HOGs found in these gelechiid species may also have similar antiviral mechanisms, including *PiggyBac* transposable element, *Ty3* transposon capsid-like protein, and Transposase, L1 [108, 109] (**Supplemental Table S3**). However, rapid repertoire size changes of these retrotransposable elements could be the result of the transposon activity instead of gene copy accumulation through recombination.

We also found many rapidly evolving HOGs annotated with immune related genes such as those involved in the Toll-like receptor (TLC) pathway. These genes (e.g. Toll-like receptor, Leucine-rich repeat domain superfamily, and NF-kappa-B inhibitor-interacting Ras-like protein) were found with rapid size changes in all tested gelechiid species. Unlike retrotransposable

elements, the TLC pathway targets a wider range of pathogens including bacteria, fungi, and viruses. Finally, many other genes that we identified have putative functions in immunity, including Serpin superfamily, Immunoglobulin, Pacifastin domain, and Gamma interferon inducible lysosomal thiol reductase GILT. The presence of many rapidly evolving, immune-related genes suggests that managing potential threats from pathogens is also a significant selection pressure. This finding is supported by comparative genomic studies on other moths where viral defending genes (RNase H, RTase, retrotransposon *Pao*, Toll-like receptor, Leucine-rich repeat domain) were identified to evolve rapidly [11, 110]. In sum, our gene family evolution approach highlights the importance of host adaptation and immune-related genes in these closely related gelechiid species.

Availability of source code

Project name: Genome Assembling of Gelechiid Moths

Project home page: https://github.com/yimingweng/genome_assembling_of_gelechiid_moths

Operating system(s): Linux

Programming language: Bash (Unix shell), python

License: MIT

Data Availability

The data sets supporting the results of this article are available in the NCBI under BioProject PRJNA932016. Supplementary data is also available via the GigaScience database GigaDB [111].

Competing interests

The authors declare that they have no competing interests.

Acknowledgments

We thank USDA APHIS for providing grant funding (grant ID: AP21PPQS&T00C030) to support this study. Thanks also to Dr. N. R. Prasannakumar, ICAR-Indian Institute of Horticultural Research, India for sending samples of *P. absoluta*. Analyses were performed on the HiPerGator high-performance computer (University of Florida). PRS expresses his sincere thanks to Dr. Ashok

Kumar Singh, Director, Indian Agricultural Research Institute and Indian Council of Agricultural Research, New Delhi, India for necessary permissions and their support. This research was made possible, in part, by a Cooperative Agreement (AP21PPQS&T00C030) from the United States Department of Agriculture's Animal and Plant Health Inspection Service (APHIS). It may not necessarily express APHIS' views. Mention of trade names or commercial products in this publication is solely for the purpose of providing specific information and does not imply recommendation or endorsement by the USDA.

Author Contributions

AYK, BMR, DP, PRS, and TW, developed the project; PRS and YMW conducted experiments and analyses; RKG and YMW wrote the first draft of the manuscript; AYK, BMR, DP, PRS, and TW, provided comments to improve the manuscript.

References

1. Karsholt O, Mutanen M, Lee S and Kaila L. A molecular analysis of the Gelechiidae (Lepidoptera, Gelechioidea) with an interpretative grouping of its taxa. Systematic Entomology. 2013;38 2:334-48. doi:https://doi.org/10.1111/syen.12006.
2. Van Nieukerken E, Kaila L, Kitching I, Kristensen N, Lees D, Minet J, et al. Animal biodiversity: An outline of higher-level classification and survey of taxonomic richness. Zootaxa. 2011;3148 1:212-21.
3. Biondi A, Guedes RNC, Wan FH and Desneux N. Ecology, Worldwide Spread, and Management of the Invasive South American Tomato Pinworm, Tuta absoluta: Past, Present, and Future. Annu Rev Entomol. 2018;63:239-58. doi:10.1146/annurev-ento-031616-034933.
4. Chang PEC and Metz MA. Classification of *Tuta absoluta* (Meyrick, 1917)(Lepidoptera: Gelechiidae: Gelechiinae: Gnorimoschemini) based on cladistic analysis of morphology. Proceedings of the entomological Society of Washington. 2021;123 1:41-54.
5. Mori BA, Dutcheshen C and Wist TJ. Scrobipalpa atriplicella (Lepidoptera: Gelechiidae), an invasive insect attacking quinoa (Amaranthaceae) in North America. The Canadian Entomologist. 2017;149 4:534-9. doi:10.4039/tce.2017.19.
6. Trivedi TP and Rajagopal D. Distribution, biology, ecology and management of potato tuber moth, Phthorimaea operculella (Zeller) (Lepidoptera: Gelechiidae): A review. Tropical Pest Management. 1992;38 3:279-85. doi:10.1080/09670879209371709.
7. Poe SL. Tomato Pinworm, *Keiferia lycopersicella* (Walshingham). Citeseer; 1999.
8. Rwomushana I, Beale T, Chipabika G, Day R, Gonzalez-Moreno P, Lamontagne-Godwin J, et al. Tomato leafminer (*Tuta absoluta*): impacts and coping strategies for Africa. CABI Working Paper. 2019;12.
9. Venkatramanan S, Wu S, Shi B, Marathe A, Marathe M, Eubank S, et al. Modeling commodity flow in the context of invasive species spread: Study of *Tuta absoluta* in Nepal. Crop Protection. 2020;135:104736.
10. Soares MA and Campos MR. *Phthorimaea absoluta* (tomato leafminer). CABI International; 2022.
11. Zhang M, Cheng X, Lin R, Xie B, Nauen R, Rondon SI, et al. Chromosomal-level genome assembly of potato tuberworm, Phthorimaea operculella: a pest of solanaceous crops. Sci Data. 2022;9 1:748. doi:10.1038/s41597-022-01859-5.
12. Simon J-C, d'Alençon E, Guy E, Jacquin-Joly E, Jaquiéry J, Nouhaud P, et al. Genomics of adaptation to host-plants in herbivorous insects. Briefings in Functional Genomics. 2015;14 6:413-23. doi:10.1093/bfgp/elv015.
13. Ma L, Li ZQ, Bian L, Cai XM, Luo ZX, Zhang YJ, et al. Identification and Comparative Study of Chemosensory Genes Related to Host Selection by Legs Transcriptome Analysis in the Tea Geometrid *Ectropis obliqua*. PLoS One. 2016;11 3:e0149591. doi:10.1371/journal.pone.0149591.
14. Agnihotri AR, Roy AA and Joshi RS. Gustatory receptors in Lepidoptera: chemosensation and beyond. Insect Molecular Biology. 2016;25 5:519-29. doi:10.1111/imb.12246.
15. Barve PR, Tellis MB, Barvkar VT, Joshi RS, Giri AP and Kotkar HM. Functional Diversity of the Lepidopteran ATP-Binding Cassette Transporters. Journal of Molecular Evolution. 2022;90 3-4:258-70. doi:10.1007/s00239-022-10056-2.
16. Calla B, Noble K, Johnson RM, Walden KKO, Schuler MA, Robertson HM, et al. Cytochrome P450 diversification and hostplant utilization patterns in specialist and generalist moths: Birth, death and adaptation. Molecular Ecology. 2017;26 21:6021-35. doi:https://doi.org/10.1111/mec.14348.
17. Cui WC, Wang B, Guo MB, Liu Y, Jacquin-Joly E, Yan SC, et al. A receptor-neuron correlate for the detection of attractive plant volatiles in *Helicoverpa assulta* (Lepidoptera: Noctuidae). Insect Biochemistry and Molecular Biology. 2018;97:31-9. doi:10.1016/j.ibmb.2018.04.006.

18. Koirala B K S, Moural T and Zhu F. Functional and Structural Diversity of Insect Glutathione S-transferases in Xenobiotic Adaptation. International Journal of Biological Sciences. 2022;18 15:5713-23. doi:10.7150/ijbs.77141.
19. Liu NY, Xu W, Dong SL, Zhu JY, Xu YX and Anderson A. Genome-wide analysis of ionotropic receptor gene repertoire in Lepidoptera with an emphasis on its functions of *Helicoverpa armigera*. Insect Biochem Mol Biol. 2018;99:37-53. doi:10.1016/j.ibmb.2018.05.005.
20. Srinivasan A, Giri AP and Gupta VS. Structural and functional diversities in lepidopteran serine proteases. Cellular & Molecular Biology Letters. 2006;11 1:132-54. doi:10.2478/s11658-006-0012-8.
21. Sun L, Mao TF, Zhang YX, Wu JJ, Bai JH, Zhang YN, et al. Characterization of candidate odorant-binding proteins and chemosensory proteins in the tea geometrid *Ectropis obliqua* Prout (Lepidoptera: Geometridae). Archives of Insect Biochemistry and Physiology. 2017;94 4 doi:10.1002/arch.21383.
22. Cohen ZP, Brevik K, Chen YH, Hawthorne DJ, Weibel BD and Schoville SD. Elevated rates of positive selection drive the evolution of pestiferousness in the Colorado potato beetle (*Leptinotarsa decemlineata*, Say). Molecular Ecology. 2021;30 1:237-54. doi:10.1111/mec.15703.
23. Hazzouri KM, Sudalaimuthuasari N, Kundu B, Nelson D, Al-Deeb MA, Le Mansour A, et al. The genome of pest Rhynchophorus ferrugineus reveals gene families important at the plant-beetle interface. Communications Biology. 2020;3 1:323. doi:10.1038/s42003-020-1060-8.
24. Li M, Yang X, Fan F, Ge Y, Hong D, Wang Z, et al. De novo genome assembly of *Bradysia cellarum* (Diptera: Sciaridae), a notorious pest in traditional special vegetables in China. Insect Molecular Biology. 2022;31 4:508-18. doi:10.1111/imb.12776.
25. Powell D, Groβe-Wilde E, Krokene P, Roy A, Chakraborty A, Löfstedt C, et al. A highly-contiguous genome assembly of the Eurasian spruce bark beetle, *Ips typographus*, provides insight into a major forest pest. Communications Biology. 2021;4 1:1059. doi:10.1038/s42003-021-02602-3.
26. Benson DA, Karsch-Mizrachi I, Lipman DJ, Ostell J, Rapp BA and Wheeler DL. GenBank. Nucleic Acids Res. 2000;28 1:15-8. doi:10.1093/nar/28.1.15.
27. Stahlke AR, Chang J, Chudalayandi S, Heu CC, Geib SM, Scheffler BE, et al. Chromosome-scale genome assembly of the pink bollworm, *Pectinophora gossypiella*, a global pest of cotton. bioRxiv. 2022.
28. Tabuloc CA, Lewald KM, Conner WR, Lee Y, Lee EK, Cain AB, et al. Sequencing of Tuta absoluta genome to develop SNP genotyping assays for species identification. Journal of Pest Science. 2019;92 4:1397-407. doi:10.1007/s10340-019-01116-6.
29. Andrews S. FastQC: a quality control tool for high throughput sequence data. Babraham Bioinformatics, Babraham Institute, Cambridge, United Kingdom, 2010.
30. Ranallo-Benavidez TR, Jaron KS and Schatz MC. GenomeScope 2.0 and Smudgeplot for reference-free profiling of polyploid genomes. Nature Communications. 2020;11 1:1432. doi:10.1038/s41467-020-14998-3.
31. Pfenninger M , Schönenbeck P, Schell T. ModEst: Accurate estimation of genome size from next generation sequencing data. Molecular Ecology Resources. 2021;1–11. doi.org/10.1111/1755-0998.13570
32. Cheng H, Concepcion GT, Feng X, Zhang H and Li H. Haplotype-resolved de novo assembly using phased assembly graphs with hifiasm. Nature Methods. 2021;18 2:170-5. doi:10.1038/s41592-020-01056-5.
33. Seppey M, Manni M and Zdobnov EM. BUSCO: Assessing Genome Assembly and Annotation Completeness. Methods in Molecular Biology. 2019;1962:227-45. doi:10.1007/978-1-4939-9173-0_14.

34. Simão FA, Waterhouse RM, Ioannidis P, Kriventseva EV and Zdobnov EM. BUSCO: assessing genome assembly and annotation completeness with single-copy orthologs. Bioinformatics. 2015;31 19:3210-2. doi:10.1093/bioinformatics/btv351.
35. Roach MJ, Schmidt SA and Borneman AR. Purge Haplotigs: allelic contig reassignment for third-gen diploid genome assemblies. BMC Bioinformatics. 2018;19 1:460. doi:10.1186/s12859-018-2485-7.
36. Li H. Minimap2: pairwise alignment for nucleotide sequences. Bioinformatics. 2018;34 18:3094-100. doi:10.1093/bioinformatics/bty191.
37. Challis R, Richards E, Rajan J, Cochrane G and Blaxter M. BlobToolKit – Interactive Quality Assessment of Genome Assemblies. G3 Genes|Genomes|Genetics. 2020;10 4:1361-74. doi:10.1534/g3.119.400908.
38. Flynn JM, Hubley R, Goubert C, Rosen J, Clark AG, Feschotte C, et al. RepeatModeler2 for automated genomic discovery of transposable element families. Proceedings of the National Academy of Sciences. 2020;117 17:9451-7. doi:10.1073/pnas.1921046117.
39. Smit A, Hubley R and Green P. RepeatMasker Open-4.0 [http://www. repeatmasker. org] Accessed September, 2020.: Accessed, 2015.
40. Jurka J. Repeats in genomic DNA: mining and meaning. Current Opinion in Structural Biology. 1998;8 3:333-7. doi:10.1016/s0959-440x(98)80067-5.
41. Barnett DW, Garrison EK, Quinlan AR, Strömberg MP and Marth GT. BamTools: a C++ API and toolkit for analyzing and managing BAM files. Bioinformatics. 2011;27 12:1691-2. doi:10.1093/bioinformatics/btr174.
42. Brůna T, Hoff KJ, Lomsadze A, Stanke M and Borodovsky M. BRAKER2: automatic eukaryotic genome annotation with GeneMark-EP+ and AUGUSTUS supported by a protein database. NAR Genom Bioinform. 2021;3 1:lqaa108. doi:10.1093/nargab/lqaa108.
43. Buchfink B, Xie C and Huson DH. Fast and sensitive protein alignment using DIAMOND. Nature Methods. 2015;12 1:59-60. doi:10.1038/nmeth.3176.
44. Hoff KJ, Lomsadze A, Borodovsky M and Stanke M. Whole-Genome Annotation with BRAKER. Methods in Molecular Biology. 2019;1962:65-95. doi:10.1007/978-1-4939-9173-0_5.
45. Lomsadze A, Burns PD and Borodovsky M. Integration of mapped RNA-Seq reads into automatic training of eukaryotic gene finding algorithm. Nucleic acids research. 2014;42 15:e119-e.
46. Stanke M, Schöffmann O, Morgenstern B and Waack S. Gene prediction in eukaryotes with a generalized hidden Markov model that uses hints from external sources. BMC Bioinformatics. 2006;7:62. doi:10.1186/1471-2105-7-62.
47. Li H, Handsaker B, Wysoker A, Fennell T, Ruan J, Homer N, et al. The sequence alignment/map format and SAMtools. Bioinformatics. 2009;25 16:2078-9.
48. Brůna T, Lomsadze A and Borodovsky M. GeneMark-EP+: eukaryotic gene prediction with self-training in the space of genes and proteins. NAR Genom Bioinform. 2020;2 2:lqaa026. doi:10.1093/nargab/lqaa026.
49. Camargo RA, Barbosa GO, Possignolo IP, Peres LE, Lam E, Lima JE, et al. RNA interference as a gene silencing tool to control *Tuta absoluta* in tomato (*Solanum lycopersicum*). PeerJ. 2016;4:e2673. doi:10.7717/peerj.2673.
50. Gabriel L, Hoff KJ, Brůna T, Borodovsky M and Stanke M. TSEBRA: transcript selector for BRAKER. BMC Bioinformatics. 2021;22 1:566. doi:10.1186/s12859-021-04482-0.
51. Caballero M and Wegrzyn J. gFACs: Gene Filtering, Analysis, and Conversion to Unify Genome Annotations Across Alignment and Gene Prediction Frameworks. Genomics, Proteomics & Bioinformatics. 2019;17 3:305-10. doi:https://doi.org/10.1016/j.gpb.2019.04.002.
52. Jones P, Binns D, Chang H-Y, Fraser M, Li W, McAnulla C, et al. InterProScan 5: genome-scale protein function classification. Bioinformatics. 2014;30 9:1236-40. doi:10.1093/bioinformatics/btu031.

53. Koskinen P, Törönen P, Nokso-Koivisto J and Holm L. PANNZER: high-throughput functional annotation of uncharacterized proteins in an error-prone environment. Bioinformatics. 2015;31 10:1544-52. doi:10.1093/bioinformatics/btu851.
54. Moriya Y, Itoh M, Okuda S, Yoshizawa AC and Kanehisa M. KAAS: an automatic genome annotation and pathway reconstruction server. Nucleic Acids Res. 2007;35 Web Server issue:W182-5. doi:10.1093/nar/gkm321.
55. Sohn JC, Regier JC, Mitter C, Adamski D, Landry JF, Heikkilae M, et al. Phylogeny and feeding trait evolution of the mega- diverse Gelechioidea (Lepidoptera: Obtectomera): new insight from 19 nuclear genes. Systematic Entomology. 2016;41 1:112-32.
56. Kawahara AY, Plotkin D, Espeland M, Meusemann K, Toussaint EFA, Donath A, et al. Phylogenomics reveals the evolutionary timing and pattern of butterflies and moths. Proceedings of the National Academy of Sciences. 2019;116 45:22657-63. doi:10.1073/pnas.1907847116.
57. Katoh K and Standley DM. MAFFT multiple sequence alignment software version 7: improvements in performance and usability. Molecular biology and evolution. 2013;30 4:772-80.
58. Minh BQ, Dang CC, Vinh LS and Lanfear R. QMaker: Fast and Accurate Method to Estimate Empirical Models of Protein Evolution. Systematic Biology. 2021;70 5:1046-60. doi:10.1093/sysbio/syab010.
59. Misof B, Liu S, Meusemann K, Peters RS, Donath A, Mayer C, et al. Phylogenomics resolves the timing and pattern of insect evolution. Science. 2014;346 6210:763-7. doi:10.1126/science.1257570.
60. Nguyen L-T, Schmidt HA, Von Haeseler A and Minh BQ. IQ-TREE: a fast and effective stochastic algorithm for estimating maximum-likelihood phylogenies. Molecular biology and evolution. 2015;32 1:268-74.
61. Kalyaanamoorthy S, Minh BQ, Wong TKF, von Haeseler A and Jermiin LS. ModelFinder: fast model selection for accurate phylogenetic estimates. Nature Methods. 2017;14 6:587-9. doi:10.1038/nmeth.4285.
62. Minh BQ, Nguyen MA and von Haeseler A. Ultrafast approximation for phylogenetic bootstrap. Molecular Biology and Evolution. 2013;30 5:1188-95. doi:10.1093/molbev/mst024.
63. Guindon S, Dufayard JF, Lefort V, Anisimova M, Hordijk W and Gascuel O. New algorithms and methods to estimate maximum-likelihood phylogenies: assessing the performance of PhyML 3.0. Systematic Biology. 2010;59 3:307-21. doi:10.1093/sysbio/syq010.
64. Sanderson, MJ. 2002. Estimating absolute rates of molecular evolution and divergence times: a penalized likelihood approach. Molecular Biology Evolution. 19 (1): 101-109.
65. Smith SA and O'Meara BC. treePL: divergence time estimation using penalized likelihood for large phylogenies. Bioinformatics. 2012;28 20:2689-90. doi:10.1093/bioinformatics/bts492.
66. Emms DM and Kelly S. OrthoFinder: phylogenetic orthology inference for comparative genomics. Genome Biology. 2019;20 1:238. doi:10.1186/s13059-019-1832-y.
67. Ganote C, Mendes F, Henschel R, Hahn M and Fulton B. Introducing CAFE: Computational Analysis of (gene) Family Evolution. Bioinformatics. 2018;22 10:1269–71.
68. Alexa A and Rahnenführer J. Gene set enrichment analysis with topGO. Bioconductor Improv. 2009;27:1-26.
69. Lee G-E, Han T, Park H, Qi M and Li H. A phylogeny of the subfamily Thiotrichinae (Lepidoptera: Gelechiidae) with a revision of the generic classification based on molecular and morphological analyses. Systematic Entomology. 2021;46 2:357-79. doi:https://doi.org/10.1111/syen.12466.
70. Gavrielatos M, Kyriakidis K, Spandidos DA and Michalopoulos I. Benchmarking of next and third generation sequencing technologies and their associated algorithms for *de novo* genome assembly. Molecular Medicine Reports. 2021;23 4 doi:10.3892/mmr.2021.11890.
71. Lang D, Zhang S, Ren P, Liang F, Sun Z, Meng G, et al. Comparison of the two up-to-date sequencing technologies for genome assembly: HiFi reads of Pacific Biosciences Sequel II

system and ultralong reads of Oxford Nanopore. GigaScience. 2020;9 12 doi:10.1093/gigascience/giaa123.

72. Han MV, Thomas GW, Lugo-Martinez J and Hahn MW. Estimating gene gain and loss rates in the presence of error in genome assembly and annotation using CAFE 3. Mol Biol Evol. 2013;30 8:1987-97. doi:10.1093/molbev/mst100.

73. Li R-T, Huang L-Q, Dong J-F and Wang C-Z. A moth odorant receptor highly expressed in the ovipositor is involved in detecting host-plant volatiles. eLife. 2020;9:e53706. doi:10.7554/eLife.53706.

74. Proffit M, Birgersson G, Bengtsson M, Reis R, Witzgall P and Lima E. Attraction and oviposition of *Tuta absoluta* females in response to tomato leaf volatiles. Journal of Chemical Ecology. 2011;37 6:565-74. doi:10.1007/s10886-011-9961-0.

75. Burton RL and Schuster DJ. Oviposition Stimulant for Tomato Pinworms1 from Surfaces of Tomato Plants2. Annals of the Entomological Society of America. 1981;74 5:512-5. doi:10.1093/aesa/74.5.512.

76. Fenemore P. Oviposition of potato tuber moth, Phthorimaea operculella Zell.(Lepidoptera: Gelechiidae); identification of host-plant factors influencing oviposition response. New Zealand Journal of Zoology. 1980;7 3:435-9.

77. Meisner J, Ascher KRS and Lavie D. Factors influencing the attraction to oviposition of the potato tuber moth, Gnorimoschema operculella Zell.1. Zeitschrift für Angewandte Entomologie. 1974;77 1-4:179-89. doi:https://doi.org/10.1111/j.1439-0418.1974.tb03245.x.

78. Tu YH, Cooper AJ, Teng B, Chang RB, Artiga DJ, Turner HN, et al. An evolutionarily conserved gene family encodes proton-selective ion channels. Science. 2018;359 6379:1047-50. doi:10.1126/science.aao3264.

79. Visalakshmi J and Johnson J. Studies on a leaf feeding caterpillar of sweet potato, *Brachmia gonvolvuli* Wlsm.(Gelechiidae: Lepidoptera). Agricultural research journal of Kerala. 1968;6 1.

80. Schuster D. Development of Tomato Pinworm (Lepodoptera: Gelechiidae) on Foliage of Selected Plant Species. The Florida Entomologist. 1989;72 1:216-9.

81. Andersson MN, Keeling CI and Mitchell RF. Genomic content of chemosensory genes correlates with host range in wood-boring beetles (Dendroctonus ponderosae, Agrilus planipennis, and Anoplophora glabripennis). BMC Genomics. 2019;20 1:690. doi:10.1186/s12864-019-6054-x.

82. Goldman-Huertas B, Mitchell RF, Lapoint RT, Faucher CP, Hildebrand JG and Whiteman NK. Evolution of herbivory in Drosophilidae linked to loss of behaviors, antennal responses, odorant receptors, and ancestral diet. Proceedings of the National Academy of Sciences. 2015;112 10:3026-31. doi:10.1073/pnas.1424656112.

83. Xu W, Papanicolaou A, Zhang H-J and Anderson A. Expansion of a bitter taste receptor family in a polyphagous insect herbivore. Scientific Reports. 2016;6 1:1-10.

84. Groen SC and Whiteman NK. Ecology and Evolution of Secondary Compound Detoxification Systems in Caterpillars. Caterpillars in the Middle: Tritrophic Interactions in a Changing World. Springer; 2022. p. 115-63.

85. Breeschoten T, van der Linden CFH, Ros VID, Schranz ME and Simon S. Expanding the Menu: Are Polyphagy and Gene Family Expansions Linked across Lepidoptera? Genome Biology and Evolution. 2022;14 1 doi:10.1093/gbe/evab283.

86. Heidel-Fischer HM and Vogel H. Molecular mechanisms of insect adaptation to plant secondary compounds. Current Opinion in Insect Science. 2015;8:8-14. doi:10.1016/j.cois.2015.02.004.

87. El-Kady H. Insecticide resistance in potato tuber moth Phthorimaea operculella Zeller in Egypt. The Journal of American Science. 2011;7 10:263-6.

88. Langa TP, Dantas KC, Pereira DL, de Oliveira M, Ribeiro LM and Siqueira HA. Basis and monitoring of methoxyfenozide resistance in the South American tomato pinworm *Tuta absoluta*. Journal of Pest Science. 2022;95 1:351-64.

89. Zibaee I. The expression profile of detoxifying enzyme of tomato leaf miner, *Tuta absoluta* Meyrik (Lepidoptera: Gelechiidae) to chlorpyrifos. Arthropods. 2016;5 2:77.

90. Terra WR and Ferreira C. Insect digestive enzymes: properties, compartmentalization and function. Comparative Biochemistry and Physiology Part B: Comparative Biochemistry. 1994;109 1:1-62.

91. Jalapathi SK, Jayaraj J, Shanthi M, Theradimani M, Venkatasamy B, Irulandi S, et al. Potential of Cry1Ac from Bacillus thuringiensis against the tomato pinworm, Tuta absoluta (Meyrick) (Gelechiidae: Lepidoptera). Egyptian Journal of Biological Pest Control. 2020;30 1:81. doi:10.1186/s41938-020-00283-4.

92. Mohammed A, Douches DS, Pett W, Grafius E, Coombs J, Liswidowati, et al. Evaluation of potato tuber moth (Lepidoptera: Gelechiidae) resistance in tubers of Bt-cry5 transgenic potato lines. Journal of Economic Entomology. 2000;93 2:472-6. doi:10.1603/0022-0493-93.2.472.

93. Seal DR and Leibee GL. Toxicity of Bacillus thuringiensis CRY1-type insecticidal toxin to geographically distant populations of tomato pinworm. The Florida Entomologist. 2003;86 2:222-4.

94. Dias RO, Via A, Brandão MM, Tramontano A and Silva-Filho MC. Digestive peptidase evolution in holometabolous insects led to a divergent group of enzymes in Lepidoptera. Insect Biochemistry and Molecular Biology. 2015;58:1-11. doi:10.1016/j.ibmb.2014.12.009.

95. Kumar R, Bhardwaj U, Kumar P and Mazumdar-Leighton S. Midgut serine proteases and alternative host plant utilization in *Pieris brassicae* L. Frontiers in Physiology. 2015;6:95. doi:10.3389/fphys.2015.00095.

96. Souza TP, Dias RO, Castelhano EC, Brandão MM, Moura DS and Silva-Filho MC. Comparative analysis of expression profiling of the trypsin and chymotrypsin genes from Lepidoptera species with different levels of sensitivity to soybean peptidase inhibitors. Comparative Biochemistry and Physiology Part B. 2016;196-197:67-73. doi:10.1016/j.cbpb.2016.02.007.

97. Bell HA, Fitches EC, Down RE, Ford L, Marris GC, Edwards JP, et al. Effect of dietary cowpea trypsin inhibitor (CpTI) on the growth and development of the tomato moth *Lacanobia oleracea* (Lepidoptera: Noctuidae) and on the success of the gregarious ectoparasitoid *Eulophus pennicornis* (Hymenoptera: Eulophidae). Pest Management Science. 2001;57 1:57-65. doi:10.1002/1526-4998(200101)57:1<57::AID-PS273>3.0.CO;2-4.

98. Gharekhani GH and Salek-Ebrahimi H. Life table parameters of the tomato leaf miner *Tuta absoluta* (Lepidoptera: Gelechiidae) on different tomato cultivars. Journal of Applied Entomology. 2014;107 5:1765-70. doi:10.1603/EC14059.

99. Golizadeh A, Esmaeili N, Razmjou J and Rafiee-Dastjerdi H. Comparative life tables of the potato tuberworm, *Phthorimaea operculella*, on leaves and tubers of different potato cultivars. Journal of Insect Science. 2014;14:42. doi:10.1093/jis/14.1.42.

100. Hemmati SA, Takalloo Z, Taghdir M, Mehrabadi M, Balalaei S, Moharramipour S, et al. The trypsin inhibitor pro-peptide induces toxic effects in Indianmeal moth, *Plodia interpunctella*. Pesticide Biochemistry and Physiology. 2021;171:104730. doi:10.1016/j.pestbp.2020.104730.

101. Dezordi FZ, Vasconcelos CRDS, Rezende AM and Wallau GL. In and Outs of Chuviridae Endogenous Viral Elements: Origin of a Potentially New Retrovirus and Signature of Ancient and Ongoing Arms Race in Mosquito Genomes. Front Genet. 2020;11:542437. doi:10.3389/fgene.2020.542437.

102. Xiong Y, Burke WD and Eickbush TH. Pao, a highly divergent retrotransposable element from *Bombyx mori* containing long terminal repeats with tandem copies of the putative R region. Nucleic Acids Research. 1993;21 9:2117-23. doi:10.1093/nar/21.9.2117.

103. Goic B, Vodovar N, Mondotte JA, Monot C, Frangeul L, Blanc H, et al. RNA-mediated interference and reverse transcription control the persistence of RNA viruses in the insect model *Drosophila*. Nature Immunology. 2013;14 4:396-403. doi:10.1038/ni.2542.

104. Moelling K, Broecker F, Russo G and Sunagawa S. RNase H As Gene Modifier, Driver of Evolution and Antiviral Defense. Frontiers in Microbiology. 2017;8:1745. doi:10.3389/fmicb.2017.01745.

105. Tassetto M, Kunitomi M, Whitfield ZJ, Dolan PT, Sánchez-Vargas I, Garcia-Knight M, et al. Control of RNA viruses in mosquito cells through the acquisition of vDNA and endogenous viral elements. Elife. 2019;8 doi:10.7554/eLife.41244.
106. Wu J, Wu C, Xing F, Cao L, Zeng W, Guo L, et al. Endogenous reverse transcriptase and RNase H-mediated antiviral mechanism in embryonic stem cells. Cell Research. 2021;31 9:998-1010. doi:10.1038/s41422-021-00524-7.
107. Zhu M, Pan J, Tong X, Qiu Q, Zhang X, Zhang Y, et al. BmCPV-Derived Circular DNA vcDNA-S7 Mediated by *Bombyx mori* Reverse Transcriptase (RT) Regulates BmCPV Infection. Frontiers in Immunology. 2022;13:861007. doi:10.3389/fimmu.2022.861007.
108. Dodonova SO, Prinz S, Bilanchone V, Sandmeyer S and Briggs JAG. Structure of the Ty3/Gypsy retrotransposon capsid and the evolution of retroviruses. Proceedings of the National Academy of Sciences. 2019;116 20:10048-57. doi:10.1073/pnas.1900931116.
109. Rohrmann GF. Baculoviruses, retroviruses, DNA transposons (piggyBac), and insect cells. Baculovirus Molecular Biology [Internet] 4th edition. National Center for Biotechnology Information (US); 2019.
110. Yan B, Ou H, Wei L, Wang X, Yu X, Liu J, et al. A chromosome-level genome assembly of *Ephestia elutella* (Hübner, 1796)(Lepidoptera: Pyralidae). Genome Biology and Evolution. 2021;13 8:evab114.
111. Weng Y-M, Pathour SR, Godfrey KR, Plotkin D, Parker BM, Wist T, Kawahara AY. Supporting data for "Evolutionary genomics of three agricultural pest moths reveals rapid evolution of host adaptation and immune-related genes" GigaScience Database. 2023; http://dx.doi.org/10.5524/102462.

Tables

Table 1. Assembly statistics of the three newly sequenced gelechiid moth species, compared to statistics of the published *Phthorimaea absoluta* v1 assembly. BUSCO results from the *Phthorimaea absoluta* v1 assembly have been re-analyzed using BUSCO v5.

	***Phthorimaea absoluta* v1**	***Phthorimaea absoluta* v2**	***Keiferia lycopersicella***	***Scrobipalpa atriplicella***
Number of contigs	51,398	688	61	7,092
Total length	906,539,853	652,703,157	443,647,192	301,148,843
GC content	38.11%	38.45%	38.86%	36.87%
Contig N50	97,121	1,614,219	14,556,016	51,599
Contig L50	1,787	116	12	1,805
genome BUSCO complete (C)	C:90.3%[S:67.1%,D:23.2%]	96.2%[S:82.5%,D:13.7%]	96.6%[S:95.5%,D:1.1%]	C:73.3%[S:69.7%,D:3.6%]
genome BUSCO fragmented (F)	3.4%	0.5%	0.7%	2.8%
genome BUSCO missing (M)	6.3%	3.3%	2.7%	23.9%
Repeat percentage	-	54.4%	48.22%	32.83%
number of protein coding genes	-	19,106	15,405	14,647
gene model BUSCO (C)	-	93.2%[S:75.9%,D:17.3%]	93.2%[S:91.7%,D:1.5%]	70.2%[S:60.6%,D:9.6%]
gene model BUSCO (F)	-	1.3%	0.7%	3.4%
gene model BUSCO (M)	-	5.5%	6.1%	26.4%
number of monoexonic genes	-	2,568	2,040	1,600
Reference	Tabuloc et al., 2019	this study	this study	this study

Table 2. Enriched GO terms from the rapidly evolving genes of the five gelechiid species in this study.

	GO term ID	Biological Function	P_{weight}	$P_{classic}$
K. lycopersicella	GO:0007275	multicellular organism development	5.52E-09	5.52E-09
	GO:0007304	chorion-containing eggshell formation	4.31E-14	4.31E-14
P. operculella	GO:0006313	transposition, DNA-mediated	0.0161611	0.0161611
	GO:0006508	proteolysis	4.955E-05	6.116E-05
	GO:0006979	response to oxidative stress	0.0028518	0.0028518
	GO:0015074	DNA integration	2.156E-06	2.156E-06
	GO:0050909	sensory perception of taste	6.504E-10	5.851E-09
P. absoluta	GO:0000723	telomere maintenance	0.046281	0.046281
	GO:0002224	toll-like receptor signaling pathway	0.023407	0.023407
	GO:0006313	transposition, DNA-mediated	0.0015755	0.0015755
	GO:0006508	proteolysis	0.0035861	0.0035861
	GO:0006955	immune response	0.046281	0.046281
	GO:0007275	multicellular organism development	3.635E-05	3.635E-05
	GO:0007304	chorion-containing eggshell formation	3.121E-08	3.121E-08
	GO:0010923	negative regulation of phosphatase activity	1.247E-06	1.247E-06
	GO:0048015	phosphatidylinositol-mediated signaling	0.046281	0.046281
P. gossypiella	GO:0006310	DNA recombination	0.0009534	0.0009534
	GO:0006334	nucleosome assembly	2.504E-29	2.504E-29
	GO:0006357	regulation of transcription by RNA polymerase II	0.03104	0.03104
	GO:0006418	tRNA aminoacylation for protein translation	0.0002912	0.0002912
	GO:0006486	protein glycosylation	0.0024002	0.0024002
	GO:0006915	apoptotic process	0.0006649	0.0006649
	GO:0007275	multicellular organism development	0.0099958	0.0099958
	GO:0007304	chorion-containing eggshell formation	0.0005445	0.0005445
	GO:0015074	DNA integration	5.079E-20	5.079E-20
	GO:0017121	plasma membrane phospholipid scrambling	2.236E-09	2.236E-09

Figure legends

Figure 1. (left) Maximum likelihood tree of four gelechiid species from a concatenated supermatrix analysis of 4,876 single-copy genes, presented alongside a color-coded number of rapidly evolving gene families (red: expanding, blue: contracting). The tree is rooted with *Hyposmocoma kahamanoa* (Gelechioidea: Cosmopterigidae). Nodes are labelled with branch supports (ultrafast bootstrap/SH-aLRT). (right) The list of rapidly evolving gene families that are associated with host plants includes a host compound-sensing gene family, 20 detoxification genes, and seven digestion-related genes. Numbers in color-coded cells represent repertoire size change in corresponding branches on the tree in (left), and gene-family functions are shown at the top of columns. The significant repertoire size changes are marked with outside borders on the cell. References supporting categorizations of gene function are provided in **Supplemental Table S5**.

Figure 1

Click here to access/download;Figure;Figure1.jpg

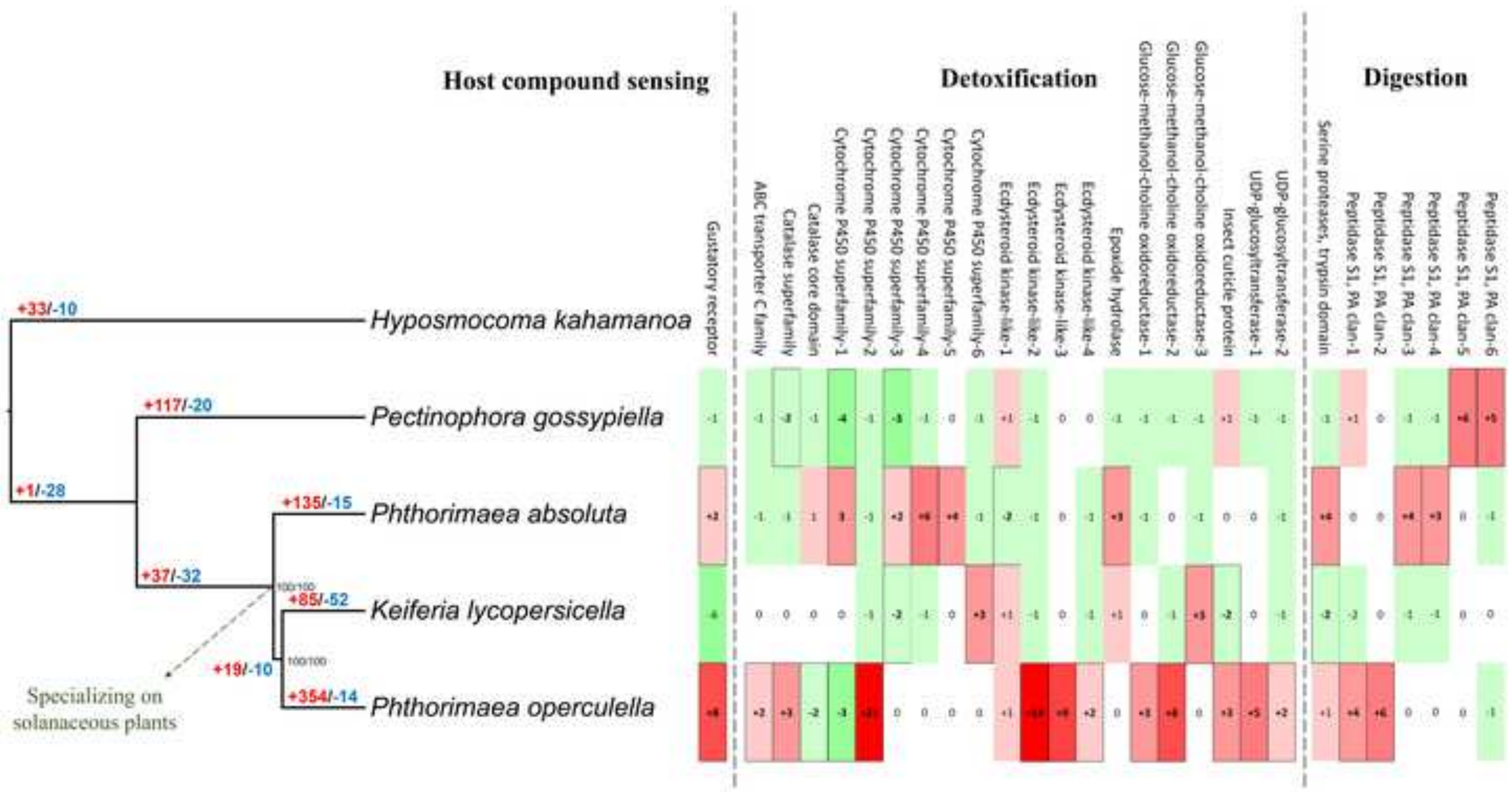

Figure S1

Click here to access/download
Supplementary Material
Figure_S1_GenomeScope.jpg

Figure S2

Click here to access/download
Supplementary Material
Figure_S2_Blobplots.jpg

Figure S3

Click here to access/download
Supplementary Material
Figure_S3_5sp_phylogeny.jpg

Table S1

Click here to access/download
Supplementary Material
Table_S1_gFACs_results.xlsx

Table S2

Click here to access/download
Supplementary Material
Table_S2_ModEst_results.xlsx

Table S3

Click here to access/download
Supplementary Material
Table_S3_list_of_rapidly_evolve_HOGs.xlsx

Table S4

Click here to access/download
Supplementary Material
Table_S4_immunity_HOGs.xlsx

Table S5

Click here to access/download
Supplementary Material
Table_S5_host_adaptation_HOGs.xlsx

Table S6

Click here to access/download
Supplementary Material
Table_S6_enriched_GO_summary.xlsx

Personal Cover

Click here to access/download;Personal Cover;Cover_resubmission_ymw.doc

UF | UNIVERSITY of FLORIDA

Florida Museum of Natural History
McGuire Center for Lepidoptera and Biodiversity

3215 Hull Rd.
Gainesville, FL 32611

August 01, 2023

Scott Edmunds
Chief Editor
GigaScience

Dear Dr. Edmunds,

Thank you for all the efforts to help us improve our manuscript entitled "*Evolutionary genomics of three agricultural pest moths reveals rapid evolution of host adaptation and immune-related genes*". To address the major concern about the poor quality of the genome of *Scrobipalpa aptatella*, we have tried to sequence more samples but unfortunately it still did not work. However, we followed the suggestion from both reviewers to remove this species from the gene family evolution analysis but keep it in this study for just phylogenetic analysis because we believe that it still can be useful data for other research. For all other suggestions from the reviewers, we've tried our best to address them to improve our manuscript. Please kindly consider our work for publication. Thank you very much for your consideration.

Sincerely,

Yi-Ming Weng
