# Supplementary figures and images for: Evolutionary genomics of three agricultural pest moths reveals rapid evolution of host adaptation and immune-related genes
Source: Gigascience. 2024 Jan 2;13:giad103. doi: 10.1093/gigascience/giad103 (PMC10759296; doi:10.1093/gigascience/giad103)

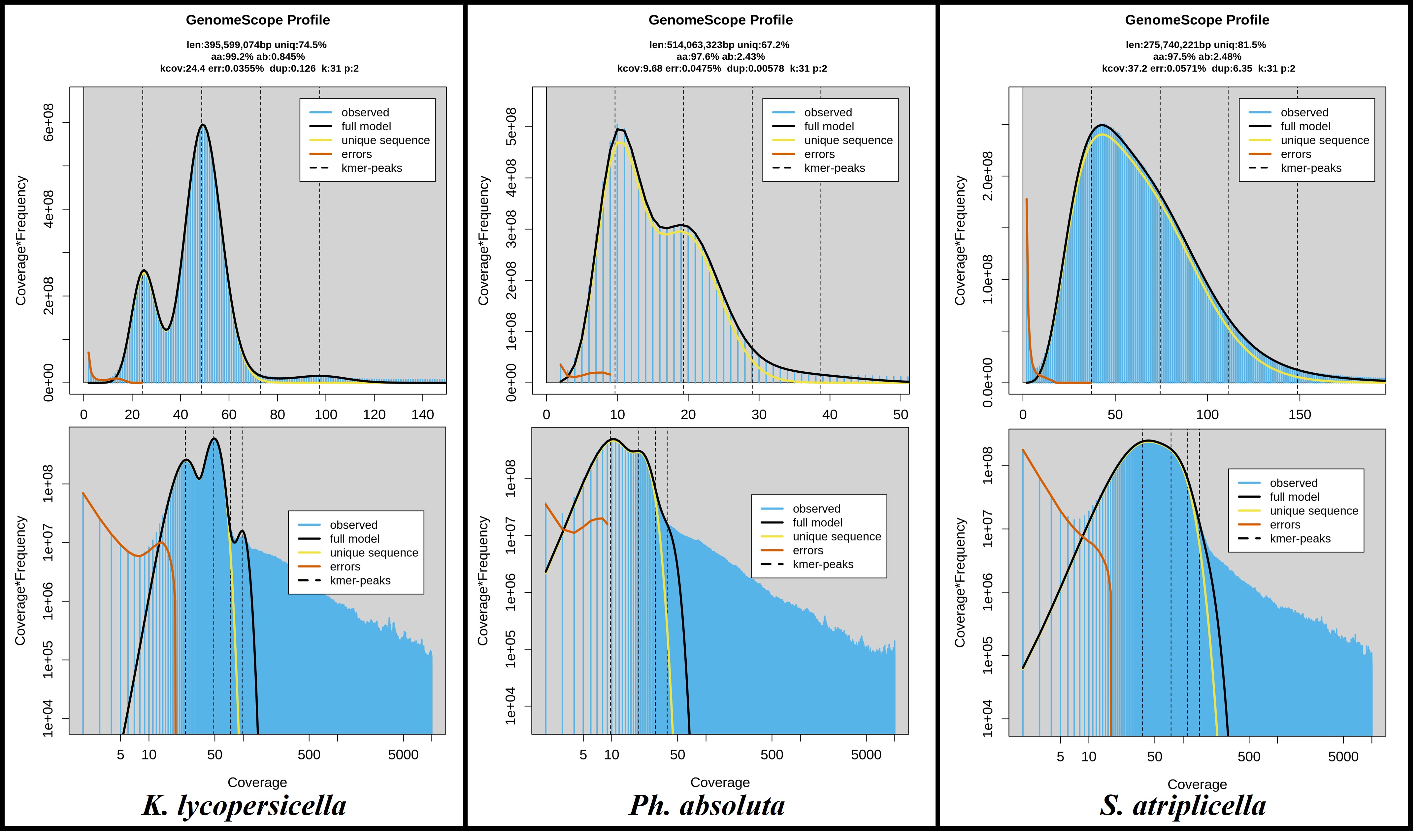

Supplement: giad103_Supplemental_Files [file giad103_supplemental_files.zip › Figure_S1_GenomeScope.jpg]

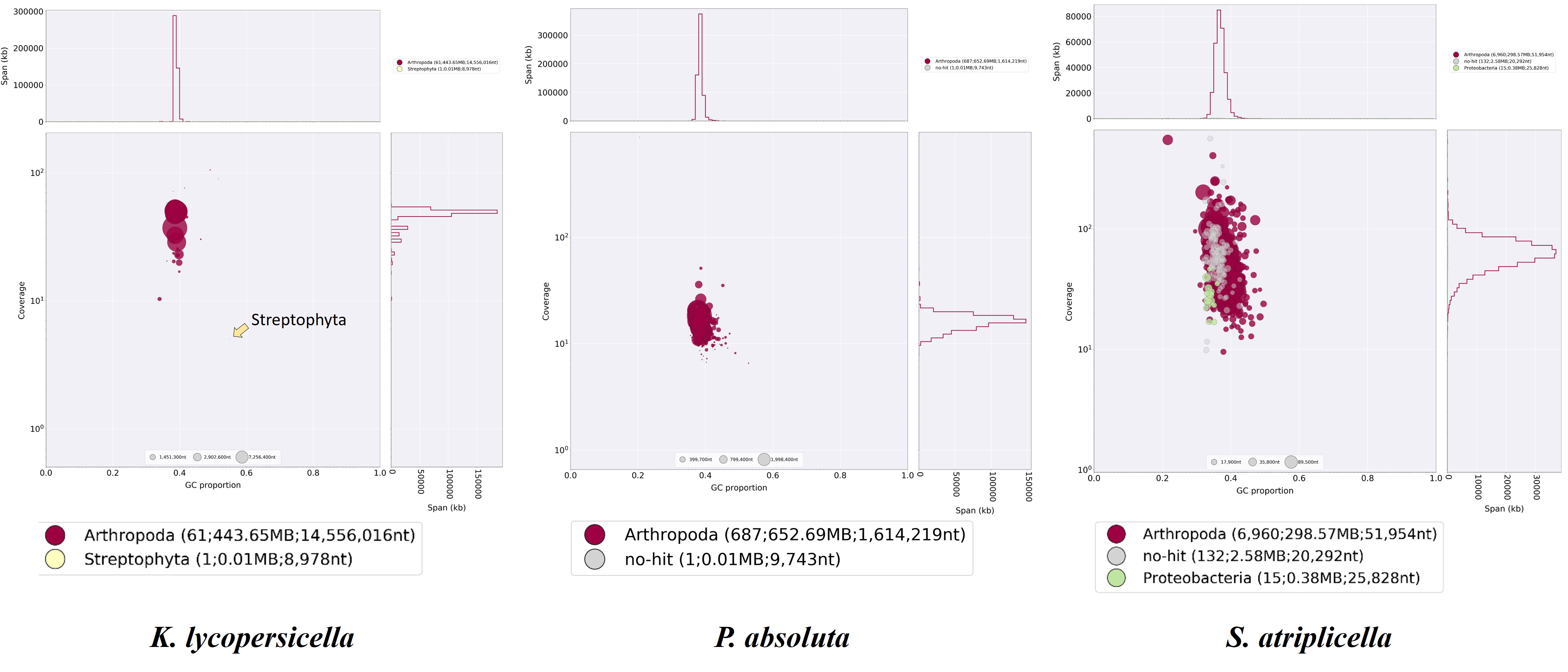

Supplement: giad103_Supplemental_Files [file giad103_supplemental_files.zip › Figure_S2_Blobplots.jpg]

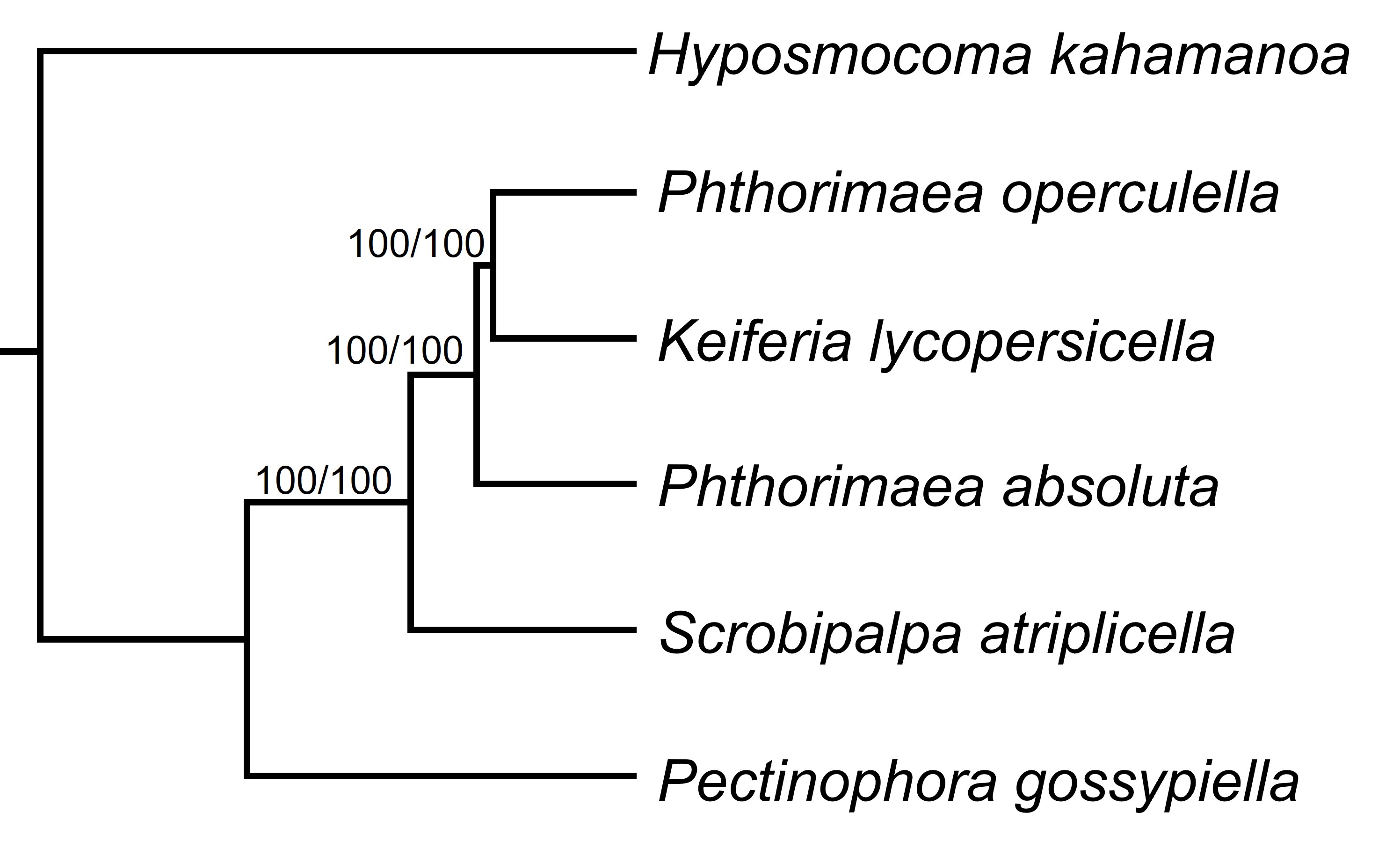

Supplement: giad103_Supplemental_Files [file giad103_supplemental_files.zip › Figure_S3_5sp_phylogeny.jpg]
