# Supplementary material for: Evolutionary genomics of three agricultural pest moths reveals rapid evolution of host adaptation and immune-related genes
Source: Gigascience. 2024 Jan 2;13:giad103. doi: 10.1093/gigascience/giad103 (PMC10759296; doi:10.1093/gigascience/giad103)
Supplement: giad103_Response_to_Reviewer_Comments_Original_Submission [file giad103_response_to_reviewer_comments_original_submission.pdf]

Author's Response To Reviewer Comments

Reviewer #1: The manuscript entitled "Evolutionary genomics of three agricultural pest moths reveals rapid evolution of host adaptation and immune-related genes" reported three genome assemblies of gelechiid moth species. All the three species are important agricultural pests and deciphering their genomes will provide a clue to the genetic background of their pest-relevant phenotypes and also some other ecological features. The three genomes are important additions to the insect genome database, and deserve full consideration for publication in GigaScience. However, there are several drawbacks that could influence the robustness of their main findings and need to be addressed before its acceptance.

The main and core issues are genome-size related: the estimated genome size of each species varied significantly from the final assemblies. A redundant genome assembly means multiple haplotig are retained due to a failure of the haplotypes' collapse; it is, therefore, hard to obtain correct inference in analyses related to gene contrast and expansion using assemblies that are mosaics of haplotype-fused contigs and haplotype-separated contigs. As the authors did not provide the number of individuals used for DNA extraction for each species, the influence of individual variation can also hardly be evaluated. If the authors believe the genome sizes estimated using GenomeScope and HiFi reads cannot represent true values, then they need to find reliable methods or datasets to obtain the correct ones.
Response: Thank you for the comment. All the assemblies are from sequences of single moth individual for each species. For genome size estimation, we believed that the assembled size is more accurate and the one from GenomeScope are underestimated. Two reasons we believe so. First, the genomes of these moth species have many repeats as we have seen the large number of reverse transcriptases and repeat-related genes in the genomes. When we use the kmer to estimate the genome size, the kmers with extremely high copy number will be excluded, even though they could be from the repeat regions, and thus the genome size will be underestimated when many repeats are in the genome. Second, we have applied haplotig purge pipeline to remove the redundant haplotigs, the BUSCO duplication rates were significantly reduced after the haplotig purge pipeline, with the completeness score remains consistent. We believe this indicates that the duplicated haplotigs are efficiently removed. We have added a sentence to clarify the potential source of this discrepancy and provide the number of samples for sequencing for each species in line 227-230 and line 94, respectively.

In Figure S1, it seems more reasonable, for K. lycopersicella, that the 50X represents the real kcov peak, while the first 25X peak comes from genome regions of high heterozygosity. Otherwise, maybe the authors can provide more likely explanations. The GC-depth plot (Figure S2) is of low resolution and blurred when zoomed in. Is that a peak of > 50X for K. lycopersicella as shown in Figure S2.
Response: Thank you. We changed the coverage from the heterozygous peak to homozygous peak so now it is 50, 20, and 36X. We have re-uploaded the figure with high resolution for each figure. The higher peak for the homozygous k-mer is likely because the sample was from the rearing colony and the moths are highly inbred. The highest coverage peak of K. lycopersicella in Figure S2 is around 50X but there are also many smaller peaks which are around 20-45.

The authors claimed that 3.8, 2.2, and 2.9 million PacBio HiFi reads were generated for the three species of K. lycopersicella, P. absoluta, and S. atriplicella, respectively. As the authors did not provide the quality information of the raw sequencing data, assuming an average library size (molecular length) of 10 kb, they obtained a total of 38, 22 and 29 Gb data for K. lycopersicella, P. absoluta, and S. atriplicella, respectively. If so, sequencing data of the three species reached ca. 100X coverage of depth, which is much higher than the Kmer coverage peak obtained using GenomeScope.
Response: Our ccs reads are relatively short, which are 5.5, 4.9, and 7.6 kbp for K. lycopersicella, P. absoluta, and S. atriplicella, respectively. Plus, we were reporting the heterozygous coverage which is misleading so that we have changed them to be homozygous coverage. We have added the related information to our results at line 217-219.

In addition, because of the amplification bias, I don't think the genome assembly of S. atriplicella can reach a high level of integrity using whatever methods. I would suggest the exclusion of S. atriplicella in any analyses related to gene count because of its incompleteness, which makes the relevant results and

conclusions untenable.
Response: Thank you for your suggestion. We agree that the incompleteness of the S. atriplicella assembly could possibly bias the results. We have removed the species from the gene family analyses, please see the updates in Table1, Figure 1, and Table S2-S5.

Reviewer #2: Review of Weng, Pathour et al. submission to GigaScience: Evolutionary genomics of three agricultural pest moths reveals rapid evolution of host adaptation and immune-related genes.

This manuscript describes the de novo genome assembly and annotation of three gelechiid moth species and places these assemblies alongside the (relatively scant) genomic resources publicly available for this agriculturally-relevant group. After annotation, orthology relationships are established and the authors investigate gene family expansion/contraction properties and perform an analysis of GO enrichment in

Major comments

The three genome assemblies presented here differ quite markedly in their quality and I am concerned that this aspect of the work is not given significant prominence/discussion as these differences may have an impact on the conclusions that are drawn. The best quality assembly is that of K lycopersicella, which has behaved as one would conventionally hope a HiFi dataset would-producing a complete and highly contiguous assembly that has required some further curation to remove redundancy. Slightly lower sequencing effort coupled with a larger genome has resulted in lower coverage for the P absoluta assembly, but it seems likely that the extreme heterozygosity in this sample is making a substantial contribution to the difficulties in producing a high-quality assembly here. The authors used higher coverage Illumina data to inform their purging of redundant allelic diversity from the assembly and this has been quite effective but it should be noted that the duplication level remains ~14% and this is likely to particularly influence the robustness of conclusions around gene family expansion and contraction (as the authors allude to in their discussion). Despite these limitations, the assembly presented here does appear to be an improvement on the previously published V1 assembly of this species. Most problematic is the S. atriplicella genome assembly- sample sequencing difficulties were noted and whole genome amplification was used to circumvent these. The GenomeScope k-mer profile (and the contrast between this and the other species) provides a visual summary of the limitations of this sequence library. The k-mer distribution peak is broad, with no clear distinction between haploid and diploid peaks. I cannot see that a coverage-based purging of redundant haplotigs would be effective here (and I note that the supplement details the upper and lower coverage thresholds but makes no mention of the homozygous and heterozygous peaks). The signal in the blobplot is also much more dispersed than the relatively tight GC/coverage clusters in the other two species. The resulting assembly is highly fragmented and shows BUSCO completeness stats <75% (whereas the other species exceed 95%). I would consider the quality of this genome to be sufficiently poor as to possibly compromise the comparative genomics analyses presented.
Response: Thank you for pointing this problem out with the clear explanations. We agree that the qualities of the assemblies will possibly confound the results of gene family evolution analysis. According to the suggestion (also suggested by another reviewer), we have rerun the analysis without S. atriplicella but keep P. absoluta in the analysis, with careful interpretations. The general trend remains with the rerun results with some minor gene count numbers changed. Please kindly find the update in Table1, Figure 1, and Table S2-S5.

The authors use concatenation of single copy orthologues to construct a phylogeny based on 4,876 single copy orthlogues using concatenation and coalescent approaches. I find the topology of this tree surprising- particularly the paraphyly of Phthorimaea and the low level of support for the K lycopersicella- Phthorimaea operculata node. Could the authors comment further on this? How does the phylogeny obtained here compare to metabarcoding data for these taxa, for example? I also wondered about the impact of the extreme heterozygosity in P. absoluta. One strategy for phylogeny reconstruction might be to run analyses with the P. absoluta genome before haplotype purging. This would presumably result in a smaller set of single-copy orthologues but might provide more robust phylogenetic signals.
Response: For the paraphyly of Phthorimaea, we believe that the phylogenetic relationship among the three species needs to be further tested with more species included. However, the taxonomic status of P. absoluta has been relatively unstable. Recently it was moved back to Phthorimaea from Tuta (actually, there are, possibly ongoing debate whether Phthorimaea absoluta should be placed in Phthorimaea or

Tuta). We have added a brief comment regarding this to the results since we know that the species sample are far from sufficient to discuss phylogeny and it is also not the focus of this study. Please see the changes at line 246-247. The relatively lower support from the astral tree is likely the result of many gene trees from BUSCO single copy genes showing distinct relationships of the three species. The uncertainties from the individual gene trees (either from the insufficient variants in the alignments or the quality of the sequences) were escalated with species tree approach. This has been recently reported as a short branch attraction by Liu et al (doi.org/10.3389/fevo.2023.1134764). Considering this, we have removed the astral tree from supplementary. We now have the supermatrix tree without S. atriplicella (the new tree for gene family evolution analysis) in the main text and move the supermatrix tree with S. atriplicella into supplementary. We understand that the heterozygosity and sequence quality could have affected the phylogeny but please allow us not to address to much since we might not have strong conclusion about this with the current species sampling. We hope the reviewer can be satisfied with this explanation.
Somewhat related to the previous comment, the authors note that there have been 5 previously published gelechiid assemblies and provide 3 references in the introduction to support this. One, I believe is the V1 assembly of P. absoluta (named Tuta absoluta in that publication, and perhaps this taxonomic distinction also requires some gloss). The other two references are the two genomes used in the comparative analyses. I initially thought some assemblies may not have been utilised but this doesn't seem to be the case. Could the authors clarify their counting? Are they including the species assembled here in this count?
Response: Thank you for pointing this out. When writing the manuscript (late 2022), we found 5 assemblies (1 for P. absoluta, 1 for K. lycopersicella, 1 for P. gossypiella, and 2 for P. operculella), where only 3 were qualified for gene family analysis. We thus used P. gossypiella and the more recent and better one of P. operculella for our analysis. However, we are aware that there are many assemblies got published in the first half of 2023, so we change our word from “five assemblies” to “a few assemblies” at line 81.

Please note that line numbering in the submitted manuscript would be helpful for review/editing.

Minor comments

Abstract:
The abstract is dominated by the rationale for sequencing these genomes. I would prefer more of the abstract to be devoted to the content of the manuscript (i.e. the genome properties, qualities and their content). One route to achieving this would be to condense the opening sentences, which are more fully expanded on in the introduction.
Response: Thank you. We have changed our abstract accordingly.
Why have the authors elected to use two-letter abbreviations for genera? It's my understanding that this is good practice when multiple species share the same final epithet, but this is not the case here.
Response: Thank you. We have changed the abbreviation of genera to be single letter.
Change "host compounding sensing" to "host compound sensing".
Response: We have corrected the spelling, thank you.
Introduction:
"….billion annual agricultural damage globally": Please provide a qualifying unit for billion. US dollars?
Response: Yes, we meant US dollars. We have added this information to the introduction at line 57.
"The three gelechiids all use of solanaceous plants as larval hosts, but…": Delete "of" or change to "all make use of"
Response: Thank you. We have corrected it accordingly (line 58).
"are found invading namy non-native regions": There's a typo (namy should be many) but perhaps it would be clearer to state that they "are invasive in many regions across the globe".
Response: Thank you. The correction has been made (line 60).
"Genes associated with sensing phytocompounds….": In these lists, the different receptors/protein types should be plural (you have a mix of singular and plural).
Response: Thank you. We have corrected it accordingly (line 67-68).
"…. with subsequent analyses such as comparative orthologous and gene family": suggest rephrasing this as "with subsequent comparative genomics analyses such as orthology, gene family evolution… "
Response: We have rephrased this sentence according to your suggestion (line 76).

Methods:

This section is generally clear, version numbers are recorded and the logic of the analyses set out are sound (but see note about incorporating data supplement methods into the main manuscript).
Are there additional parameters in the TopGO analysis that should be specified?
Response: Except for the algorithms and p-values, we kept all other parameters defaults (line 210).
Results:
Note coverage is conventionally reported with respect to a haploid representation of the genome i.e. K lycopersicella would be considered to be at 50x coverage and P absoluta coverage at 20x.
Response: Thank you. We have changed them to homozygous coverages (line 217).

Discussion:
There is some discussion of assembly challenges and the potential implications of these including acknowledgement of possible limitations, but I would encourage the authors to elaborate further on this- including some mention of high heterozygosity and how one deals effectively with this when analysing chimeric haploid genome representations, for example.
Response: Thank you. We have added a discussion on the possible outcomes of analysis gene evolution with highly duplicated genome. We believe that the best practice is to avoid this situation but, in our case, we have tried our best to suppress the duplication. We mentioned it at the discussion (line 301-308).
The discussion is dominated by discussion of the gene ontogeny enrichment analysis and puts forward plausible descriptions/hypotheses for how the gene repertoire may relate to adaptive traits such as host-plant sensing, detoxification and immunity.
Figure 1: The resolution of this figure in the rendered pdf is too poor for the content to be legible.
Response: We will resubmit out figures with high resolution images.

Data supplements:
I have edited the supplementary .docx file for language. Note that the figure for the genome size of Ph absoluta still needs to be added in. Please increase the size of the figures in order to make the embedded text readable. I encourage the authors to consider incorporating the supplemental methods presented here into the main body of the manuscript since the sections are not overly long and do contribute to the understanding of the assembly approach.
Response: We really appreciate your help editing the supplementary for language. However, we were not able to find the edits, but we have taken your suggestion to integrate the supplemental method to the main manuscript with more concise wording style (lines 130-135).
