# Supplementary material for: Evolutionary genomics of three agricultural pest moths reveals rapid evolution of host adaptation and immune-related genes
Source: Gigascience. 2024 Jan 2;13:giad103. doi: 10.1093/gigascience/giad103 (PMC10759296; doi:10.1093/gigascience/giad103)
Supplement: giad103_Response_to_Reviewer_Comments_Revision_1 [file giad103_response_to_reviewer_comments_revision_1.pdf]

# Author's Response To Reviewer Comments

Reviewer #1: The authors have addressed the concerns I raised in my first review. Most of them have been well addressed, a

The results of genomeScope (Figure S1) is really misleading. I would recommend you to use other programs or do a calculati
<20X, respectively. Genome size should be > 418Mb (20.9/50) and >539MB (10.78/20), respectively, which fit your final ass

Response: Thank you for the suggestions. We agree that this manual calculation is better than the estimates of GenomeScop
reanalyzed the kmer count (KMC) by relaxing the max count to be 10,000 counts and increasing the k-mer size from 21 to 3
atriplicella). Although this is still smaller than the assembled sizes (there are still some extremely high k-mer counts not inclu

Reviewer #2: The authors have taken on board the comments from the first round of review and I find the resulting manuscr

The discrepancy between GenomeScope genome size estimates and the recovered assembly remains. If there are no relevan
dataset. The paper also provides some comparisons between k-mer-based and flow cytometry/read-mapping estimates of ge
GenomeScope k-mer analysis on the Illumina dataset you used to support your coverage-binning for that one taxon? A plot c
(this is to some extent provided in the blobplot supplement, but I think there each contig has a single coverage value). Revie

Response: Thank you for your suggestions. We followed your suggestion using Pfenninger's tool (backmap) to estimate the g
detect the correct peak coverage for P. absoluta (should be around 19X-20X but it got 10X) and likely because of this it doub
from 21 to 31 to reduce the number of k-mer being truncated. This resulted in the estimates much closer to our assembled s
GenomeScope results and hope this modification can satisfy the reviewers' concern. We note, however, that the assembled s

# backmap results
K. lycopersicella:
Mapped nucleotides: 20.67Gb
Peak coverage: 49
Genome size estimate: 421.76Mb
Assembly length: 443.65Mb (105.19% of estimate)

P. absoluta
Mapped nucleotides: 10.38Gb
Peak coverage: 10
Genome size estimate: 1.04Gb
Assembly length: 652.70Mb (62.89% of estimate)

S. atriplicella
Mapped nucleotides: 18.94Gb
Peak coverage: 55
Genome size estimate: 344.43Mb
Assembly length: 301.53Mb (87.54% of estimate)

Pfenninger, M., Schönnenbeck, P., & Schell, T. (2022). ModEst: Accurate estimation of genome size from next generation sec
0998.13570&data=05%7C01%7Cyimingweng%40ufl.edu%7Ce05b2616068948d7720a08db9cc0114f%7C0d4da0f84a314d76
HieTkepR0%2BHlpRg%3D&reserved=0

Minor:
L132: "Illumina" not "illumina"
L219: change "removals" to "removal"
L319-20: perhaps "Based on these experiences, we would recommend optimisation of DNA extraction to enable native DNA t
L362: should be "are found to prefer tomato over potato"
L365 change "is resulted from" to "has resulted from"
L387: should "reverse transcriptome" be "reverse transcription"?

Note that the blobplot figure S3 still contains the "Ph" not "P" abbreviation

Response: Thank you very much for catching these. The corrections have been made accordingly.
