# Supplementary material for: Evolutionary genomics of three agricultural pest moths reveals rapid evolution of host adaptation and immune-related genes
Source: Gigascience. 2024 Jan 2;13:giad103. doi: 10.1093/gigascience/giad103 (PMC10759296; doi:10.1093/gigascience/giad103)
Supplement: giad103_Response_to_Reviewer_Comments_Revision_2 [file giad103_response_to_reviewer_comments_revision_2.pdf]

Author's Response To Reviewer Comments

Reviewer #2: Thank you for your response to the previous round of review. This last issue of the genome size is still a little tricky. The solution you have opted for is to revisit GenomeScope with a different kmer dataset and with an increase in the repetitive kmer threshold. This inflates your genome size estimates, but it isn't entirely clear to me that this is an appropriate inflation (the authors of GenomeScope discuss this in their publication). To be clear, I am not saying that it is inappropriate and I do not object to you reporting these statistics, but my preference in this case, where it seems that assembly size and genome size estimation are not going to converge, would be that the genome size estimates produced with all of these tools are reported. The reason for this is (1) these figures are very helpful metrics that end users can use when assessing assembly quality and (2) it raises awareness of assembly size estimates, how they can differ and what tools are available. If you prefer not to do this, I would recommend also displaying the log-scaled GenomeScope output as this would provide a better visualization of the high-count kmer portion of the dataset.
The ModEst/Backmap approach I think is valid and working. My understanding is that it takes the modal depth of coverage and the total amount of sequence data generated and extrapolates from there. For P. absoluta, you have so much heterozygosity that you would expect the modal coverage to be of a haploid contig, and so I believe it would be valid to halve that output figure.

Response: Thank you for the suggestions, we agree that reporting different estimates from different approaches can better depict the problem about discrepancy of the genome size estimation and increase the transparency. Here we follow reviewer's suggestion to 1) add the log scaled GenomeScope output in Figure S1, and 2) report the estimated genome sizes and sequence coverages from MODEST (backmap), with brief discussion on the results compared to that of GenomeScope. Please see the changes in [Methods] lines 123-124; [Results] lines 248-252 (and Table S2); [Discussion] lines 344-347.
