# Supplementary material for: Evolutionary genomics of three agricultural pest moths reveals rapid evolution of host adaptation and immune-related genes
Source: Gigascience. 2024 Jan 2;13:giad103. doi: 10.1093/gigascience/giad103 (PMC10759296; doi:10.1093/gigascience/giad103)
Supplement: giad103_Response_to_Reviewer_Comments_Revision_3 [file giad103_response_to_reviewer_comments_revision_3.pdf]

Author's Response To Reviewer Comments

This is the revised version for adding the citation to the GigaDB.
