# Supplementary material for: Evolutionary genomics of three agricultural pest moths reveals rapid evolution of host adaptation and immune-related genes
Source: Gigascience. 2024 Jan 2;13:giad103. doi: 10.1093/gigascience/giad103 (PMC10759296; doi:10.1093/gigascience/giad103)
Supplement: giad103_Reviewer_1_Report_Original_Submission — Shanlin Liu -- 3/20/2023 Reviewed [file giad103_reviewer_1_report_original_submission.pdf]

Reviewer Report

Title: Evolutionary genomics of three agricultural pest moths reveals rapid evolution of host adaptation and immune-related genes

Version: Original Submission **Date:** 3/20/2023

Reviewer name: Shanlin Liu

Reviewer Comments to Author:

The manuscript entitled "Evolutionary genomics of three agricultural pest moths reveals rapid evolution of host adaptation and immune-related genes" reported three genome assemblies of gelechiid moth species. All the three species are important agricultural pests and deciphering their genomes will provide a clue to the genetic background of their pest-relevant phenotypes and also some other ecological features. The three genomes are important additions to the insect genome database, and deserve full consideration for publication in GigaScience. However, there are several drawbacks that could influence the robustness of their main findings and need to be addressed before its acceptance.
The main and core issues are genome-size related: the estimated genome size of each species varied significantly from the final assemblies. A redundant genome assembly means multiple haplotig are retained due to a failure of the haplotypes' collapse; it is, therefore, hard to obtain correct inference in analyses related to gene contrast and expansion using assemblies that are mosaics of haplotype-fused contigs and haplotype-separated contigs. As the authors did not provide the number of individuals used for DNA extraction for each species, the influence of individual variation can also hardly be evaluated.
If the authors believe the genome sizes estimated using GenomeScope and HiFi reads cannot represent true values, then they need to find reliable methods or datasets to obtain the correct ones.
In Figure S1, it seems more reasonable, for K. lycopersicella, that the 50X represents the real kcov peak, while the first 25X peak comes from genome regions of high heterozygosity. Otherwise, maybe the authors can provide more likely explanations. The GC-depth plot (Figure S2) is of low resolution and blurred when zoomed in. Is that a peak of > 50X for K. lycopersicella as shown in Figure S2.
The authors claimed that 3.8, 2.2, and 2.9 million PacBio HiFi reads were generated for the three species of K. lycopersicella, Ph. absoluta, and S. atriplicella, respectively. As the authors did not provide the quality information of the raw sequencing data, assuming an average library size (molecular length) of 10 kb, they obtained a total of 38, 22 and 29 Gb data for K. lycopersicella, Ph. absoluta, and S. atriplicella, respectively. If so, sequencing data of the three species reached ca. 100X coverage of depth, which is much higher than the Kmer coverage peak obtained using GenomeScope.
In addition, because of the amplification bias, I don't think the genome assembly of S. atriplicella can reach a high level of integrity using whatever methods. I would suggest the exclusion of S. atriplicella in any analyses related to gene count because of its incompleteness, which makes the relevant results and conclusions untenable.

Methods

Are the methods appropriate to the aims of the study, are they well described, and are necessary controls included? Choose an item.

Conclusions

Are the conclusions adequately supported by the data shown? Choose an item.

Reporting Standards

Does the manuscript adhere to the journal's guidelines on minimum standards of reporting? Choose an item.

Choose an item.

Statistics

Are you able to assess all statistics in the manuscript, including the appropriateness of statistical tests used? Choose an item.

Quality of Written English

Please indicate the quality of language in the manuscript: Choose an item.

Declaration of Competing Interests

Please complete a declaration of competing interests, considering the following questions:

- Have you in the past five years received reimbursements, fees, funding, or salary from an organisation that may in any way gain or lose financially from the publication of this manuscript, either now or in the future?
- Do you hold any stocks or shares in an organisation that may in any way gain or lose financially from the publication of this manuscript, either now or in the future?
- Do you hold or are you currently applying for any patents relating to the content of the manuscript?
- Have you received reimbursements, fees, funding, or salary from an organization that holds or has applied for patents relating to the content of the manuscript?
- Do you have any other financial competing interests?
- Do you have any non-financial competing interests in relation to this paper?

If you can answer no to all of the above, write 'I declare that I have no competing interests' below. If your reply is yes to any, please give details below.

I declare that I have no competing interests

I agree to the open peer review policy of the journal. I understand that my name will be included on my report to the authors and, if the manuscript is accepted for publication, my named report including any attachments I upload will be posted on the website along with the authors' responses. I agree for my report to be made available under an Open Access Creative Commons CC-BY license (http://creativecommons.org/licenses/by/4.0/). I understand that any comments which I do not wish to be included in my named report can be included as confidential comments to the editors, which will not be published.

Choose an item.

To further support our reviewers, we have joined with Publons, where you can gain additional credit to further highlight your hard work (see: https://publons.com/journal/530/gigascience). On publication of this paper, your review will be automatically added to Publons, you can then choose whether or not to claim your Publons credit. I understand this statement.

Yes Choose an item.
