# Supplementary material for: Evolutionary genomics of three agricultural pest moths reveals rapid evolution of host adaptation and immune-related genes
Source: Gigascience. 2024 Jan 2;13:giad103. doi: 10.1093/gigascience/giad103 (PMC10759296; doi:10.1093/gigascience/giad103)
Supplement: giad103_Reviewer_1_Report_Revision_1 — Shanlin Liu -- 8/11/2023 Reviewed [file giad103_reviewer_1_report_revision_1.pdf]

Reviewer Report

Title: Evolutionary genomics of three agricultural pest moths reveals rapid evolution of host adaptation and immune-related genes

Version: Revision 1 **Date:** 8/11/2023

Reviewer name: Shanlin Liu

Reviewer Comments to Author:

The authors have addressed the concerns I raised in my first review. Most of them have been well addressed, and it is almost there. I only have one additional suggestion for your reference.
The results of genomeScope (Figure S1) is really misleading. I would recommend you to use other programs or do a calculation by hand instead. For example, you obtained sequencing data of a total of 20.9 Gb (3.8m*5.5k) and 10.78 Gb (2.2*4.9k) for K. lycopersicella and P. absoluta, respectively. Kmer analysis showed that the depth coverage is about <50X and <20X, respectively. Genome size should be > 418Mb (20.9/50) and >539MB (10.78/20), respectively, which fit your final assemblies much better than that calculated from genomeScope.

Methods

Are the methods appropriate to the aims of the study, are they well described, and are necessary controls included? Choose an item.

Conclusions

Are the conclusions adequately supported by the data shown? Choose an item.

Reporting Standards

Does the manuscript adhere to the journal's guidelines on minimum standards of reporting? Choose an item.

Choose an item.

Statistics

Are you able to assess all statistics in the manuscript, including the appropriateness of statistical tests used? Choose an item.

Quality of Written English

Please indicate the quality of language in the manuscript: Choose an item.

Declaration of Competing Interests

Please complete a declaration of competing interests, considering the following questions:

- Have you in the past five years received reimbursements, fees, funding, or salary from an organisation that may in any way gain or lose financially from the publication of this manuscript, either now or in the future?
- Do you hold any stocks or shares in an organisation that may in any way gain or lose financially from the publication of this manuscript, either now or in the future?
- Do you hold or are you currently applying for any patents relating to the content of the manuscript?
- Have you received reimbursements, fees, funding, or salary from an organization that holds or has applied for patents relating to the content of the manuscript?
- Do you have any other financial competing interests?
- Do you have any non-financial competing interests in relation to this paper?

If you can answer no to all of the above, write 'I declare that I have no competing interests' below. If your reply is yes to any, please give details below.

I declare that I have no competing interests

I agree to the open peer review policy of the journal. I understand that my name will be included on my report to the authors and, if the manuscript is accepted for publication, my named report including any attachments I upload will be posted on the website along with the authors' responses. I agree for my report to be made available under an Open Access Creative Commons CC-BY license (http://creativecommons.org/licenses/by/4.0/). I understand that any comments which I do not wish to be included in my named report can be included as confidential comments to the editors, which will not be published.

Choose an item.

To further support our reviewers, we have joined with Publons, where you can gain additional credit to further highlight your hard work (see: https://publons.com/journal/530/gigascience). On publication of this paper, your review will be automatically added to Publons, you can then choose whether or not to claim your Publons credit. I understand this statement.

Yes Choose an item.
