# Supplementary material for: Evolutionary genomics of three agricultural pest moths reveals rapid evolution of host adaptation and immune-related genes
Source: Gigascience. 2024 Jan 2;13:giad103. doi: 10.1093/gigascience/giad103 (PMC10759296; doi:10.1093/gigascience/giad103)
Supplement: giad103_Reviewer_2_Report_Original_Submission — Annabel Charlotte Whibley -- 4/10/2023 Reviewed [file giad103_reviewer_2_report_original_submission.pdf]

Reviewer Report

Title: Evolutionary genomics of three agricultural pest moths reveals rapid evolution of host adaptation and immune-related genes

Version: Original Submission **Date:** 4/10/2023

Reviewer name: Annabel Charlotte Whibley

Reviewer Comments to Author:

Review of Weng, Pathour et al. submission to GigaScience: Evolutionary genomics of three agricultural pest moths reveals rapid evolution of host adaptation and immune-related genes
This manuscript describes the de novo genome assembly and annotation of three gelechiid moth species and places these assemblies alongside the (relatively scant) genomic resources publicly available for this agriculturally-relevant group. After annotation, orthology relationships are established and the authors investigate gene family expansion/contraction properties and perform an analysis of GO enrichment in
Major comments
The three genome assemblies presented here differ quite markedly in their quality and I am concerned that this aspect of the work is not given significant prominence/discussion as these differences may have an impact on the conclusions that are drawn. The best quality assembly is that of K lycopersicella, which has behaved as one would conventionally hope a HiFi dataset would-producing a complete and highly contiguous assembly that has required some further curation to remove redundancy.
Slightly lower sequencing effort coupled with a larger genome has resulted in lower coverage for the P absoluta assembly, but it seems likely that the extreme heterozygosity in this sample is making a substantial contribution to the difficulties in producing a high-quality assembly here. The authors used higher coverage Illumina data to inform their purging of redundant allelic diversity from the assembly and this has been quite effective but it should be noted that the duplication level remains ~14% and this is likely to particularly influence the robustness of conclusions around gene family expansion and contraction (as the authors allude to in their discussion). Despite these limitations, the assembly presented here does appear to be an improvement on the previously published V1 assembly of this species.
Most problematic is the S. atriplicella genome assembly- sample sequencing difficulties were noted and whole genome amplification was used to circumvent these. The GenomeScope k-mer profile (and the contrast between this and the other species) provides a visual summary of the limitations of this sequence library. The k-mer distribution peak is broad, with no clear distinction between haploid and diploid peaks. I cannot see that a coverage-based purging of redundant haplotigs would be effective here (and I note that the supplement details the upper and lower coverage thresholds but makes no mention of the homozygous and heterozygous peaks). The signal in the blobplot is also much more dispersed than the relatively tight GC/coverage clusters in the other two species. The resulting assembly is highly fragmented and shows BUSCO completeness stats <75% (whereas the other species exceed 95%). I would consider the quality of this genome to be sufficiently poor as to possibly compromise the comparative genomics analyses presented.

The authors use concatenation of single copy orthologues to construct a phylogeny based on 4,876 single copy orthlogues using concatenation and coalescent approaches. I find the topology of this tree surprising- particularly the paraphyly of Phthorimaea and the low level of support for the K lycopersicella- Phthorimaea operculata node. Could the authors comment further on this? How does the phylogeny obtained here compare to metabarcoding data for these taxa, for example? I also wondered about the impact of the extreme heterozygosity in P. absoluta. One strategy for phylogeny reconstruction might be to run analyses with the P. absoluta genome before haplotype purging. This would presumably result in a smaller set of single-copy orthologues but might provide more robust phylogenetic signals.

Somewhat related to the previous comment, the authors note that there have been 5 previously published gelechiid assemblies and provide 3 references in the introduction to support this. One, I believe is the V1 assembly of P. absoluta (named Tuta absoluta in that publication, and perhaps this taxonomic distinction also requires some gloss). The other two references are the two genomes used in the comparative analyses. I initially thought some assemblies may not have been utilised but this doesn't seem to be the case. Could the authors clarify their counting? Are they including the species assembled here in this count?

Please note that line numbering in the submitted manuscript would be helpful for review/editing.

Minor comments

Abstract:

The abstract is dominated by the rationale for sequencing these genomes. I would prefer more of the abstract to be devoted to the content of the manuscript (i.e. the genome properties, qualities and their content). One route to achieving this would be to condense the opening sentences, which are more fully expanded on in the introduction.

Why have the authors elected to use two-letter abbreviations for genera? It's my understanding that this is good practice when multiple species share the same final epithet, but this is not the case here.

Change "host compounding sensing" to "host compound sensing".

Introduction:

"….billion annual agricultural damage globally": Please provide a qualifying unit for billion. US dollars?

"The three gelechiids all use of solanaceous plants as larval hosts, but…": Delete "of" or change to "all make use of"

"are found invading namy non-native regions": There's a typo (namy should be many) but perhaps it would be clearer to state that they "are invasive in many regions across the globe".

"Genes associated with sensing phytocompounds….": In these lists, the different receptors/protein types should be plural (you have a mix of singular and plural).

"…. with subsequent analyses such as comparative orthologous and gene family": suggest rephrasing this as "with subsequent comparative genomics analyses such as orthology, gene family evolution… "

Methods:

This section is generally clear, version numbers are recorded and the logic of the analyses set out are sound (but see note about incorporating data supplement methods into the main manuscript).

Are there additional parameters in the TopGO analysis that should be specified?

Results:

Note coverage is conventionally reported with respect to a haploid representation of the genome i.e. K

lycopersicella would be considered to be at 50x coverage and P absoluta coverage at 20x.
Discussion:
There is some discussion of assembly challenges and the potential implications of these including acknowledgement of possible limitations, but I would encourage the authors to elaborate further on this-including some mention of high heterozygosity and how one deals effectively with this when analysing chimeric haploid genome representations, for example.
The discussion is dominated by discussion of the gene ontogeny enrichment analysis and puts forward plausible descriptions/hypotheses for how the gene repertoire may relate to adaptive traits such as host-plant sensing, detoxification and immunity.
Figure 1: The resolution of this figure in the rendered pdf is too poor for the content to be legible.
Data supplements:
I have edited the supplementary .docx file for language. Note that the figure for the genome size of Ph absoluta still needs to be added in. Please increase the size of the figures in order to make the embedded text readable. I encourage the authors to consider incorporating the supplemental methods presented here into the main body of the manuscript since the sections are not overly long and do contribute to the understanding of the assembly approach.

Methods

Are the methods appropriate to the aims of the study, are they well described, and are necessary controls included? Choose an item.

Conclusions

Are the conclusions adequately supported by the data shown? Choose an item.

Reporting Standards

Does the manuscript adhere to the journal's guidelines on [minimum standards of reporting?] Choose an item.

Choose an item.

Statistics

Are you able to assess all statistics in the manuscript, including the appropriateness of statistical tests used? Choose an item.

Quality of Written English

Please indicate the quality of language in the manuscript: Choose an item.

Declaration of Competing Interests

Please complete a declaration of competing interests, considering the following questions:

- Have you in the past five years received reimbursements, fees, funding, or salary from an organisation that may in any way gain or lose financially from the publication of this manuscript, either now or in the future?
- Do you hold any stocks or shares in an organisation that may in any way gain or lose financially from the publication of this manuscript, either now or in the future?
- Do you hold or are you currently applying for any patents relating to the content of the manuscript?
- Have you received reimbursements, fees, funding, or salary from an organization that holds or has applied for patents relating to the content of the manuscript?
- Do you have any other financial competing interests?
- Do you have any non-financial competing interests in relation to this paper?

If you can answer no to all of the above, write 'I declare that I have no competing interests' below. If your reply is yes to any, please give details below.

I declare that I have no competing interests

I agree to the open peer review policy of the journal. I understand that my name will be included on my report to the authors and, if the manuscript is accepted for publication, my named report including any attachments I upload will be posted on the website along with the authors' responses. I agree for my report to be made available under an Open Access Creative Commons CC-BY license (http://creativecommons.org/licenses/by/4.0/). I understand that any comments which I do not wish to be included in my named report can be included as confidential comments to the editors, which will not be published.

Choose an item.

To further support our reviewers, we have joined with Publons, where you can gain additional credit to further highlight your hard work (see: https://publons.com/journal/530/gigascience). On publication of this paper, your review will be automatically added to Publons, you can then choose whether or not to claim your Publons credit. I understand this statement.

Yes Choose an item.
