# Supplementary material for: Evolutionary genomics of three agricultural pest moths reveals rapid evolution of host adaptation and immune-related genes
Source: Gigascience. 2024 Jan 2;13:giad103. doi: 10.1093/gigascience/giad103 (PMC10759296; doi:10.1093/gigascience/giad103)
Supplement: giad103_Reviewer_2_Report_Revision_1 — Annabel Charlotte Whibley -- 8/13/2023 Reviewed [file giad103_reviewer_2_report_revision_1.pdf]

Reviewer Report

Title: Evolutionary genomics of three agricultural pest moths reveals rapid evolution of host adaptation and immune-related genes

Version: Revision 1 **Date:** 8/13/2023

Reviewer name: Annabel Charlotte Whibley

Reviewer Comments to Author:

The authors have taken on board the comments from the first round of review and I find the resulting manuscript much improved. These genome assemblies are useful community resources and a more nuanced discussion of their quality and features will better enable their appropriate usage.
The discrepancy between GenomeScope genome size estimates and the recovered assembly remains. If there are no relevant flow cytometry estimates of genome size available to use as a comparison, you could consider using the tool described in Pfenniger et al (2022; full reference below) to obtain an estimate of genome size based on modal coverage in your dataset. The paper also provides some comparisons between k-mer-based and flow cytometry/read-mapping estimates of genome size that may support your statements about the source of the discrepancy (though your differences are more extreme than many of the examples they present). There may be some bias in your HiFi dataset- did you ever run the GenomeScope k-mer analysis on the Illumina dataset you used to support your coverage-binning for that one taxon? A plot of the distribution of binned contig coverage might be a useful support for the assertion that there is not extensive retained duplication in the purged assemblies (this is to some extent provided in the blobplot supplement, but I think there each contig has a single coverage value). Reviewer 1 has quite carefully set out why this is of key importance for your gene family analyses.
Pfenninger, M., SchÃ¶nnenbeck, P., & Schell, T. (2022). ModEst: Accurate estimation of genome size from next generation sequencing data. Molecular Ecology Resources, 22, 1454-1464. https://doi.org/10.1111/1755-0998.13570
Minor:
L132: "Illumina" not "illumina"
L219: change "removals" to "removal"
L319-20: perhaps "Based on these experiences, we would recommend optimisation of DNA extraction to enable native DNA to be sequenced over the whole genome amplification route we followed in with this challenging sample.
L362: should be "are found to prefer tomato over potato"
L365 change "is resulted from" to "has resulted from"
L387: should "reverse transcriptome" be "reverse transcription"?
Note that the blobplot figure S3 still contains the "Ph" not "P" abbreviation

Methods

Are the methods appropriate to the aims of the study, are they well described, and are necessary controls included? Choose an item.

Conclusions

Are the conclusions adequately supported by the data shown? Choose an item.

Reporting Standards

Does the manuscript adhere to the journal's guidelines on minimum standards of reporting? Choose an item.

Choose an item.

Statistics

Are you able to assess all statistics in the manuscript, including the appropriateness of statistical tests used? Choose an item.

Quality of Written English

Please indicate the quality of language in the manuscript: Choose an item.

Declaration of Competing Interests

Please complete a declaration of competing interests, considering the following questions:

- Have you in the past five years received reimbursements, fees, funding, or salary from an organisation that may in any way gain or lose financially from the publication of this manuscript, either now or in the future?
- Do you hold any stocks or shares in an organisation that may in any way gain or lose financially from the publication of this manuscript, either now or in the future?
- Do you hold or are you currently applying for any patents relating to the content of the manuscript?
- Have you received reimbursements, fees, funding, or salary from an organization that holds or has applied for patents relating to the content of the manuscript?
- Do you have any other financial competing interests?
- Do you have any non-financial competing interests in relation to this paper?

If you can answer no to all of the above, write 'I declare that I have no competing interests' below. If your reply is yes to any, please give details below.

I declare that I have no competing interests

I agree to the open peer review policy of the journal. I understand that my name will be included on my report to the authors and, if the manuscript is accepted for publication, my named report including any attachments I upload will be posted on the website along with the authors' responses. I agree for my report to be made available under an Open Access Creative Commons CC-BY license (http://creativecommons.org/licenses/by/4.0/). I understand that any comments which I do not wish to be included in my named report can be included as confidential comments to the editors, which will not be published.

Choose an item.

To further support our reviewers, we have joined with Publons, where you can gain additional credit to further highlight your hard work (see: https://publons.com/journal/530/gigascience). On publication of this paper, your review will be automatically added to Publons, you can then choose whether or not to claim your Publons credit. I understand this statement.

Yes Choose an item.
