# Supplementary material for: Evolutionary genomics of three agricultural pest moths reveals rapid evolution of host adaptation and immune-related genes
Source: Gigascience. 2024 Jan 2;13:giad103. doi: 10.1093/gigascience/giad103 (PMC10759296; doi:10.1093/gigascience/giad103)
Supplement: giad103_Reviewer_2_Report_Revision_2 — Annabel Charlotte Whibley -- 9/11/2023 Reviewed [file giad103_reviewer_2_report_revision_2.pdf]

Reviewer Report

Title: Evolutionary genomics of three agricultural pest moths reveals rapid evolution of host adaptation and immune-related genes

Version: Revision 2 **Date:** 9/11/2023

Reviewer name: Annabel Charlotte Whibley

Reviewer Comments to Author:

Thank you for your response to the previous round of review. This last issue of the genome size is still a little tricky. The solution you have opted for is to revisit GenomeScope with a different kmer dataset and with an increase in the repetitive kmer threshold. This inflates your genome size estimates, but it isn't entirely clear to me that this is an appropriate inflation (the authors of GenomeScope discuss this in their publication). To be clear, I am not saying that it is inappropriate and I do not object to you reporting these statistics, but my preference in this case, where it seems that assembly size and genome size estimation are not going to converge, would be that the genome size estimates produced with all of these tools are reported. The reason for this is (1) these figures are very helpful metrics that end users can use when assessing assembly quality and (2) it raises awareness of assembly size estimates, how they can differ and what tools are available. If you prefer not to do this, I would recommend also displaying the log-scaled GenomeScope output as this would provide a better visualization of the high-count kmer portion of the dataset.
The ModEst/Backmap approach I think is valid and working. My understanding is that it takes the modal depth of coverage and the total amount of sequence data generated and extrapolates from there. For P. absoluta, you have so much heterozygosity that you would expect the modal coverage to be of a haploid contig, and so I believe it would be valid to halve that output figure.

Methods

Are the methods appropriate to the aims of the study, are they well described, and are necessary controls included? Choose an item.

Conclusions

Are the conclusions adequately supported by the data shown? Choose an item.

Reporting Standards

Does the manuscript adhere to the journal's guidelines on [minimum standards of reporting?](#) Choose an item.

Choose an item.

Statistics

Are you able to assess all statistics in the manuscript, including the appropriateness of statistical tests used? Choose an item.

Quality of Written English

Please indicate the quality of language in the manuscript: Choose an item.

Declaration of Competing Interests

Please complete a declaration of competing interests, considering the following questions:

- Have you in the past five years received reimbursements, fees, funding, or salary from an organisation that may in any way gain or lose financially from the publication of this manuscript, either now or in the future?
- Do you hold any stocks or shares in an organisation that may in any way gain or lose financially from the publication of this manuscript, either now or in the future?
- Do you hold or are you currently applying for any patents relating to the content of the manuscript?
- Have you received reimbursements, fees, funding, or salary from an organization that holds or has applied for patents relating to the content of the manuscript?
- Do you have any other financial competing interests?
- Do you have any non-financial competing interests in relation to this paper?

If you can answer no to all of the above, write 'I declare that I have no competing interests' below. If your reply is yes to any, please give details below.

I declare that I have no competing interests

I agree to the open peer review policy of the journal. I understand that my name will be included on my report to the authors and, if the manuscript is accepted for publication, my named report including any attachments I upload will be posted on the website along with the authors' responses. I agree for my report to be made available under an Open Access Creative Commons CC-BY license (http://creativecommons.org/licenses/by/4.0/). I understand that any comments which I do not wish to be included in my named report can be included as confidential comments to the editors, which will not be published.

Choose an item.

To further support our reviewers, we have joined with Publons, where you can gain additional credit to further highlight your hard work (see: https://publons.com/journal/530/gigascience). On publication of this paper, your review will be automatically added to Publons, you can then choose whether or not to claim your Publons credit. I understand this statement.

Yes Choose an item.
